# Synthesis and Biological Evaluation of 2-Substituted Quinazolin-4(3*H*)-Ones with Antiproliferative Activities

**DOI:** 10.3390/molecules28237912

**Published:** 2023-12-02

**Authors:** Maria Karelou, Dionysis Kampasis, Amalia D. Kalampaliki, Leentje Persoons, Andreas Krämer, Dominique Schols, Stefan Knapp, Steven De Jonghe, Ioannis K. Kostakis

**Affiliations:** 1 Department of Pharmacy, Division of Pharmaceutical Chemistry, National and Kapodistrian University of Athens, Panepistimiopolis, Zografou, 15771 Athens, Greece; marykar@pharm.uoa.gr (M.K.); dionisiskamp@pharm.uoa.gr (D.K.); akalampa@pharm.uoa.gr (A.D.K.); 2 Department of Microbiology, Immunology and Transplantation, Rega Institute for Medical Research, Laboratory of Virology and Chemotherapy, KU Leuven, Herestraat 49, P.O. Box 1043, 3000 Leuven, Belgium; leentje.persoons@kuleuven.be (L.P.); dominique.schols@kuleuven.be (D.S.); steven.dejonghe@kuleuven.be (S.D.J.); 3 Institute for Pharmaceutical Chemistry, Department of Biochemistry, Chemistry and Pharmacy, Goethe University Frankfurt, Max-von-Laue-Straße 9, 60438 Frankfurt, Germany; kraemer@pharmchem.uni-frankfurt.de (A.K.); knapp@pharmchem.uni-frankfurt.de (S.K.); 4 Structural Genomics Consortium, Buchmann Institute for Molecular Life Sciences, Goethe University Frankfurt, Max-von-Laue-Straße 15, 60438 Frankfurt, Germany

**Keywords:** quinazolines, synthesis, anti-proliferative activity, kinase inhibition

## Abstract

Sixteen new 2-substituted quinazolines were synthesized using a straightforward methodology starting from 2-methoxybezoic acid or 3-methoxy-2-naphthoic acid. The anti-proliferative activity of the target compounds was evaluated against nine cancer cell lines. Additionally, all the compounds were screened for their potency and selectivity against a panel of 109 kinases and four bromodomains, using Differential Scanning Fluorimetry (DSF). Compound **17** bearing a 2-methoxyphenyl substitution along with a basic side chain displayed a remarkable profile against the majority of the tested cell lines.

## 1. Introduction

Cancer is a complex disease that arises from the accumulation of genetic mutations and aberrant cellular signaling. The uncontrolled proliferation and metastasis of cancer cells are the hallmarks of this disease. Among the many different classes of chemotherapeutic agents used against malignant diseases, quinazoline derivatives have been extensively studied as anti-cancer compounds [1,2,3]. Furthermore, this scaffold has been used as a lead compound for the synthesis of various analogues [4,5,6,7].

Characterized by their quinazoline ring structure, these compounds have emerged as potent modulators of key cellular signaling pathways, offering promise for the development of selective and targeted interventions against cancer [8,9,10]. By inhibiting tyrosine kinases, enzymes that play a central role in cell growth and differentiation, quinazolinone-based inhibitors hold the potential to disrupt the aberrant signaling cascades that drive tumorigenesis. Their ability to target specific kinases associated with different cancer types highlights their importance in the pursuit of personalized therapeutic strategies [3,11,12]. 

**Figure 1:**
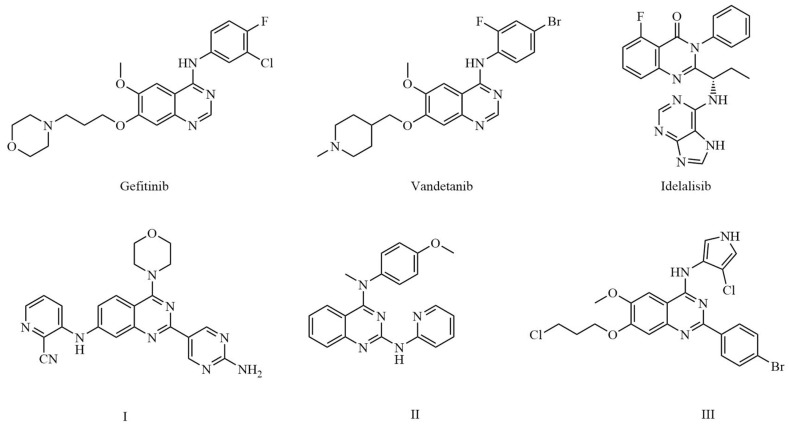
Structures of Gefitinib, Vandetanib, Idelalisib, and other known quinazoline kinase inhibitors (**I**–**III**).

Notable examples for approved and clinically used quinazoline-based drugs are Gefitinib [13] and the muti targeted kinase inhibitor Vandetanib [14], which have significantly impacted the treatment of non-small-cell lung cancer (NSCLC) by targeting, among others, the epidermal growth factor receptor (EGFR) (Figure 1). Following extensive Structure–Activity Relationship (SAR) studies in this class of compounds, it has been proposed that the substitution with one or two polar side chains and a substituted aniline plays an important role in their activity and selectivity as well as for optimizing the ADME (Absorption, Distribution, Metabolism, and Excretion) profile. Furthermore, Idelalisib, a PI3Kδ inhibitor, is approved for the treatment of chronic lymphocytic leukemia [15,16]. 

Beyond their role as kinase inhibitors, quinazolinones possess a rich array of capabilities that extend their utility in oncology. They have the potential to induce apoptosis in cancer cells, a process that triggers programmed cell death and is vital for slowing tumor growth [17,18]. Compounds I and II (Figure 1) are examples of known quinazoline analogs that inhibit tubulin polymerization. Recent studies show that 2-aryl-substituted quinazolines show moderate antiproliferative potency against various cell lines. Many other quinazoline analogs are under investigation for their antiproliferative activity [1,2,19,20,21].

In an effort to explore the optimal structural requirements for 2-aryl-substituted quinazolines, we designed a number of new analogs and present herein their synthesis and biological evaluation. In the new derivatives, a substituted phenyl or naphthyl ring is incorporated at position 2 of the quinazolinone moiety. In addition, a basic side chain was positioned at C8 aiming to identify the optimal structural requirements for biological activity. Furthermore, we modulated the aminoalkyl side chain to identify the optimal moiety for interaction with the hinge region of kinases, resulting in the preparation of various analogs with different aminoalkyl chains.

## 2. Results and Discussion

### 2.1. Chemistry

**Scheme 1:**
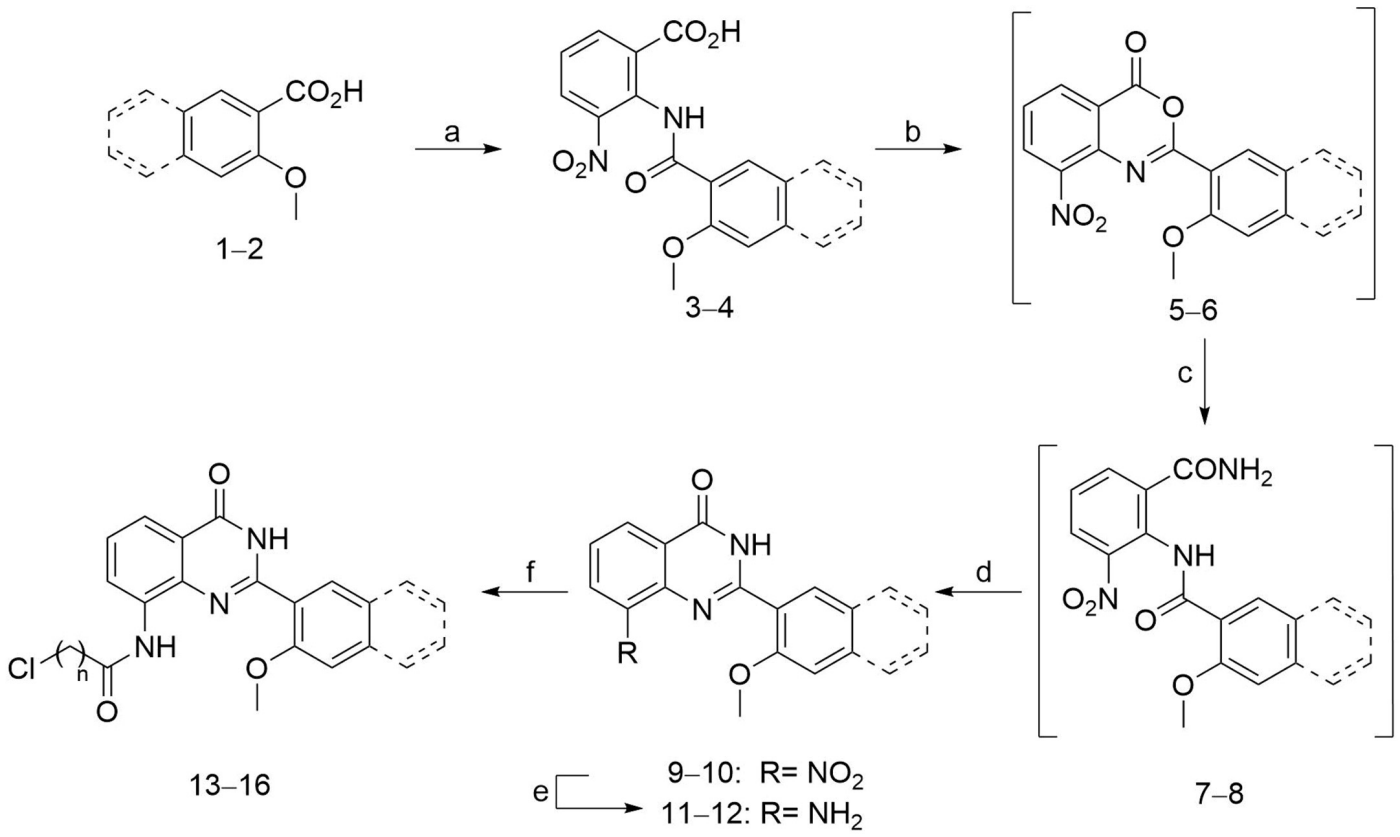
Reagents and conditions: (**a**) ACN, Et_3_N, ClCO_2_Et, 30 min, 0 °C/2-amino-3-nitrobenzoic acid, Na_2_CO_3_, 50 °C, 24 h; (**b**) (CH_3_CO)_2_O, reflux, 2 h; (**c**) NH_3_, THF, 12 h; (**d**) 5% aq. NaOH sol., reflux, 10 min; (**e**) EtOH abs., H_2_, Pd/C, 50 psi, 4 h; (**f**) **13–14**: ClCOCH_2_Cl, Na_2_CO_3_, THF, rt, 15 min; **15–16**: ClCOCH_2_CH_2_Cl, Na_2_CO_3_, THF, rt, 15 min.

For the synthesis of the target derivatives **17–37**, we used as starting material 2-methoxybezoic acid (**1**) or 3-methoxy-2-naphthoic acid (**2**), which were converted to the corresponding anhydrides by treatment with ethyl chloroformate (Scheme 1). Subsequent reaction with 2-amino-3-nitrobenzoic acid in the presence of Na_2_CO_3_ provided acids **3** and **4, respectively**. The next step concerns the synthesis of the benzoxazinone analogs **5** and **6** upon heating in acetic acid anhydride, followed by reaction with ammonia to yield the intermediate amides **7** and **8** [22,23]. Ring closure of the amides **7** and **8**, upon treatment with 5% aq. NaOH solution under reflux, provided the nitro quinazolinones **9** and **10** [24]. The nitro group was then easily reduced by hydrogenation to afford the amino derivatives **11** and **12**, which were converted to the corresponding amides **13–16** by treatment with chloroacetyl chloride or 3-chloropropionyl chloride.

**Scheme 2:**
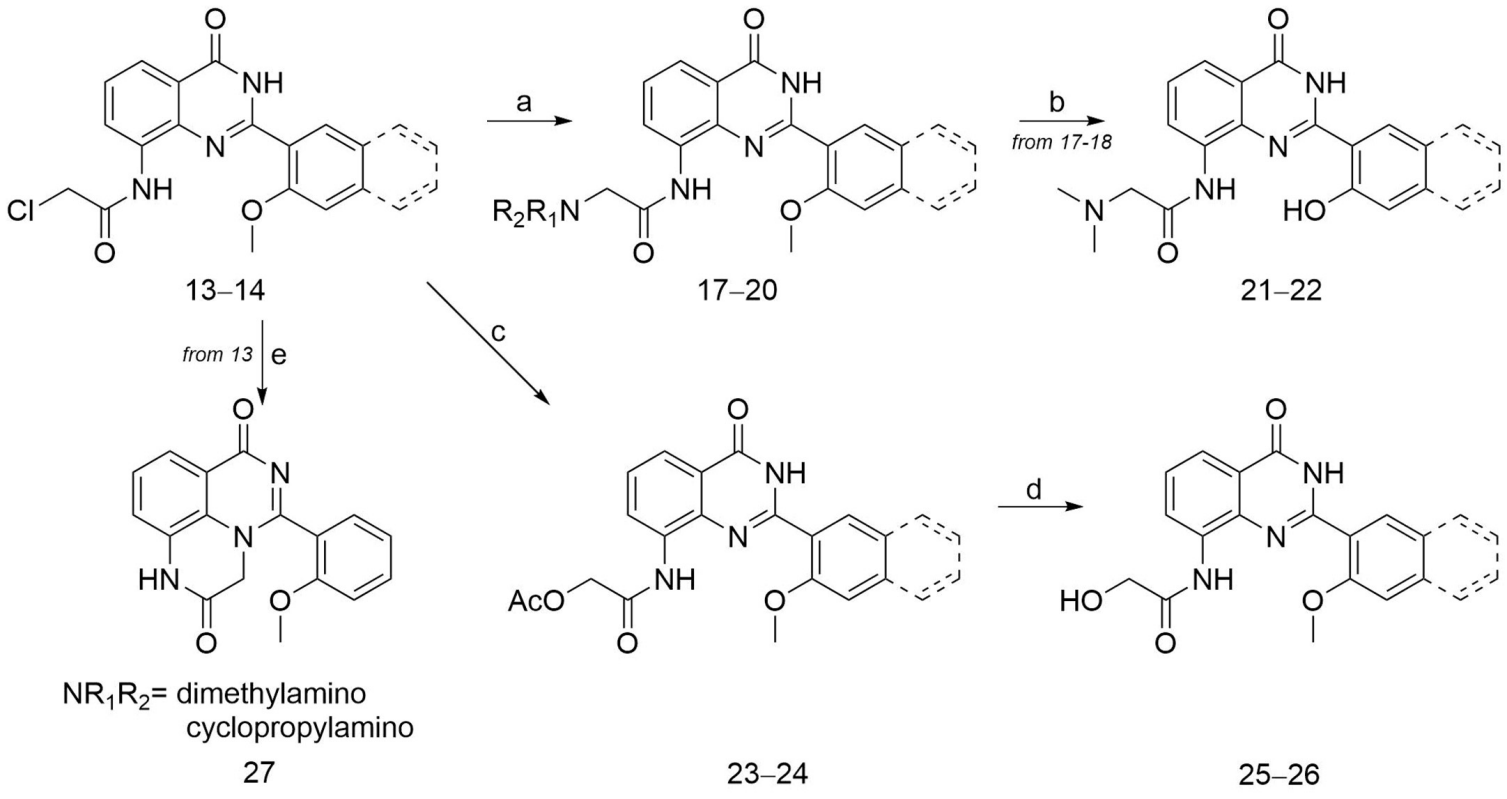
Reagents and conditions: (**a**) suitable amine, THF anh., autoclave, 100 °C, 65 h; (**b**) BBr_3_, CH_2_Cl_2_, −40 °C, 10 min, then 0 °C, 24 h; (**c**) DMF, CH_3_COOK, 50 °C, 2 h; (**d**) MeOH, 30% aq. NaOH sol., rt, 2 h; (**e**) MeOH, CH_3_COOK, rt, 18 h.

Reaction of these amides with the suitable amines resulted in the target amino-substituted compounds **17–20** (Scheme 2) [25]. The methoxy compounds **17** and **18** were then efficiently demethoxylated upon treatment with BBr_3_ to provide the phenol analogs **21** and **22**, respectively. For the preparation of hydroxy analogs **25** and **26**, we followed a slightly different approach. Specifically, chlorides **13** and **14** were first treated with potassium acetate in DMF to provide the corresponding acetates **23** and **24** that were saponified to provide the corresponding quinazolinones **25** and **26**, respectively. Interestingly, the reaction of chloride **13** with potassium acetate in methanol gave compound **27** upon intramolecular cyclisation.

**Scheme 3:**
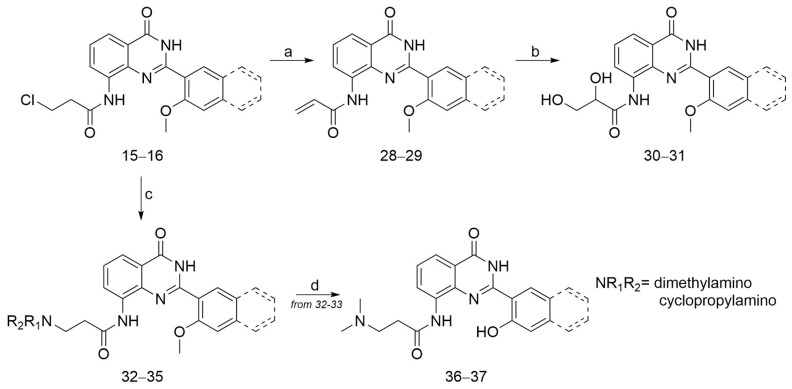
Reagents and conditions: (**a**) Et_3_N, THF anh., 70 °C, 18 h; (**b**) OsO_4_, *N*-methylmorpholine-*N*-oxide, THF anh., rt, 60 h; (**c**) suitable amine, THF anh., autoclave, 100 °C, 65 h; (**d**) BBr_3_, CH_2_Cl_2_, −40 °C, 10 min, then 0 °C, 24 h.

For the synthesis of the amides **32–35** and **36–37**, we followed a similar strategy. Therefore, chlorides **15** and **16** were converted into the corresponding amines **32–35** by treatment with suitable secondary amines (Scheme 3). 

Finally, the amides **32** and **33** underwent Lewis acid-mediated deprotection to provide the target amines **36** and **37**, respectively. Cis-diols **30** and **31** were prepared by catalytic syn-hydroxylation of the acrylamides **28** and **29**, with osmium tetroxide and *N*-methylmorpholine-*N*-oxide as the oxidizing agent [26]. 

### 2.2. Biological Assays

#### 2.2.1. Growth Inhibiting Activity 

All compounds were evaluated for their anti-proliferative activities against nine human cancer cell lines: LN-229 (glioblastoma), Capan-1 (pancreatic adenocarcinoma), Hap-1 (chronic myeloid leukemia), HCT-116 (colorectal carcinoma), NCI-H460 (lung carcinoma), DND-41 (acute lymphoblastic leukemia), HL-60 (acute myeloid leukemia), K-562 (chronic myeloid leukemia), and Z-138 (non-Hodgkin lymphoma). Staurosporine and Docetaxel were used as positive controls to validate the assay. As negative controls, the untreated cell lines were used, which allowed us to measure the baseline response in the absence of compound treatment. The results of the MTT dye reduction assay, expressed as 50% inhibitory concentrations (IC_50_) in µM, are depicted in Table 1.

Most of the new compounds exhibited no cytotoxic activity, with only five of them (specifically **17**, **21**, **25**, **32**, and **34**) demonstrating moderate inhibitory effects on cell growth in the low micromolar potency region. It is apparent that all five of these compounds are phenyl-substituted, indicating that the presence of the naphthyl group is unfavorable for the cytotoxic activity within this class of compounds. The results obtained suggest that the demethylation of the methoxy derivative **17**, resulting in the hydroxy analog **21**, significantly reduces antiproliferative activity in the tested cell lines. Similarly, the data imply that dimethylamino substitution enhances activity when compared to cyclopropylamino substitution. Conversely, increasing the side chain length from two (compounds **17–20**) to three carbons (compounds **32–35**) diminished antiproliferative activity.

The phenyl-substituted analog **17** was the most potent inhibitor within the cell lines tested, indicating that the methoxy phenyl substitution, along with a dimethylaminoacetamido side chain, clearly enhances cytotoxic activity. Upon direct comparison of their activity against all tested cell lines, it was evident that most of the compounds were more cytotoxic against the chronic myeloid leukemia cell line Hap-1.

#### 2.2.2. Kinases and Bromodomain Inhibition

All the synthesized compounds were screened against a panel of 113 proteins (109 kinases and 4 bromodomains), using Differential Scanning Fluorimetry (DSF) in order to assess their activity against these potential target molecules (Tables S1–S8, supporting information). The screening data highlighted 17 promising kinase targets with significant temperature shifts (Table 2). Compounds **17**, **21**, and **25** exhibited interesting profiles by binding to five kinases, with ΔT_m_ values comparable to the positive controls, Staurosporine, Silmitasertib, GW779439X, and GSK626616. Notably, compound **17** demonstrated the highest potency in the assay, stabilizing most of the highlighted kinases, with ΔT_m_ values half of those observed with the positive control.

DSF data measured on compound 17 demonstrated significant temperature stabilization of BMPK2, GSK3B, and MEK5 with ΔTm of 9 °C, 5.1 °C, and, 5.3 °C, respectively. ΔTm values were slightly lower for non-methoxylated compound 21, except for CK2A2 and PIM3 with ΔTm values at 3.9 °C and 6.5 °C, compared to 1.8 °C and 3.2 °C of compound **17**. Bulkier substituents in the side chain (compound **19**) resulted in a loss of potency to around half of that seen in compound **17**, except for DAPK3, MST3, and DYRK1A with ΔTm at 4.1 °C, 3 °C, and 5 °C, respectively. Compounds **32** and **34**, where the side chain was extended, demonstrated a loss of their potency, except for GSG2 with ΔTm values around 3 °C. Furthermore, this increase in the side chain’s length, combined with a phenolic substituent on the quinazoline ring (compound **36**), led to a complete loss of potency across the tested kinases. 

Naphthyl-substituted compounds either bearing a 2-methoxy or a 2-hydroxy substitution were not potent against all targets, except for compounds **18** and **20** which retained their ΔT_m_ values against GSK3b at around 4 °C, comparable to compound **17**. The addition of a polar side chain favored the stabilization of MEK5 and DYRK2, with compounds **25** and **30** showing interesting ΔT_m_ values at 6.5 and 6.1 °C for MEK5 and 3.8 °C and 3.7 °C for DYRK2, respectively. Furthermore, these compounds exhibited a selectivity profile favoring MEK5 over the other tested MEKs, namely MEK1 and MAP2K7 (see Supplementary Material). This provides valuable insights for further investigation.

## 3. Materials and Methods 

### 3.1. General Information

All commercially available reagents and solvents were purchased from Alfa Aesar (Ward Hill, MA, USA) and used without any further purification. Melting points were determined on Büchi apparatus and were uncorrected. One-dimensional (^1^H NMR, ^13^C NMR) and two-dimensional (COSY, NOESY, HMBC, HSQC-DEPT135) spectra were carried out on a Bruker Avance III-600 MHz spectrometer (Karlsruhe, Germany). Chemical shifts (δ) are expressed in ppm while coupling constants (*J*) are in Hz. The multiplicity of vertices is expressed as s (singlet), d (doublet), t (triplet), q (quartet), dd (doublet of doublets), and m (multiple). ^1^H-NMR and ^13^C-NMR are available online in the supplementary material. Flash chromatography was performed on Merck silica gel (40–63 μm) with the indicated solvent system using gradients of increasing polarity in most cases (Merck KGaA—Darmstadt, Germany). The reactions were monitored by analytical thin-layer chromatography (Merck pre-coated silica gel 60 F254 TLC plates, 0.25 mm layer thickness). Mass spectra were recorded on a UPLC Triple TOF-MS (UPLC: Acquity of Waters (Milford, MA 01757, USA), SCIEX Triple TOF-MS 5600+ (Framingham, MA 01701, USA)).

### 3.2. Synthesis of Compounds **3–37**

2-(2-Methoxybenzamido)-3-nitrobenzoic acid (**3**). To a solution of 2-methoxybenzoic acid (**1**) (150 mg, 1.08 mmol) in ACN (20 mL) at 0 °C, Et_3_N (275 μL, 1.97 mmol) and ClCO_2_Et (0.1 mL, 1.08 mmol) were added under argon. The resulting mixture was stirred for 30 min at room temperature; after which, Na_2_CO_3_ (195 mg, 2.96 mmol) and 2-amino-3-nitrobenzoic acid (200 mg, 1.08 mmol) were added, and the mixture was heated at 50 °C for 24 h. The reaction mixture was then vacuum evaporated, diluted with water, and acidified with 9% aq. HCl solution (pH ≈ 3). The precipitate was filtered and air-dried to give crude **3**, which was purified by column chromatography (silica gel) using a mixture of CH_2_Cl_2_/CH_3_OH 100/2–100/16 as the eluent to afford 180 mg (57%) of the title compound as a yellow solid. Mp.: 134–136 °C (EtOAc). ^1^H NMR (600 MHz, DMSO-*d*_6_) δ (ppm) 8.24 (dd, *J* = 7.8, 1.6 Hz, 1H, H-6), 8.11 (dd, *J* = 8.1, 1.6 Hz, 1H, H-4), 7.96 (dd, *J* = 7.8, 1.9 Hz, 1H, H-6′), 7.61 (td, *J* = 8.4, 1.8 Hz, 1H, H-4′), 7.45 (t, *J* = 8.0 Hz, 1H, H-5), 7.26 (dd, *J* = 8.4, 1.0 Hz, 1H, H-3′), 7.11 (dd, *J* = 8.1, 1.0 Hz, 1H, H-5′), 4.05 (s, 3H, OC*H*_3_).^13^C NMR (151 MHz, DMSO-*d*_6_) δ (ppm) 167.3 (NH*C*O), 163.1 (*C*OOH), 157.8 (C-2′), 144.8 (C-3), 134.8 (C-6), 134.4 (C-4′), 131.7 (C-6′), 130.8 (C-2), 128.1 (C-4), 126.5, (C-1), 124.4 (C-5), 120.9, (C-5′), 120.1, (C-1′), 112.5 (C-3′), 56.1 (O*C*H_3_).

2-(3-Methoxy-2-naphthamido)-3-nitrobenzoic acid (**4**). This compound was synthesized by an analogous procedure as described for the preparation of compound **3**, using 3-methoxy-2-naphthoic acid (**2**). Yield: 55%. Mp.: 145–147 °C (EtOAc). ^1^H NMR (600 MHz, DMSO-*d*_6_) δ (ppm) 12.03 (brs, 1H, D_2_O exch., N*H*), 8.62 (s, 1H, H-1′), 8.26 (dd, *J* = 7.8, 1.6 Hz, 1H, H-6), 8.19 (dd, *J* = 8.2, 1.6 Hz, 1H, H-4), 8.05 (d, *J* = 8.2 Hz, 1H, H-8′), 7.92 (d, *J* = 8.2 Hz, 1H, H-5′), 7.64–7.58 (m, 2H, H-4′, H-6′), 7.52 (t, *J* = 8.0 Hz, 1H, H-5), 7.45 (dd, *J* = 8.1, 6.8, 1.2 Hz, 1H, H-7′), 4.17 (s, 3H, OC*H*_3_). ^13^C NMR (151 MHz, DMSO-*d*_6_) δ (ppm) 167.1 (NH*C*O), 163.0 (*C*OOH), 154.4 (C-3′), 144.9 (C-3), 136.0 (C-4a’), 134.9 (C-6), 133.6 (C-1′), 130.6 (C-2), 129.1 (C-8′), 128.9 (C-6′), 128.6 (C-4), 127.5 (C-8a’), 126.4 (C-5′), 125.5 (C-1), 124.9 (C-5), 124.7 (C-7′), 121.3 (C-2′), 107.3 (C-4′), 56.1 (O*C*H_3_).

2-(2-Methoxyphenyl)-8-nitroquinazolin-4(3*H*)-one (**9**). A suspension of compound **3** (1 g, 3.16 mmol) in (CH_3_CO)_2_O (6.83 mL, 72.28 mmol) was refluxed for 2 h. The volatiles were then vacuum evaporated, and the residue was treated with NH_3_ (0.5 M solution in THF, 15 mL). After completion of the reaction, the solvent was vacuum evaporated, and the residue was dissolved in 5% aq. NaOH solution (10 mL) and refluxed for 10 min. After cooling, the reaction mixture was diluted with water and acidified with 9% aq. HCl solution (pH ≈ 3). The precipitate was filtered, washed with water, and air-dried to give compound **9** (750 mg, 79.8%), practically pure, which was used for the next step without any further purification. Mp.: 168–170 °C (EtOAc). ^1^H NMR (400 MHz, DMSO-*d*_6_) δ (ppm) 8.37 (dd, *J* = 8.0, 1.5 Hz, 1H, H-5), 8.30 (dd, *J* = 7.8, 1.5 Hz, 1H, H-7), 7.66 (m, 2H, H-6, H-6′), 7.57 (td, *J* = 8.8, 1.8 Hz, 1H, H-4′), 7.22 (d, *J* = 8.4 Hz, 1H, H-3′), 7.11 (t, *J* = 7.5 Hz, 1H, H-5′), 3.88 (s, 3H, OC*H*_3_).^13^C NMR (151 MHz, DMSO-*d*_6_) δ (ppm) 159.9 (*C*O), 157.4 (C-2′), 154.8 (C-2), 146.8 (C-8), 140.8 (C-8a), 133.1 (C-4′), 130.7 (C-6′), 129.7 (C-5), 128.1, (C-7), 126.2 (C-6), 122.5, (C-4a), 121.8, (C-1′), 120.7 (C-5′), 112.2 (C-3′), 56.0 (O*C*H_3_).

2-(3-Methoxynaphthalen-2-yl)-8-nitroquinazolin-4(3*H*)-one (**10**). This compound was synthesized by an analogous procedure as described for the preparation of compound **9**. Yield: 80%. Mp.: 186–188 °C (EtOAc-*n*-Pentane). ^1^H NMR (400 MHz, DMSO-*d*_6_) δ (ppm) 8.41 (d, *J* = 8.0 Hz, 1H, H-5), 8.33 (d, *J* = 7.8 Hz, 1H, H-7), 8.16 (s, 1H, H-1′), 7.98 (d, *J* = 6.25 Hz, 1H, H-8′), 7.91 (d, *J* = 6.24 Hz, 1H, H-5′), 7.70 (t, *J* = 7.9 Hz, 1H, H-6), 7.58 (t, *J* = 7.9 Hz, 1H, H-6′), 7.54 (s, 1H, H-4′), 7.43 (td, *J* = 8.0, 1.0 Hz, 1H, H-7′), 3.95 (s, 3H, OC*H*_3_). ^13^C NMR (101 MHz, DMSO-*d*_6_) δ (ppm) 160.0 (*C*O), 154.9 (C-3′), 154.4 (C-2), 146.8 (C-8), 140.7 (C-8a), 135.4 (C-4a’), 131.0 (C-1), 129.7 (C-5), 128.5 (C-6′), 128.2 (C-8′), 128.1 (C-7), 127.4 (C-8a’), 126.7 (C-5′), 126.4 (C-6), 124.6 (C-7′), 124.2 (C-2′), 122.6 (C-4a), 106.7 (C-4′), 56.0 (O*C*H_3_).

2-Chloro-*N*-(2-(2-methoxyphenyl)-4-oxo-3,4-dihydroquinazolin-8-yl)acetamide (**13**). A solution of **9** (200 mg, 0.67 mmol) in abs. EtOH (10 mL) was hydrogenated in the presence of Pd/C (15 mg), under pressure (50 psi), at room temperature, for 4 h. After completion of the reaction, the mixture was filtered through a Celite pad, and the filtrate was evaporated to dryness to provide the amino derivative **11**. Without further purification, the oily residue was dissolved in THF (10 mL) and CH_2_Cl_2_ (5 mL). To this solution, Na_2_CO_3_ (210 mg, 2.01 mmol) and chloroacetyl chloride (59 μL, 0.74 mmol) were added. The resulting suspension was stirred for 15 min at room temperature. The volatiles were then vacuum evaporated, and the residue was diluted with water and acidified with 9% aq. HCl solution (pH ≈ 3). The precipitate was filtered, washed with water, and air dried to afford the title compound **13** (161 mg, 70%), practically pure, which was used for the next step without any further purification. Mp.: 132–134 °C (MeOH). ^1^H NMR (600 MHz, DMSO-*d*_6_) δ (ppm) 12.20 (brs, 1H, D_2_O exch., N*H*), 10.20 (brs, 1H, D_2_O exch., N*H*CO), 8.62 (dd, *J* = 8.0, 1.4 Hz, 1H, H-7), 7.90 (dd, *J* = 7.6, 1.8 Hz, 1H, H-6′), 7.86 (dd, *J* = 8.0, 1.4 Hz, 1H, H-5), 7.57 (td, *J* = 8.8, 1.8 Hz, 1H, H-4′), 7.52 (t, *J* = 8.0 Hz, 1H, H-6), 7.23 (d, *J* = 8.3 Hz, 1H, H-3′), 7.14 (t, *J* = 7.5 Hz, 1H, H-5′), 4.54 (s, 2H, C*H*_2_), 3.90 (s, 3H, OC*H*_3_).^13^C NMR (151 MHz, DMSO-*d*_6_) δ (ppm) 164.9 (NH*C*O), 160.9 (*C*O), 157.4 (C-2′), 152.0 (C-2), 138.9 (C-8a), 133.2 (C-4a), 132.8 (C-4′), 130.9 (C-6′), 126.7 (C-6), 122.4 (C-7), 121.9 (C-5′), 120.9 (C-8), 120.7 (C-5), 120.2 (C-1′), 112.1 (C-3′), 56.0 (O*C*H_3_), 43.6 (*C*H_2_).

2-Chloro-*N-*(2-(3-methoxynaphthalen-2-yl)-4-oxo-3,4-dihydroquinazolin-8-yl)acetamide (**14**). This compound was synthesized by an analogous procedure as described for the preparation of compound **13**. Yield: 77%. Mp.: 156–158 °C (THF-*n*-Pentane). ^1^H NMR (600 MHz, DMSO-*d*_6_) δ (ppm) 12.45 (brs, 1H, D_2_O exch., N*H*), 10.24 (brs, 1H, D_2_O exch., N*H*CO), 8.67 (dd, *J* = 8.0, 1.4 Hz, 1H, H-7), 8.41 (s, 1H, H-1′), 7.93 (d, *J* = 8.1 Hz, 1H, H-8′), 7.89 (d, *J* = 8.2 Hz, 1H, H-5′), 7.59 (dd, *J* = 8.2, 1.3 Hz, 1H,H-6′), 7.57–7.52 (m, 2H, H-6, H-4′), 7.45 (dd, *J* = 8.0, 1.2 Hz, 1H, H-7′), 4.55 (s, 2H, C*H*_2_), 3.98 (s, 3H, OC*H*_3_). ^13^C NMR (151 MHz, DMSO-*d*_6_) δ (ppm) 164.8 (NH*C*O), 160.9 (*C*O), 154.6 (C-3′), 151.8 (C-2), 138.8 (C-8a), 135.3 (C-4a’), 133.3 (C-4a), 131.3 (C-1′), 128.4 (C-6′), 128.0 (C-8′), 127.6 (C-8a’), 126.7 (C-6), 126.6 (C-5′, C-7′), 124.5 (C-2′), 122.4 (C-7), 121.0 (C-8), 120.3 (C-2′), 106.6 (C-4′), 56.9 (O*C*H_3_), 43.6 (*C*H_2_).

3-Chloro-*N*-(2-(2-methoxyphenyl)-4-oxo-3,4-dihydroquinazolin-8-yl)propanamide (**15**). This compound was synthesized by an analogous procedure as described for the preparation of compound **13**. Yield: 67%. Mp.: 153–155 °C (MeOH). ^1^H NMR (400 MHz, DMSO-*d*_6_) δ (ppm) 12.18 (brs, 1H, D_2_O exch., N*H*), 9.76 (brs, 1H, D_2_O exch., N*H*CO), 8.63 (dd, *J* = 8.3, 1.6 Hz, 1H, H-7), 7.96 (dd, *J* = 7.6, 1.8 Hz, 1H, H-6′), 7.83 (dd, *J* = 8.0, 1.4 Hz, 1H, H-5), 7.57 (td, *J* = 8.5, 1.8 Hz, 1H, H-4′), 7.49 (t, *J* = 8.0 Hz, 1H, H-6), 7.22 (dd, *J* = 8.6, 1.0 Hz, 1H, H-3′), 7.13 (td, *J* = 7.5, 1.0 Hz, 1H, H-5′), 3.94–3.85 (m, 5H, OC*H*_3_, COCH_2_C*H*_2_), 3.06 (t, *J* = 6.3 Hz, 2H, COC*H*_2_CH_2_).^13^C NMR (101 MHz, DMSO-*d*_6_) δ (ppm) 168.5 (NH*C*O), 160.9 (*C*O), 157.4 (C-2′), 151.6 (C-2), 138.9 (C-8a), 134.0 (C-4a), 132.6 (C-4′), 131.2 (C-6′), 126.5 (C-6), 123.4 (C-7), 122.1 (C-5′), 120.9 (C-8), 120.6 (C-5), 119.9 (C-1′), 111.9 (C-3′), 55.9 (O*C*H_3_), 40.7 (COCH_2_*C*H_2_), 30.7 (CO*C*H_2_CH_2_).

3-Chloro-*N*-(2-(3-methoxynaphthalen-2-yl)-4-oxo-3,4-dihydroquinazolin-8-yl)propanamide (**16**). This compound was synthesized by an analogous procedure as described for the preparation of compound **13**. Yield: 81%. Mp.: 167–169 °C (THF-*n*-Pentane). ^1^H NMR (600 MHz CDCl_3_) δ (ppm) 10.96 (brs, 1H, D_2_O exch., N*H*), 9.58 (s brs, 1H, D_2_O exch., N*H*CO), 8.95 (s, 1H, H-1′), 8.87 (dd, *J* = 7.9, 1.4 Hz, H-7), 8.00 (dd, *J* = 8.0, 1.4 Hz, H-5), 7.96 (d, *J* = 8.1 Hz, H-8′), 7.81 (d, *J* = 8.2 Hz, 1H, H-5′), 7.59 (dd, *J* = 8.1, 1.2 Hz, 1H, H-6′), 7.51–7.44 (m, 2H, H-6, H-7′), 7.35 (s, 1H, H-4′), 4.16 (s, 3H, OC*H*_3_), 4.01 (t, *J* = 6.3 Hz, 2H, COCH_2_C*H*_2_), 3.06 (t, *J* = 6.3 Hz, 2H, COC*H*_2_CH_2_). ^13^C NMR (151 MHz, CDCl_3_) δ (ppm) 168.0 (NH*C*O), 161.4 (*C*O), 154.8 (C-3′), 150.3 (C-2), 138.6 (C-8a), 136.0 (C-4a’), 133.9 (C-4a), 133.1 (C-1′), 129.2 (C-6′), 129.1 (C-8′), 128.5 (C-8a’), 127.3 (C-6), 126.7 (C-5′), 125.4 (C-7′), 122.9 (C-7), 120.9 (C-5), 120.7 (C-8), 120.6 (C-2′), 107.5 (C-4′), 56.4 (O*C*H_3_), 41.3 (COCH_2_*C*H_2_), 40.2 (CO*C*H_2_CH_2_).

2-(Dimethylamino)-*N*-(2-(2-methoxyphenyl)-4-oxo-3,4-dihydroquinazolin-8-yl)acetamide (**17**). To a solution of chloride **13** (80 mg, 0.23 mmol) in anh. THF (10 mL), a 5.6 M ethanolic solution of dimethylamine (4.66 mmol) was added dropwise and the resulting mixture was heated at 100 °C, in an autoclave apparatus, for 65 h. After cooling, the solvent was vacuum evaporated and the oily residue was purified by column chromatography (silica gel, CH_2_Cl_2_/MeOH 95/5) to afford **17** (59 mg, 73.2%). Mp.: >230 °C (EtOAc-*n*-Pentane). IR (Nujol) ν max/cm^−1^: 1671.98 (CO). ^1^H NMR (600 MHz, CDCl_3_) δ (ppm) 11.19 (brs, 1H, D_2_O exch., N*H*), 11.10 (brs, 1H, D_2_O exch., N*H*CO), 8.78 (dd, *J* = 7.9, 1.4 Hz, 1H, H-7), 8.62 (dd, *J* = 8.0, 1.8 Hz, 1H, H-6′), 7.92 (dd, *J* = 8.0, 1.4 Hz, 1H, H-5), 7.53 (td, *J* = 8.4, 1.8 Hz, 1H, H-4′), 7.41 (t, *J* = 8.0 Hz, 1H, H-6), 7.14 (td, *J* = 8.1, 1.0 Hz, 1H, H-5′), 7.09 (dd, *J* = 8.4, 1.0 Hz, 1H, H-3′), 4.08 (s, 3H, OC*H*_3_), 3.22 (s, 2H, C*H*_2_), 2.52 (s, 6H, (C*H*_3_)_2_). ^13^C NMR (151 MHz, CDCl_3_) δ (ppm) 169.1 (NH*C*O), 161.7 (*C*O), 158.4 (C-2′), 149.7 (C-2), 138.9 (C-8a), 134.0 (C-4a), 133.6 (C-4′), 131.2 (C-6′), 127.0 (C-6), 122.3 (C-7), 121.8 (C-5′), 120.9 (C-8), 120.2 (C-5), 119.5 (C-1′), 112.3 (C-3′), 64.1 (*C*H_2_), 56.4 (O*C*H_3_), 46.3 (*C*H_3_)_2_).

2-(Dimethylamino)-*N*-(2-(3-methoxynaphthalen-2-yl)-4-oxo-3,4-dihydroquinazolin-8-yl)acetamide (**18**). This compound was synthesized by an analogous procedure as described for the preparation of compound **17**. Yield: 57%. Mp.: >230 °C (THF-*n*-Pentane). IR (Nujol) ν max/cm^−1^: 1668.23 (CO). ^1^H NMR (600 MHz, CDCl_3_) δ (ppm) 11.19 (brs, 2H, D_2_O exch., N*H*, N*H*CO), 9.16 (s, 1H, H-1′), 8.89 (dd, *J* = 7.9, 1.4 Hz, 1H, H-7), 7.98 (dd, *J* = 8.0, 1.4 Hz, 1H, H-5), 7.89 (d, *J* = 8.1 Hz, 1H, H-8′), 7.82 (d, *J* = 8.2 Hz, 1H, H-5′), 7.59 (td, *J* = 8.1, 1.2 Hz, 1H, H-6′), 7.48 (m, 2H, H-6, H-7′), 7.37 (s, 1H, H-4′), 4.19 (s, 3H, OC*H*_3_), 3.27 (s, 2H, C*H*_2_), 2.61 (s, 6H, (C*H*_3_)_2_).^13^C NMR (151 MHz, CDCl_3_) δ (ppm) 169.2 (NH*C*O), 161.7 (*C*O), 155.3 (C-3′), 149.8 (C-2), 139.0 (C-8a), 136.1 (C-4a’), 134.2 (C-4a), 133.0 (C-1′), 129.0 (C-6′), 128.8 (C-8′), 128.8 (C-8a’), 127.3 (C-6), 126.9 (C-5′), 125.5 (C-7′), 122.6 (C-7), 121.1 (C-8), 120.7 (C-2′), 120.4 (C-5), 107.6 (C-4′), 64.4 (*C*H_2_), 56.5 (O*C*H_3_), 46.6 ((*C*H_3_)_2_).

2-(Cyclopropylamino)-*N*-(2-(2-methoxyphenyl)-4-oxo-3,4-dihydroquinazolin-8-yl)acetamide (**19**): This compound was synthesized by an analogous procedure as described for the preparation of compound **17**. Yield: 63%. Mp.: >230 °C (THF-*n*-Pentane). IR (Nujol) ν max/cm^−1^: 1669.11 (CO). ^1^H NMR (600 MHz, CDCl_3_) δ (ppm) 11.07 (brs, 1H, D_2_O exch., N*H*), 10.91 (brs, 1H, D_2_O exch., N*H*CO), 8.88 (dd, *J* = 7.8, 1.3 Hz, 1H, H-7), 8.57 (dd, *J* = 8.0, 1.8 Hz, 1H, H-6′), 7.93 (dd, *J* = 7.8, 1.3 Hz, 1H, H-5), 7.55 (dd, *J* = 7.9, 1.8 Hz, 1H, H-4′), 7.41 (t, *J* = 7.9 Hz, 1H, H-6), 7.19 (t, *J* = 7.6 Hz, 1H, H-5′), 7.10 (d, *J* = 8.5 Hz, 1H, H-3′), 4.08 (s, 3H, OC*H*_3_), 3.64 (s, 2H, C*H*_2_), 2.37 (m, 1H, C*H*-cyclopropyl), 0.63–0.52 (m, 4H, C*H*_2_-cyclopropyl). ^13^C NMR (151 MHz, CDCl_3_) δ (ppm) 170.6 (NH*C*O), 161.7 (*C*O), 158.3 (C-2′), 149.8 (C-2), 138.8 (C-8a), 134.1 (C-4a), 133.7 (C-4′), 131.4 (C-6′), 127.1 (C-6), 122.3 (C-7), 121.8 (C-5′), 120.9 (C-8), 120.3 (C-5), 119.5 (C-1′), 112.3 (C-3′), 56.4 (O*C*H_3_), 54.1 (*C*H_2_), 31.7 (*C*H-cyclopropyl), 6.8 (*C*H_2_-cyclopropyl); HRMS (ESI^+^) *m*/*z* 365.1617 (calcd for C_20_H_21_N_4_O_3_^+^, 365.1608).

2-(Cyclopropylamino)-*N*-(2-(3-methoxynaphthalen-2-yl)-4-oxo-3,4-dihydroquinazolin-8-yl)acetamide (**20**). This compound was synthesized by an analogous procedure as described for the preparation of compound **17**. Yield: 88%. Mp.: >230 °C (THF-*n*-Pentane). IR (Nujol) ν max/cm^−1^: 1667.75 (CO). ^1^H NMR (600 MHz, CDCl_3_) δ (ppm) 10.99 (brs, 1H, D_2_O exch., N*H*), 10.95 (brs, 1H, D_2_O exch., N*H*CO), 9.01 (s, 1H, H-1′), 8.93 (dd, *J* = 8.0, 1.4 Hz, 1H, H-7), 7.99–7.94 (m, 2H, H-5, H-8′), 7.81 (d, *J* = 8.2 Hz, 1H, H-5′), 7.58 (dd, *J* = 8.1, 1.2 Hz, 1H, H-6′), 7.50–7.42 (m, 2H, H-6, H-7′), 7.32 (s, 1H, H-4′), 4.14 (s, 3H, OC*H*_3_), 3.66 (s, 2H, C*H*_2_), 2.35 (m, 1H, C*H*-cyclopropyl), 0.63–0.57 (m, 2H, C*H*_2_-cyclopropyl), 0.63–0.57 (m, 2H, C*H*_2_-cyclopropyl). ^13^C NMR (151 MHz, CDCl_3_) δ (ppm) 170.8 (NH*C*O), 161.6 (*C*O), 155.0 (C-3′), 149.9 (C-2), 138.9 (C-8a), 136.0 (C-4a’), 134.2 (C-4a), 132.9 (C-1′), 129.1 (C-6′), 128.9 (C-8′), 128.6 (C-8a’), 127.3 (C-6), 126.8 (C-5′), 125.2 (C-7′), 122.5 (C-7), 121.1 (C-8), 120.9 (C-2′), 120.3 (C-5), 107.5 (C-4′), 56.4 (O*C*H_3_), 54.2 (*C*H_2_), 32.1 (*C*H-cyclopropyl), 7.0 (*C*H_2_-cyclopropyl); HRMS (ESI^+^) *m*/*z* 415.1770 (calcd for C_24_H_23_N_4_O_3_^+^, 415.1765).

2-(Dimethylamino)-*N*-(2-(2-hydroxyphenyl)-4-oxo-3,4-dihydroquinazolin-8-yl)acetamide (**21**). BBr_3_ (0.52 mL, 0.51 mmol, 10% solution in CH_2_Cl_2_) was added dropwise to a solution of **17** (30 mg, 0.085 mmol) in anh. CH_2_Cl_2_ (5 mL), at −40 °C, under argon, and the reaction mixture was stirred at this temperature for 10 min and at 0 °C for 24 h. Afterwards, MeOH (10 mL) and a saturated NaHCO_3_ solution were added (0.5 mL) and stirring was continued for 10 min. Most of the organic solvents were vacuum evaporated. The residue was then extracted with EtOAc, the organic phase was washed with a saturated NaHCO_3_ solution, water, and brine, dried (anh. Na_2_SO_4_), and concentrated to dryness. The crude product was purified by column chromatography (silica gel), using a mixture of CH_2_Cl_2_/CH_3_OH 100/2 to 100/8 as the eluent, to afford 13 mg (45%) of the title compound. Mp.: >230 °C (EtOAc). IR (Nujol) ν max/cm^−1^: 1668.51 (CO). ^1^H NMR (600 MHz, CDCl_3_-MeOD) δ (ppm) 8.80 (dd, *J* = 7.9, 1.4 Hz, 1H, H-7), 8.45 (dd, *J* = 8.3, 1.7 Hz, 1H, H-6′), 8.01 (dd, *J* = 8.0, 1.4 Hz, 1H, H-5), 7.55–7.50 (m, 2H, H-6, H-4′), 7.13–7.11 (m, 2H, H-3′, H-5′), 3.31 (s, 2H, C*H*_2_), 2.62 (s, 6H, (C*H*_3_)_2_). ^13^C NMR (151 MHz, CDCl_3_-MeOD) δ (ppm) δ 170.7 (NH*C*O), 163.0 (*C*O), 158.4 (C-2′), 151.9 (C-2), 139.3 (C-8a), 134.2 (C-4′), 133.5 (C-4a), 129.6 (H-6′), 127.2 (C-6), 123.5 (C-7), 121.0 (C-5′, C-8), 120.6 (C-5), 118.0 (C-3′), 116.4 (C-1′), 64.1 (*C*H_2_), 46.3 (*C*H_3_)_2_); HRMS (ESI^+^) *m*/*z* 339.1457 (calcd for C_18_H_19_N_4_O_3_^+^, 339.1452).

2-(Dimethylamino)-*N*-(2-(3-hydroxynaphthalen-2-yl)-4-oxo-3,4-dihydroquinazolin-8-yl)acetamide (**22**). This compound was synthesized by an analogous procedure as described for the preparation of compound **21**. Yield: 40%. Mp.: >230 °C (EtOAc). IR (Nujol) ν max/cm^−1^: 1670.07 (CO). ^1^H NMR (600 MHz, DMSO-*d*_6_) δ (ppm) 10.84 (brs, 2H, D_2_O exch., N*H*CO, N*H*), 8.81 (s, 1H, H-1′), 8.61 (d, *J* = 8.4 Hz, 1H, H-7), 7.85–7.88 (m, 2H, H-5′, H-8′), 7.79 (d, *J* = 8.0 Hz, 1H, H-5), 7.55–7.47 (m, 2H, H-6′, H-7′), 7.43–7.35 (m, 2H, H-6, H-4′), 3.30 (m, 2H, C*H*_2_), 2.55 (s, 6H, (C*H*_3_)_2_). ^13^C NMR (151 MHz, DMSO-*d*_6_) δ (ppm) 167.8 (NH*C*O), 161.1 (*C*O), 154.0 (C-3′), 152.1 (C-2), 138.7 (C-8a), 135.8 (C-4a’), 133.2 (C-4a), 130.7 (C-1′), 128.4 (C-6′, C-8a’), 128.2 (C-8′), 127.0 (C-6), 126.5 (C-5′), 125.9 (C-7′), 124.0 (C-7), 121.1 (C-8), 120.9 (C-2′), 120.0 (C-5), 119.9 (C-2′), 110.9 (C-4′), 62.4 (*C*H_2_), 45.4 ((*C*H_3_)_2_); HRMS (ESI^+^) *m*/*z* 389.1615 (calcd for C_22_H_21_N_4_O_3_^+^, 389.1608).

2-Hydroxy-*N*-(2-(2-methoxyphenyl)-4-oxo-3,4-dihydroquinazolin-8-yl)acetamide (**25**). To a solution of **13** (100 mg, 0.29 mmol) in anh. DMF (20 mL), under argon, was added CH_3_COOK (57 mg, 0.58 mmol) and the mixture was heated at 50 °C for 2 h. After completion of the reaction, the mixture was poured into water and acidified with 9% aq. HCl solution (pH ≈ 3). The precipitate was filtered, washed with water, and air dried to afford crude intermediate **23** (65 mg, 0.18 mmol), which was purified by column chromatography (silica gel) using a mixture of CH_2_Cl_2_/EtOAc 100/10 to CH_2_Cl_2_/EtOAc 25/10 as the eluent. This compound was then dissolved in MeOH (10 mL) and treated with 30% aq. NaOH solution at room temperature for 2 h. The resulting precipitate was filtered and purified by column chromatography (silica gel), using a mixture of CH_2_Cl_2_/CH_3_OH 100/4 to CH_2_Cl_2_/CH_3_OH 100/12 as the eluent, to afford 30 mg (52.6%) of the title compound **25**. Mp.: 113–115 °C (THF-*n*-Pentane). IR (Nujol) ν max/cm^−1^: 1668.11 (CO). ^1^H NMR (600 MHz, DMSO-*d*_6_) δ (ppm) 10.53 (brs, 1H, D_2_O exch., N*H*CO), 8.64 (dd, *J* = 7.8, 1.4 Hz, 1H, H-7), 7.74 (dd, *J* = 8.0, 1.5 Hz, 1H, H-6′), 7.68 (dd, *J* = 7.6, 1.8 Hz, 1H, H-5), 7.45 (dd, *J* = 8.9, 1.8 Hz, 1H, H-4′), 7.31 (t, *J* = 7.9 Hz, 1H, H-6), 7.14 (d, *J* = 8.3 Hz, 1H, H-3′), 7.05 (td, *J* = 7.4, 1.0 Hz, 1H, H-5′), 4.03 (s, 2H, C*H*_2_), 3.83 (s, 3H, OC*H*_3_). ^13^C NMR (151 MHz, DMSO-*d*_6_) δ (ppm) 174.3 (NH*C*O), 170.6 (*C*O), 157.3 (C-2′, C-2), 140.0 (C-8a), 132.7 (C-4a), 132.8 (C-4′), 130.5 (C-6′), 127.8 (C-6), 124.3 (C-8), 120.7 (C-5′), 120.2 (C-7), 119.5 (C-5), 119.0 (C-1′), 112.2 (C-3′), 61. 9 (*C*H_2_), 55.9 (O*C*H_3_); HRMS (ESI^+^) *m*/*z* 326.1141 (calcd for C_17_H_16_N_3_O_4_^+^, 326.1135).

2-Hydroxy-*N*-(2-(3-methoxynaphthalen-2-yl)-4-oxo-3,4-dihydroquinazolin-8-yl)acetamide (**26**). This compound was synthesized by an analogous procedure as described for the preparation of compound **25**. Yield: 58%. Mp.: 198–200 °C (THF-*n*-Pentane). IR (Nujol) ν max/cm^−1^: 1669.74 (CO). ^1^H NMR (600 MHz, DMSO-*d*_6_) δ (ppm) 10.53 (brs, 1H, D_2_O exch., N*H*CO), 8.80 (dd, *J* = 8.0, 1.4 Hz, 1H, H-7), 8.32 (s, 1H, H-1′), 7.96 (d, *J* = 8.1 Hz, 1H, H-8′), 7.93 (d, *J* = 8.2 Hz, 1H, H-5′), 7.81 (dd, *J* = 8.2, 1.42Hz, 1H, H-5), 7.59 (dd, *J* = 8.2, 1.2 Hz, 1H, H-6′), 7.55 (s, 1H, H-4′), 7.52 (t, *J* = 8.0 Hz, 1H, H-6), 7.45 (td, *J* = 8.0, 6.8, 1.2 Hz, 1H, H-7′), 4.06 (s, 2H, C*H*_2_), 3.98 (s, 3H, OC*H*_3_). ^13^C NMR (151 MHz, DMSO-*d*_6_) δ (ppm) 171.0 (NH*C*O), 164.4 (*C*O), 155.7 (C-3′, C-2), 140.6 (C-8a), 135.0 (C-4a’), 133.1 (C-4a), 130.6 (C-1′), 128.5 (C-8′), 128.2 (C-8a’), 127.6 (C-6′, C-6), 126.9 (C-5′), 124.5 (C-7′), 121.4 (C-8), 120.1 (C-5, C-7), 119.4 (C-2′), 106.7 (C-4′), 62.4 (*C*H_2_). 56.2 (O*C*H_3_); HRMS (ESI^+^) *m*/*z* 376.1287 (calcd for C_21_H_18_N_3_O_4_^+^, 376.1292).

5-(2-Methoxyphenyl)-3-hydro-1*H*,7*H*-pyrazino[3,2,1-*ij*]quinazoline-2,7-dione (**27**). A suspension of **13** (40 mg, 0.11 mmol) and CH_3_COOK (23 mg, 0.23 mmol) in dry MeOH (20 mL) was stirred under argon, at room temperature, for 18 h. The solvent was vacuum evaporated, the residue was dissolved in CH_2_Cl_2_, washed with water, dried (anh. Na_2_SO_4_), and evaporated to dryness. Flash chromatography on silica gel using a mixture of cyclohexane/EtOAc 1/1 as the eluent provided the title compound **27** (15 mg, 45.5%). Mp.: 202–204 °C (THF-*n*-Pentane). ^1^H NMR (600 MHz, CDCl_3_) δ (ppm) 7.92 (dd, *J* = 8.0, 1.3 Hz, 1H, H-8), 7.49 (td, *J* = 8.4, 1.8 Hz, 1H, H-4′), 7.43–7.34 (m, 2H, H-9, H-6′), 7.19 (dd, *J* = 7.8, 1.3 Hz, 1H, H-10), 7.09 (td, *J* = 7.6, 0.9 Hz, 1H, H-5′), 7.00 (d, *J* = 8.4 Hz, 1H, H-3′), 4.74 (d, *J* = 18.0 Hz, 1H, C*H*H), 4.39 (d, *J* = 17.9 Hz, 1H, CH*H*), 3.82 (s, 3H, OC*H*_3_). ^13^C NMR (151 MHz, CDCl_3_) δ (ppm) 168.4 (*C*O-7), 162.4 (*C*O-2), 159.9 (C-5), 155.7 (C-2′), 132.7 (C-4′), 129.9 (C-6′), 127.2 (C-10b), 127.1 (C-9), 122.4 (C-8), 122.3 (C-10a), 121.8 (C-5′), 119.9 (C-7a), 118.7 (C-10), 111.5 (C-3′), 55.9 (O*C*H_3_), 49.5 (*C*H_2_); HRMS (ESI^+^) *m*/*z* 308.1037 (calcd for C_17_H_14_N_3_O_3_^+^, 308.1030).

*N*-(2-(2-Methoxyphenyl)-4-oxo-3,4-dihydroquinazolin-8-yl)acrylamide (**28**). A solution of **15** (120 mg, 0.34 mmol) and Et_3_N (0.47 mL, 3.34 mmol) in anh. THF (20 mL) was stirred for 18 h at 70 °C, under argon. The solvent was vacuum evaporated, the residue was dissolved in CH_2_Cl_2_, washed with water, dried (anh. Na_2_SO_4_), and evaporated to dryness. Flash chromatography on silica gel using a mixture of cyclohexane/EtOAc 1/1 as the eluent provided 90 mg (83.3%) of the title compound **28**. Mp.: 190–192 °C (THF-*n*-Pentane). ^1^H NMR (400 MHz, CDCl_3_) δ (ppm) 10.98 (brs, 1H, D_2_O exch., N*H*), 9.46 (brs, 1H, D_2_O exch., N*H*CO), 8.95 (dd, *J* = 8.0, 1.3 Hz, 1H, H-7), 8.43 (dd, *J* = 7.9, 1.8 Hz, 1H, H-6′), 7.98 (dd, *J* = 8.0, 1.4 Hz, 1H, H-5), 7.57 (dd, *J* = 8.6, 1.8 Hz, 1H, H-4′), 7.48 (t, *J* = 8.0 Hz, 1H, H-6), 7.21 (t, *J* = 7.6 Hz, 1H, H-5′), 7.12 (d, *J* = 8.4 Hz, 1H, H-3′), 6.78 (dd, *J* = 17.0, 1.2 Hz, 1H, COC*H*=CHH), 6.29 (dd, *J* = 16.9, 1.8 Hz, 1H, COCH=C*H*H), 5.86 (dd, *J* = 9.4, 2.0 Hz, 1H, COCH=CH*H*), 4.09 (s, 3H, OC*H*_3_). ^13^C NMR (101 MHz, CDCl_3_) δ (ppm) 163.6 (NH*C*O), 161.5 (*C*O), 158.1 (C-2′), 150.1 (C-2), 138.5 (C-8a), 134.1 (C-4a), 133.8 (C-4′), 131.8 (CO*C*H=CH_2_), 131.1 (C-6′), 127.8 (COCH=*C*H_2_), 127.2 (C-6), 122.8 (C-7), 122.0 (C-5′), 120.8 (C-8), 120.51(C-5), 119.5 (C-1′), 112.2 (C-3′), 56.4 (O*C*H_3_); HRMS (ESI^+^) *m*/*z* 322.1192 (calcd for C_18_H_16_N_3_O_3_^+^, 322.1186).

*N*-(2-(3-Methoxynaphthalen-2-yl)-4-oxo-3,4-dihydroquinazolin-8-yl)acrylamide (**29**). This compound was synthesized by an analogous procedure as described for the preparation of compound **28**. Yield: 40.7%. Mp.: 210–212 °C (THF-*n*-Pentane). ^1^H NMR (600 MHz, DMSO-*d*_6_) δ (ppm) 12.41 (brs, 1H, D_2_O exch., N*H*), 9.86 (brs, 1H, D_2_O exch., N*H*CO), 8.74 (dd, *J* = 7.9, 1.4 Hz, 1H, H-7), 8.47 (s, 1H, H-1′), 8.00 (d, *J* = 8.2 Hz, 1H, H-8′), 7.92 (d, *J* = 8.2 Hz, 3H, H-5′), 7.88 (dd, *J* = 7.9, 1.4 Hz, 1H, H-5), 7.58 (td, *J* = 8.2, 6.8, 1.3 Hz, 1H, H-6′), 7.56–7.51 (m, 2H, H-6, H-4′), 7.45 (dd, *J* = 8.2, 1.2 Hz, 1H, H-7′), 6.78 (dd, *J* = 16.9, 10.2 Hz, 1H, COC*H*=CHH), 6.31 (dd, *J* = 16.9, 1.7 Hz, 1H, COCH=C*H*H), 5.78 (dd, *J* = 10.3, 1.8 Hz, 1H, COCH=CH*H*), 3.97 (s, 3H, OC*H*_3_). ^13^C NMR (151 MHz, DMSO-*d*_6_) δ (ppm) 163.4 (NH*C*O), 160.9 (*C*O), 154.6 (C-3′), 151.7 (C-2), 139.1 (C-8a), 135.2 (C-4a’), 134.0 (C-4a), 132.2 (CO*C*H=CH_2_), 131.6 (C-1′), 128.4 (C-8′), 127.9 (C-6′), 127.6 (C-8a’), 127.3 (COCH=*C*H_2_), 126.6 (C-6), 126.5 (C-5′), 124.3 (C-7′), 124.2 (C-8), 123.5(C-7), 121.0 (C-2′), 120.0 (C-5), 106.4 (C-4′), 55.9 (O*C*H_3_); HRMS (ESI^+^) *m*/*z* 372.1338 (calcd for C_22_H_18_N_3_O_3_^+^, 372.1343).

2,3-Dihydroxy-*N*-(2-(2-methoxyphenyl)-4-oxo-3,4-dihydroquinazolin-8-yl)propanamide (**30**). To a solution of osmium tetroxide (2.5% in isopropanol) (0.23 mL, 0.0036 mmol) and *N*-methylmorpholine *N*-oxide (14.78 mg, 0.126 mmol) in THF (10 mL), compound **28** (30 mg, 0.09 mmol) was added. The reaction mixture was stirred at rt for 3 days. A saturated NaHSO_3_ solution (0.5 mL) was then added, the mixture was stirred at rt for 60 min, and the volatiles were vacuum evaporated. The residue was then extracted with EtOAc, and the organic phase was washed with a saturated NaHCO_3_ solution, water, and brine, dried (anh. Na_2_SO_4_), and concentrated to dryness. Flash chromatography on silica gel using a mixture of CH_2_Cl_2_/CH_3_OH 100/0–100/5 as the eluent provided 13 mg (40%) of the title compound **30**. Mp.: 210–212 °C (EtOH). IR (Nujol) ν max/cm^−1^: 1669.52 (CO). ^1^H NMR (600 MHz, DMSO-*d*_6_) δ (ppm) 12.19 (brs, 1H, D_2_O exch., N*H*), 10.61 (brs, 1H, D_2_O exch., N*H*CO), 8.80 (dd, *J* = 8.0, 1.4 Hz, 1H, H-7), 7.82 (dd, *J* = 8.0, 1.8 Hz, 1H, H-6′), 7.80 (dd, *J* = 7.8, 1.3 Hz, 1H, H-5), 7.57 (dd, *J* = 8.9, 1.8 Hz, 1H, H-4′), 7.49 (t, *J* = 8.0 Hz, 1H, H-6), 7.23 (d, *J* = 8.4 Hz, 1H, H-3′), 7.12 (td, *J* = 7.5, 1.0 Hz, 1H, H-5′), 6.26 (d, *J* = 5.1 Hz, 1H, CHO*H*), 4.84 (t, *J* = 5.3 Hz, 1H, CH_2_O*H*), 4.12 (td, *J* = 5.1, 3.2 Hz, 1H, C*H*), 3.90 (s, 3H, OC*H*_3_), 3.73–3.63 (m, 2H, C*H*_2_). ^13^C NMR (151 MHz, DMSO-*d*_6_) δ (ppm) 171.2 (NH*C*O), 160.9 (*C*O), 157.4 (C-2′), 151.7 (C-2), 138.3 (C-8a), 133.5 (C-4a), 132.6 (C-4′), 130.5 (C-6′), 126.7 (C-6), 122.1 (C-7), 120. (C-8a, C-5′), 120.60 (C-5), 119.2 (C-1′), 112.1 (C-3′), 73.6 (*C*H), 63.6 (*C*H_2_), 55.9 (O*C*H_3_); HRMS (ESI^+^) *m*/*z* 356.1248 (calcd for C_18_H_18_N_3_O_5_^+^, 356.1241).

2,3-Dihydroxy-*N*-(2-(3-methoxynaphthalen-2-yl)-4-oxo-3,4-dihydroquinazolin-8-yl)propanamide (**31**). This compound was synthesized by an analogous procedure as described for the preparation of compound **30**. Yield: 41%. Mp.: 216–218 °C (EtOH). IR (Nujol) ν max/cm^−1^: 1661.24 (CO). ^1^H NMR (600 MHz, DMSO*-d*_6_) δ (ppm) 10.66 (brs, 1H, D_2_O exch., N*H*CO), 8.81 (dd, *J* = 8.0, 1.4 Hz, 1H, H-7), 8.37 (s, 1H, H-1′), 7.97 (d, *J* = 8.1 Hz, 1H, H-8′), 7.93 (d, *J* = 8.2 Hz, 3H, H-5′), 7.83 (dd, *J* = 8.1, 1.4 Hz, 1H, H-5), 7.59 (dd, *J* = 8.2, 1.3 Hz, 1H, H-6′), 7.56 (s, 1H, H-4′), 7.52 (t, *J* = 8.0 Hz, 1H, H-6), 7.46 (dd, *J* = 7.4, 1.0 Hz, 1H, H-7′), 3.99 (s, 3H, OC*H*_3_), 6.27 (d, *J* = 5.1 Hz, 1H,CHO*H*), 4.85 (t, *J* = 5.9 Hz, 1H, CH_2_O*H*), 4.13 (td, *J* = 5.1, 3.2 Hz, 1H, C*H*), 3.90 (s, 3H, OC*H*_3_), 3.71–3.65 (m, 2H, C*H*_2_). ^13^C NMR (151 MHz, DMSO-*d*_6_) δ (ppm) 171.2 (NH*C*O), 161.0 (*C*O), 154.7 (C-3′), 151.6 (C-2), 138.3 (C-8a), 135.3 (C-4a’), 133.7 (C-4a), 131.0 (C-1′), 128.4 (C-8′), 128.0 (C-6′), 127.6 (C-8a’), 126.9 (C-6), 126.6 (C-5′), 124.5 (C-7′), 124.1 (C-8), 120.9 (C-7), 120.9 (C-5), 119.3 (C-2′), 106.7 (C-4′), 73.5 (*C*H), 63.6 (*C*H_2_), 56.0 (O*C*H_3_); HRMS (ESI^+^) *m*/*z* 406.1405 (calcd for C_22_H_20_N_3_O_5_^+^, 406.1397).

3-(Dimethylamino)-*N*-(2-(2-methoxyphenyl)-4-oxo-3,4-dihydroquinazolin-8-yl)propanamide *(***32***).* This compound was synthesized by an analogous procedure as described for the preparation of compound **17**. Yield: 57%. Mp.: 208–210 °C (THF-*n*-Pentane). IR (Nujol) ν max/cm^−1^: 1669.28 (CO). ^1^H NMR (600 MHz, CDCl_3_) δ (ppm) 10.92 (brs, 1H, D_2_O exch., N*H*CO), 8.93 (dd, *J* = 8.0, 1.5 Hz, 1H, H-7), 8.56 (dd, *J* = 7.9, 1.8 Hz, 1H, H-6′), 7.97 (dd, *J* = 8.0, 1.4 Hz, 1H, H-5), 7.55 (dd, *J* = 8.8, 1.8 Hz, 1H, H-4′), 7.44 (t, *J* = 8.0 Hz, 1H, H-6), 7.17 (d, *J* = 7.6 Hz, 1H, H-5′), 7.11 (d, *J* = 8.4 Hz, 1H, H-5′), 4.08 (s, 3H, OC*H*_3_), 2.70 (t, *J* = 6.1 Hz, 2H, COCH_2_C*H*_2_), 2.67 (t, *J* = 6.1 Hz, 2H, COC*H*_2_CH_2_), 2.37 (s, 6H, (C*H*_3_)_2_). ^13^C NMR (101 MHz, CDCl_3_) δ (ppm) 171.4 (NH*C*O), 161.6 (*C*O), 157.9 (C-2′), 149.6 (C-2), 138.8 (C-8a), 135.1 (C-4a), 133.4 (C-4′), 131.5 (C-6′), 126.9 (C-6), 123.5 (C-7), 121.4 (C-5′), 120.8 (C-8), 120.0 (C-5), 119.8 (C-1′), 112.0 (C-3′), 56.2 (COCH_2_*C*H_2_), 55.6 (O*C*H_3_), 45.3 ((*C*H_3_)_2_), 35.4 (CO*C*H_2_CH_2_); HRMS (ESI^+^) *m*/*z* 367.1770 (calcd for C_20_H_23_N_4_O_3_^+^, 367.1765).

3-(Dimethylamino)-*N*-(2-(3-methoxynaphthalen-2-yl)-4-oxo-3,4-dihydroquinazolin-8-yl)propanamide (**33**). This compound was synthesized by an analogous procedure as described for the preparation of compound **17**. Yield: 60%. Mp.: >230 °C (THF-*n*-Pentane). IR (Nujol) ν max/cm^−1^: 1665.49 (CO). ^1^H NMR (600 MHz, CDCl_3_) δ (ppm) 11.00 (brs, 1H, D_2_O exch., N*H*), 10.68 (brs, 1H, D_2_Oexch., N*H*CO), 8.93 (dd, *J* = 8.1, 1.5 Hz, 1H, H-7), 8.77 (s, 1H, H-1′), 7.97 (dd, *J* = 8.0, 1.4 Hz, 1H, H-5), 7.91 (d, *J* = 8.2 Hz, 1H, H-8′), 7.81 (d, *J* = 8.2 Hz, 1H, H-5′), 7.57 (dd, *J* = 8.1, 1.2 Hz, 1H, H-6′), 7.49–7.42 (m, 2H, H-6, H-7′), 7.33 (s, 1H, H-4′), 4.12 (s, 3H, OC*H*_3_), 2.76–2.67 (m, 4H, COC*H*_2_C*H*_2_), 2.30 (s, 6H, (C*H*_3_)_2_). ^13^C NMR (151 MHz, CDCl_3_) δ (ppm) 171.3 (NH*C*O), 161.6 (*C*O), 154.6 (C-3′), 150.0 (C-2′), 139.1 (C-8a), 135.9 (C-4a’), 135.3 (C-4a), 132.9 (C-1′), 128.7 (C-6′, C-8′), 128.4 (C-8a’), 127.3 (C-6), 126.9 (C-5′), 125.3 (C-7′), 123.7 (C-7), 121.9 (C-8), 121.1 (C-2′), 120.3 (C-5), 107.3 (C-4′), 56.3 (O*C*H_3_), 55.5 (COCH_2_*C*H_2_). 45.2 ((*C*H_3_)_2_), 35.3 (*C*OCH_2_CH_2_); HRMS (ESI^+^) *m*/*z* 417.1929 (calcd for C_24_H_25_N_4_O_3_^+^, 417.1921).

3-(Cyclopropylamino)-*N*-(2-(2-methoxyphenyl)-4-oxo-3,4-dihydroquinazolin-8-yl)propanamide (**34**). This compound was synthesized by an analogous procedure as described for the preparation of compound **17**. Yield: 61%. Mp.: 187–189 °C (THF-*n*-Pentane). IR (Nujol) ν max/cm^−1^: 1666.56 (CO). ^1^H NMR (400 MHz, CDCl_3_) δ (ppm) 9.98 (brs, 1H, D_2_O exch., N*H*CO), 8.80 (dd, *J* = 8.0, 1.4 Hz, 1H, H-7), 8.38 (dd, *J* = 7.9, 1.8 Hz, 1H, H-6′), 7.87 (dd, *J* = 8.0, 1.4 Hz, 1H, H-5), 7.48 (dd, *J* = 8.4, 1.8 Hz, 1H, H-4′), 7.36 (t, *J* = 8.0 Hz, 1H, H-6), 7.11 (d, *J* = 7.6 Hz, 1H, H-5′), 7.02 (dd, *J* = 8.5, 1.0 Hz, 1H, H-3′), 4.00 (s, 3H, OC*H*_3_), 3.14 (t, *J* = 6.1 Hz, 2H, COCH_2_C*H*_2_), 2.71 (t, *J* = 6.1 Hz, 2H, COC*H*_2_CH_2_), 2.17 (t, *J* = 6.8, Hz, 1H, C*H*-cyclopropyl), 0.40–0.33 (m, 4H, C*H*_2_-cyclopropyl). ^13^C NMR (101 MHz, CDCl_3_) δ (ppm) 171.1 (NH*C*O), 161.5 (*C*O), 157.9 (C-2′), 149.8 (C-2), 138.5 (C-8a), 134.4 (C-4a), 133.5 (C-4′), 131.3 (C-6′), 126.9 (C-6), 123.0 (C-7), 121.7 (C-5′), 120.7 (C-8), 120.1 (C-5), 119. (C-1′), 112.0 (C-3′), 56.2 (O*C*H_3_), 45.5 (COCH_2_*C*H_2_), 37.6 (CO*C*H_2_CH_2_), 30.4 (*C*H-cyclopropyl), 6.3 (*C*H_2_-cyclopropyl); HRMS (ESI^+^) *m*/*z* 379.1759 (calcd for C_21_H_23_N_4_O_3_^+^, 379.1765).

3-(Cyclopropylamino)-*N*-(2-(3-methoxynaphthalen-2-yl)-4-oxo-3,4-dihydroquinazolin-8-yl)propanamide (**35**). This compound was synthesized by an analogous procedure as described for the preparation of compound **17**. Yield: 82%. Mp.: >230 °C (THF-*n*-Pentane). IR (Nujol) ν max/cm^−1^: 1664.59 (CO). ^1^H NMR (600 MHz, CDCl_3_) δ (ppm) 10.10 (brs, 1H, D_2_O exch., N*H*CO), 8.88 (m, 2H, H-7, H-1′), 7.96 (dd, *J* = 8.0, 1.4 Hz, H-5), 7.92 (d, *J* = 8.1 Hz, H-8′), 7.80 (d, *J* = 8.2 Hz, 1H, H-5′), 7.57 (td, *J* = 8.1, 1.2 Hz, 1H, H-6′), 7.51–7.44 (m, 2H, H-6, H-7′), 7.32 (s, 1H, H-4′), 4.13 (s, 3H, OC*H*_3_), 3.21 (t, *J* = 6.1 Hz, 2H, COCH_2_C*H*_2_), 2.77 (t, *J* = 6.1 Hz, 2H, COC*H*_2_CH_2_), 2.20 (s, 1H, C*H*-cyclopropyl), 0.43–0.34 (m, 4H, C*H*_2_-cyclopropyl). ^13^C NMR (151 MHz, CDCl_3_) δ (ppm) 171.1 (NH*C*O), 161.5 (*C*O), 154.9 (C-3′), 150.0 (C-2′), 138.8 (C-8a), 136.0(C-4a’), 134.6 (C-4a), 132.9 (C-1′), 129.0 (C-6′), 128.9 (C-8′), 128.5 (C-8a’), 127.3 (C-6), 126.8 (C-5′), 125.3 (C-7′), 123.3 (C-7), 121.0 (C-8), 120.3 C-5, C-2′), 107.4 (C-4′), 56.4 (O*C*H_3_), 45.7 (COCH_2_*C*H_2_), 37.9 (CO*C*H_2_CH_2_) 30.6 (*C*H-cyclopropyl), 6.3 (*C*H_2_-cyclopropyl); HRMS (ESI^+^) *m*/*z* 429.1915 (calcd for C_25_H_25_N_4_O_3_^+^, 429.1912).

3-(Dimethylamino)-*N*-(2-(2-hydroxyphenyl)-4-oxo-3,4-dihydroquinazolin-8-yl)propanamide (**36**). This compound was synthesized by an analogous procedure as described for the preparation of compound **21**. Yield: 41%. Mp.: >230 °C (EtOAc). IR (Nujol) ν max/cm^−1^: 1665.17 (CO). ^1^H NMR (600 MHz, CDCl_3_-MeOD) δ (ppm) 8.10 (dd, *J* = 7.8, 1.4 Hz, 1H, H-7), 7.99 (dd, *J* = 8.1, 1.6 Hz, 1H, H-6′), 7.94 (dd, *J* = 8.0, 1.4 Hz, 1H, H-5), 7.40–7.44 (m, 2H, H-6, H-4′), 7.06 (dd, *J* = 8.3, 1.1 Hz, 1H, H-3′), 6.94 (d, *J* = 7.6 Hz, 1H, H-5′), 3.63 (t, *J* = 6.6 Hz, 2H, COCH_2_C*H*_2_)), 3.21 (t, *J* = 6.5 Hz, 2H, COC*H*_2_CH_2_), 3.04 (s, 6H, (C*H*_3_)_2_). ^13^C NMR (151 MHz, CDCl_3_-MeOD) δ (ppm) 170.2 (NH*C*O), 161.5 (*C*O), 162.3 (C-2′), 153.5 (C-2), 139.8 (C-8a), 135.2 (C-4′), 132.0 (C-4a), 130.0 (C-6′), 129.1 (C-5′), 127.8 (C-6), 124.3 (C-7), 121.6 (C-8a), 120.9 (C-5), 118.3 (C-3′), 114.3 (C-1′), 54.6 (COCH_2_*C*H_2_), 43.8 ((*C*H_3_)_2_), 31.1 (CO*C*H_2_CH_2_); HRMS (ESI^+^) *m*/*z* 353.1617 (calcd for C_19_H_19_N_4_O_3_^+^, 353.1608).

3-(Dimethylamino)-*N*-(2-(3-hydroxynaphthalen-2-yl)-4-oxo-3,4-dihydroquinazolin-8-yl)propanamide (**37**). This compound was synthesized by an analogous procedure as described for the preparation of compound **21**. Yield: 42%. Mp.: 214–216 °C (EtOAc). IR (Nujol) ν max/cm^−1^: 1664.10 (CO). ^1^H NMR (600 MHz, CDCl_3_-MeOD) δ (ppm) 8.72 (s, 1H, H-1′), 8.31 (d, *J* = 7.9 Hz, 1H, H-7), 7.94 (d, *J* = 7.9 Hz, 1H, H-8′), 7.89 (d, *J* = 8.2 Hz, 1H, H-5′), 7.68 (d, *J* = 8.3 Hz, 1H, H-5), 7.49 (dd, *J* = 8.2, 1.2 Hz, 1H, H-6′), 7.38–7.31 (m, 2H, H-6, H-7′), 7.31 (s, 1H, H-4′), 3.34 (m, 2H, COCH_2_C*H*_2_), 3.08 (t, *J* = 6.5 Hz, 2H, COC*H*_2_CH_2_), 2.83 (s, 6H, (C*H*_3_)_2_). ^13^C NMR (151 MHz, CDCl_3_-MeOD) δ (ppm) 170.2 (NH*C*O), 162.7 (*C*O), 156.8 (C-3′), 152.3 (C-2), 138.7 (C-8a), 135.7 (C-4a’), 133.2 (C-4a), 130.4 (C-1′), 128.9 (C-6′), 128.5 (C-8′), 127.6 (C-8a’), 126.6 (C-6, C-5′), 125.8 (C-7′), 122.3 (C-7), 122.1 (C-8), 122.1 (C-2′), 121.0 (C-5), 111.5 (C-4′), 54.4 (COCH_2_*C*H_2_). 43.7 ((*C*H_3_)_2_), 32.2 (CO*C*H_2_CH_2_); HRMS (ESI^+^) *m*/*z* 403.1772 (calcd for C_23_H_23_N_4_O_3_^+^, 403.1765).

### 3.3. Biological Assays and Experiments

#### Cell Viability MTS Assays

The human cancer cell lines Capan-1, HCT-116, NCI-H460, LN-229, HL-60, K-562, and Z-138 were acquired from the American Type Culture Collection (ATCC, Manassas, VA, USA), while the DND-41 cell line was purchased from the Deutsche Sammlung von Mikroorganismen und Zellkulturen (DSMZ Leibniz-Institut, Braunschweig, Germany) and the Hap-1 cell line which was ordered from Horizon Discovery (Horizon Discovery Group, UK). All cell lines were cultured as recommended by the suppliers. Culture media were purchased from Gibco (Gibco Life Technologies, Merelbeke, Belgium) and supplemented with 10% fetal bovine serum (HyClone, Cytiva, MA, USA).

Adherent cell lines were seeded at a density between 500 and 1500 cells per well in 384-well plates (Greiner). After overnight incubation, cells were treated with seven different concentrations of the test compounds, ranging from 100 to 0.006 µM. Docetaxel and Staurosporine were included as positive controls to validate the assay conditions. Untreated cell lines (i.e., without compound treatment) were used as negative controls.

Suspension cell lines were seeded at densities ranging from 2500 to 5000 cells per well in 384-well culture plates containing the test compounds at the same concentration points. Cells were incubated for 72 h with compounds and were then analyzed using the CellTiter 96^®^ AQueous One Solution Cell Proliferation Assay (MTS) reagent (Promega) according to the manufacturer’s instructions. Absorbance of the samples was measured at 490 nm using a SpectraMax Plus 384 (Molecular Devices), and OD values were used to calculate the 50% inhibitory concentration (IC50). Compounds were tested in two independent experiments [27].

Differential scanning fluorimetry (DSF). Thermal melting experiments were measured using an Mx3005p Real Time PCR machine (Stratagene). Proteins were buffered in 10 mM HEPES pH 7.5 and 500 mM NaCl and run in a 96-well plate at a final concentration of 2 μM in 20 μL volume. Compounds were added at a final concentration of 10 μM from stock solutions in DMSO. SYPRO Orange was used as a fluorescence probe at a dilution of 1:1000. Excitation and emission filters for the SYPRO Orange dye were set to 465 nm and 590 nm, respectively. The temperature was raised with a step of 3 °C per minute from 25 °C to 96 °C and fluorescence readings were taken at each interval. Data collection was made in triplicates. For the data analysis, the baselines of the denatured and native states were approximated by a linear fit. The observed temperature shifts, ΔT_m_, were recorded as the difference between the transition midpoints of sample and reference wells containing the protein without the ligand in the same plate and determined by non-linear least squares fit [28].

## 4. Conclusions

A set of 16 newly synthesized 2-aryl-substituted quinazolinones were assessed for their anti-proliferative effects against nine different tumor cell lines. Most of the compounds exhibited moderate to good anti-proliferative activity, with the most promising outcomes observed in the case of phenyl-substituted analogs. All the naphthyl-substituted compounds were found to be inactive against the tested cell lines. In contrast, only the 2-methoxyphenyl-substituted compounds demonstrated interesting activity. Notably, compound 17 exhibited the highest potency, displaying significant efficacy against the Capan-1, HCT-116, Hap-1, NCI-H460, Z-138, and DND-41 cell lines. Considering its potential clinical relevance and therapeutic utility, compound **17** could be further optimized. While the precise mechanism underlying its tumor-suppressing function remains unclear, our results suggest that in the case of the 2-phenyl analog, the side chain significantly enhances its anti-cancer activity.

## Figures and Tables

**Table 1:** Accumulative results of the anti-proliferative activities for all the synthesized compounds.

Comp	IC_50_ (µM)								
	LN-229	Capan-1	Hap-1	HCT-116	NCI-H460	DND-41	HL-60	K-562	Z-138
	Glioblastoma	Pancreatic Adenocarcinoma	Chronic Myeloid Leukemia	Colorectal Carcinoma	Lung Carcinoma	Acute Lymphoblastic Leukemia	Acute Myeloid Leukemia	Chronic Myeloid Leukemia	Non-Hodgkin Lymphoma
**17**	42.6	1.8	2.3	2.2	1.4	5.5	10.6	12.7	2.6
**18**	>100	>100	>100	>100	57.1	>100	98.5	>100	>100
**19**	>100	>100	>100	>100	>100	>100	>100	>100	>100
**20**	>100	>100	>100	>100	>100	>100	>100	>100	>100
**21**	21.8	45.5	34.4	73.1	>100	51.2	53.8	52.4	37.7
**22**	>100	>100	51.3	>100	>100	>100	>100	>100	>100
**25**	32.9	37.0	39.2	9.5	49.8	51.8	48.9	42.2	41.9
**26**	>100	>100	51.3	>100	>100	>100	>100	>100	>100
**30**	>100	>100	51.3	>100	>100	>100	>100	>100	>100
**31**	>100	>100	51.3	>100	>100	>100	>100	>100	>100
**32**	46.7	35.3	48.2	42.0	27.6	37.6	55.0	45.2	15.3
**33**	>100	78.7	70.1	49.1	>100	>100	>100	>100	51.7
**34**	66.4	9.9	11.3	13.1	32.5	10.8	9.6	54.0	11.8
**35**	>100	>100	51.3	>100	>100	>100	>100	>100	>100
**36**	73.6	>100	>100	40.8	>100	95.3	>100	>100	60.1
**37**	67.4	>100	73.8	63.7	>100	>100	>100	>100	>100
Docetaxel (nM)	2.8	4.9	2.0	2.3	2.8	2.9	10.5	19.4	2.0
Staurosporine (nM)	44.7	44.4	37.4	63.7	54.5	59.7	58.3	50.5	48.8

**Table 2:** DSF results on 17 most selected kinases, measured as ΔT_m_ (°C).

	AAK1	CAMKK2	CK2A2	CLK1	DYRK2	HIPK2	GAK	DAPK3	DYRK1A	GSG2	MST3	GSK3B	MAP3K5	PIM3	BMP2K	BMPR2	MEK5
**17**	5.2	5.2	1.8	4.6	2.3	1.1	4.7	2.0	4.0	2.6	0.6	5.1	2.7	3.2	9.0	3.2	5.3
**21**	4.3	4.3	3.9	3.9	2.5	0.8	3.9	2.1	3.2	1.5	0.7	4.7	1.7	6.5	7.0	2.6	2.8
**19**	2.2	2.2	1.0	3.1	1.4	1.2	2.4	4.1	5.0	0.8	3.0	2.4	2.0	3.0	3.3	1.8	5.2
**34**	2.6	2.6	1.6	3.2	1.6	0.7	2.0	1.4	2.7	3.1	0.4	2.4	0.3	2.5	4.5	1.4	3.3
**20**	0.7	0.7	0.9	2.1	1.1	0.5	1.0	0.9	2.2	−0.4	0.3	4.1	0.3	1.0	1.3	1.5	1.5
**35**	1.4	1.4	0.7	2.4	1.4	0.6	1.4	1.2	2.4	0.4	0.2	2.6	0.2	3.5	2.5	0.9	2.2
**18**	2.1	2.1	1.3	3.4	1.5	0.6	2.0	1.3	2.3	−0.1	−0.1	3.9	0.7	1.2	4.8	2.1	2.8
**33**	0.9	0.9	0.9	2.1	1.1	0.6	1.3	2.0	2.0	0.3	0.4	2.1	0.3	2.9	1.8	0.4	1.7
**30**	3.9	3.9	1.8	4.6	3.7	2.0	2.8	2.1	2.8	3.1	0.8	3.0	5.1	3.0	7.0	2.8	6.1
**32**	1.7	1.7	1.4	2.5	1.1	0.5	1.5	1.6	2.0	3.0	0.0	1.5	0.3	1.9	2.8	0.6	2.2
**31**	0.7	0.7	1.2	2.5	1.0	0.5	0.6	0.5	1.4	0.3	0.1	0.8	0.8	1.2	1.3	0.8	2.5
**25**	3.3	3.3	1.3	4.2	3.8	2.3	2.8	1.7	5.3	2.9	0.2	3.7	1.9	2.7	5.0	2.8	6.5
**33**	0.1	0.1	0.9	0.0	0.3	0.6	−0.2	0.4	−0.4	0.5	0.3	0.9	0.0	2.7	0.0	0.2	1.5
**36**	0.2	0.2	2.0	0.3	0.4	0.3	0.3	0.2	1.2	0.8	−0.1	0.5	−0.2	2.8	0.8	0.3	0.6
**Staurosporine**	13.0	23.8	2.8	11.9	7.0	4.6	8.9	16.3	12.8	8.9	9.1	11.2	16.9	19.6	18.3	2.5	13.4
**Silmitasertib**			15.7														
**GW779439X**						11.6											
**GSK626616**																6.6	

## Data Availability

Data are contained within the article and supplementary materials.

## Supplementary Materials

The following supporting information can be downloaded at: https://www.mdpi.com/article/10.3390/molecules28237912/s1, DSF, ^1^H-NMR and ^13^C-NMR are available online.
