# Supplementary material for: Synthesis and Biological Evaluation of 2-Substituted Quinazolin-4(3H)-Ones with Antiproliferative Activities
Source: Molecules. 2023 Dec 2;28(23):7912. doi: 10.3390/molecules28237912 (PMC10707894; doi:10.3390/molecules28237912)
Supplement: Supplementary file 1 [file molecules-28-07912-s001.zip › molecules-2742415-supplementary.pdf]

Supporting Information

Synthesis and Biological Evaluation of Novel 2-Substituted Quinazolin-4(3*H*)-ones with Antiproliferative Activities

Maria Karelou[1], Dionysis Kampasis[1], Amalia. D. Kalampaliki[1], Leentje Persoons[2], Andreas Krämer[3,4], Dominique Schols[2], Stefan Knapp[3,4], Steven De Jonghe[2], Ioannis K. Kostakis[1,*]

1 *Department of Pharmacy, Division of Pharmaceutical Chemistry, National and Kapodistrian University of Athens, Panepistimiopolis, Zografou, 15771 Athens, Greece.*

2 KU Leuven, Department of Microbiology, Immunology and Transplantation, Rega Institute for Medical Research, Laboratory of Virology and Chemotherapy, Herestraat 49, box 1043, 3000 Leuven, Belgium.

3 Institute for Pharmaceutical Chemistry, Department of Biochemistry, Chemistry and Pharmacy, Goethe University Frankfurt, Max-von-Laue-Straße 9, 60438 Frankfurt, Germany

4 Structural Genomics Consortium, Buchmann Institute for Molecular Life Sciences, Goethe University Frankfurt, Max-von-Laue-Straße 15, 60438 Frankfurt, Germany

Correspondence: ikkostakis@pharm.uoa.gr; +30-210-727-4212

Table of contents

Table S1: DFS results 4

Table S2: DFS results 4

Table S3: DFS results 4

Table S4: DFS results 5

Table S5: DFS results 5

Table S6: DFS results 5

Table S7: DFS results 6

Table S8: DFS results 6

Figure S1: ^{1}H NMR spectrum of 3 7

Figure S2: ^{13}C NMR spectrum of 3 7

Figure S3: ^{1}H NMR spectrum of 4 8

Figure S4: ^{13}C NMR spectrum of 4 8

Figure S5: ^{1}H NMR spectrum of 9 9

Figure S6: ^{13}C NMR spectrum of 9 9

Figure S7: ^{1}H NMR spectrum of 10 10

Figure S8: ^{13}C NMR spectrum of 10 10

Figure S9: ^{1}H NMR spectrum of 13 11

Figure S10: ^{13}C NMR spectrum of 13 11

Figure S11: ^{1}H NMR spectrum of 14 12

Figure S12: ^{13}C NMR spectrum of 14 12

Figure S13: ^{1}H NMR spectrum of 15 13

Figure S14: ^{13}C NMR spectrum of 15 13

Figure S15: ^{1}H NMR spectrum of 16 14

Figure S16: ^{13}C NMR spectrum of 16 14

Figure S17: ^{1}H NMR spectrum of 17 15

Figure S18: ^{13}C NMR spectrum of 17 15

Figure S19: ^{1}H NMR spectrum of 18 16

Figure S20: ^{13}C NMR spectrum of 18 16

Figure S21: ^{1}H NMR spectrum of 19 17

Figure S22: ^{13}C NMR spectrum of 19 17

Figure S23: ^{1}H NMR spectrum of 20 18

Figure S24: ^{13}C NMR spectrum of 20 18

Figure S25: ^{1}H NMR spectrum of 21 19

Figure S26: ^{13}C NMR spectrum of 21 19
Figure S27: ^{1}H NMR spectrum of 22 20
Figure S28: ^{13}C NMR spectrum of 22 20
Figure S29: ^{1}H NMR spectrum of 25 21
Figure S30: ^{13}C NMR spectrum of 25 21
Figure S31: ^{1}H NMR spectrum of 26 22
Figure S32: ^{13}C NMR spectrum of 26 22
Figure S33: ^{1}H NMR spectrum of 27 23
Figure S34: ^{13}C NMR spectrum of 27 23
Figure S35: ^{1}H NMR spectrum of 28 24
Figure S36: ^{13}C NMR spectrum of 28 24
Figure S37: ^{1}H NMR spectrum of 29 25
Figure S38: ^{13}C NMR spectrum of 29 25
Figure S39: ^{1}H NMR spectrum of 30 26
Figure S40: ^{13}C NMR spectrum of 30 26
Figure S41: ^{1}H NMR spectrum of 31 27
Figure S42: ^{13}C NMR spectrum of 31 27
Figure S43: ^{1}H NMR spectrum of 32 28
Figure S44: ^{13}C NMR spectrum of 32 28
Figure S45: ^{1}H NMR spectrum of 33 29
Figure S46: ^{13}C NMR spectrum of 33 29
Figure S47: ^{1}H NMR spectrum of 34 30
Figure S48: ^{13}C NMR spectrum of 34 30
Figure S49: ^{1}H NMR spectrum of 35 31
Figure S50: ^{13}C NMR spectrum of 35 31
Figure S51: ^{1}H NMR spectrum of 36 32
Figure S52: ^{13}C NMR spectrum of 36 32

Cpd Name	Kinases											
	CAMK4A	CDC42BPAA	CDK2A	CDKL1A	CHEK2A	CLK1A	CLK3A	DYRK2A	CSNK1EA	EPHA4A	FECHHSF	HIPK2HSF
**17**	-0.3	-1.3	0.8	0.4	1.4	4.6	-0.1	2.3	1.2	0.3	0.2	1.1
**21**	-0.4	-0.8	1.2	0.5	1.2	3.9	0.8	2.5	1.4	-0.1	-2.4	0.8
**19**	0.7	-0.5	1.4	0.7	1.7	3.1	1.6	1.4	0.7	0.1	0.8	1.2
**34**	-0.5	-1.2	0.5	0.1	0.6	3.2	0.1	1.6	1.3	-0.2	2.1	0.7
**20**	-0.4	-0.7	0.0	0.3	0.5	2.1	-0.1	1.1	0.6	-0.1	2.4	0.5
**35**	-0.5	-0.9	0.3	0.4	0.4	2.4	0.0	1.4	1.4	0.1	6.3	0.6
**18**	-0.4	-0.7	0.2	0.5	1.4	3.4	0.0	1.5	1.3	0.4	4.0	0.6
**33**	-0.3	-0.7	0.5	0.4	0.3	2.1	0.4	1.1	1.2	0.0	4.5	0.6
**30**	0.2	-0.4	0.9	0.3	2.6	4.6	1.5	3.7	2.1	0.3	3.1	2.0
**32**	-0.5	-1.2	0.5	0.2	0.4	2.5	0.0	1.1	1.1	-0.4	0.2	0.5
**31**	-0.7	-0.2	0.2	0.6	0.5	2.5	0.5	1.0	0.5	-0.4	1.5	0.5
**25**	-0.6	-0.5	0.6	0.5	0.9	4.2	1.3	3.8	2.2	0.0	2.1	2.3
**37**	0.0	-0.3	0.6	0.7	0.0	0.0	-0.3	0.3	0.9	0.1	0.8	0.6
**36**	-0.3	-0.9	0.4	0.3	-0.1	0.3	-0.4	0.4	1.0	0.0	-0.9	0.3
**Staurosporine**	7.1	2.6	15.2	4.2	16.5	11.9	5.3	7.0	3.1	5.6	2.1	4.6

Table S1: DFS results

Cpd Name	Kinases										
	MSSK1A	NQO2A	DAPK1A	DCAMKL1A	DMPK1A	EPHA5A	EPHA7A	EPHB3A	FGFR1B	FGFR2A	GAKA
**17**	1.0	-0.1	1.3	-0.7	-0.2	0.3	-3.2	1.0	0.2	0.0	4.7
**21**	0.2	0.2	0.4	-1.4	-0.4	0.1	-2.7	0.3	0.3	0.1	3.9
**19**	0.3	0.4	0.8	-1.1	-0.5	0.3	-2.3	1.1	0.5	0.4	2.4
**34**	0.2	-0.5	-0.1	-1.5	-0.4	-0.7	-3.9	0.5	0.1	0.0	2.0
**20**	0.1	0.2	0.0	0.3	-0.3	-0.3	-3.4	1.0	0.1	-0.3	1.0
**35**	0.0	0.0	0.2	-0.7	0.1	-0.1	-3.0	1.0	0.3	0.1	1.4
**18**	0.5	0.0	0.8	0.2	0.5	-0.1	-3.0	1.3	0.2	-0.1	2.0
**33**	0.1	0.4	0.9	-0.4	1.6	0.2	-2.3	0.5	0.3	-0.1	1.3
**30**	0.4	-0.1	0.8	0.2	0.6	0.4	-2.7	0.6	0.1	-0.4	2.8
**32**	0.1	-0.2	-0.2	-1.3	-0.6	-0.3	-3.8	0.2	0.2	-0.2	1.5
**31**	0.0	0.3	0.1	0.0	-0.2	-0.5	-2.5	-0.4	-0.2	-0.4	0.6
**25**	0.0	-0.4	1.0	-0.5	0.1	-0.5	-2.7	0.6	0.0	-0.4	2.8
**37**	0.0	0.0	-0.4	-2.0	-0.1	-0.3	-2.3	0.6	0.0	-0.1	-0.2
**36**	0.3	0.3	-0.5	-2.5	-0.9	-0.4	-4.0	3.1	0.2	-0.1	0.3
**Staurosporine**	3.7	-0.2	9.0	11.8	8.2	7.7	9.5	5.5	6.4	8.4	8.9

Table S2: DFS results

Cpd Name	Kinases										
	DAPK3A	DYRK1AA	FESA	GSG2A	TTKA	EPHA2A	MAPK1A	MERTKA	MST3A	MST4A	GPRK5A
**17**	2.0	4.0	0.1	2.6	0.2	1.1	-0.1	-0.7	0.6	0.3	1.0
**21**	2.1	3.2	0.2	1.5	-0.2	0.8	-0.2	-0.6	0.7	0.3	1.5
**19**	4.1	5.0	0.9	0.8	0.3	0.8	-0.4	-0.5	3.0	1.1	1.2
**34**	1.4	2.7	0.0	3.1	-0.3	0.7	-0.4	-1.1	0.4	0.3	0.5
**20**	0.9	2.2	0.0	-0.4	-0.2	0.5	-0.5	-0.8	0.3	0.0	0.3
**35**	1.2	2.4	-0.1	0.4	-0.4	0.6	-0.3	-0.8	0.2	0.0	0.7
**18**	1.3	2.3	0.5	-0.1	-1.2	0.7	-0.2	-0.6	-0.1	-0.2	0.4
**33**	2.0	2.0	0.1	0.3	-0.1	0.7	-0.3	-0.3	0.4	0.1	0.5
**30**	2.1	2.8	0.0	3.1	0.2	0.8	-0.2	0.0	0.8	0.3	0.7
**32**	1.6	2.0	-0.2	3.0	-0.4	0.6	-0.3	-1.0	0.0	-0.2	0.3
**31**	0.5	1.4	-0.3	0.3	-2.0	0.2	-0.3	-0.7	0.1	0.1	-0.2
**25**	1.7	5.3	0.0	2.9	-0.1	0.7	-0.2	-0.5	0.2	0.2	0.7
**37**	0.4	-0.4	-0.1	0.5	-0.6	0.6	-0.4	-1.0	0.3	0.1	0.3
**36**	0.2	1.2	0.0	0.8	-0.2	0.5	-0.2	-1.5	-0.1	0.5	-0.1
**Staurosporine**	16.3	12.8	7.7	2.3	8.4	7.4	1.3	5.7	9.1	7.8	8.0

Table S3: DFS results

Cpd Name	Kinases										
	GSK3BB	MAP2K4A	MAP2K6A	MAP3K5A	MAPK10A	MAPK13A	MAPK14A	MAPK8B	MAPK9A	MAP2K7A	TLK1A
**17**	5.1	1.0	0.2	2.7	-1.2	0.7	-0.2	1.5	0.8	-0.8	-1.7
**21**	4.7	2.0	0.3	1.7	-0.2	0.4	0.1	2.5	1.2	-0.7	-1.0
**19**	2.4	2.3	0.7	2.0	-2.1	0.5	0.4	1.1	-0.5	0.9	-0.4
**34**	2.4	0.8	0.2	0.3	-3.9	0.4	-0.4	-0.8	-0.5	-1.0	-2.1
**20**	4.1	0.9	0.2	0.3	-3.3	0.5	-0.5	-0.8	-0.4	-0.2	-1.7
**35**	2.6	0.9	0.2	0.2	-3.1	0.4	-0.3	-0.4	-0.3	-0.3	-1.8
**18**	3.9	0.8	0.3	0.7	-1.1	0.8	-0.3	0.9	0.2	0.1	-1.7
**33**	2.1	0.6	0.2	0.3	-2.5	0.5	0.0	0.0	-0.4	-0.1	-1.6
**30**	3.0	1.1	0.2	5.1	-0.6	0.4	0.1	1.8	-0.2	0.1	-1.1
**32**	1.5	1.3	0.0	0.3	-3.9	0.3	-0.5	-0.4	-0.5	-0.4	-2.0
**31**	0.8	0.2	0.1	0.8	-2.3	0.3	-0.4	0.6	-0.5	-0.8	-1.8
**25**	3.7	1.2	0.3	1.9	-1.2	0.5	-0.1	1.6	0.4	-0.6	-1.3
**37**	0.9	0.6	0.1	0.0	-2.8	0.4	-0.3	-0.6	-0.9	-0.7	-1.6
**36**	0.5	0.4	0.0	-0.2	-4.9	0.4	-0.6	-1.3	-0.7	-1.5	-1.7
**Staurosporine**	11.2	11.5	11.8	16.9	4.7	6.5	0.9	8.0	3.2	8.3	8.5

Table S4: DFS results

Cpd Name	Kinases										
	MARK3A	MARK4A	MELKA	NEK1A	NEK2A	NEK7A_2	OSR1A	PAK1A	PIM3A	FLT1A	PCTK1A
**17**	-1.0	-0.6	1.8	0.1	0.4	-2.6	-0.2	-0.1	3.2	2.3	1.0
**21**	-0.6	-0.3	0.9	-0.5	1.1	-1.2	0.2	1.2	6.5	1.3	1.2
**19**	0.3	-0.4	1.4	0.5	1.7	0.3	1.0	1.2	3.0	1.3	0.2
**34**	-0.3	-0.5	1.6	0.1	0.5	-2.2	-0.4	0.3	2.5	1.4	0.4
**20**	-1.1	-1.1	0.9	0.0	0.4	-1.8	-0.2	0.4	1.0	1.3	0.1
**35**	-0.8	-0.6	0.8	0.2	0.6	-1.4	-0.4	0.4	3.5	1.2	0.4
**18**	-0.8	-1.0	0.7	0.5	0.8	-1.4	-0.1	0.6	1.2	1.4	0.6
**33**	-0.7	-0.9	1.0	-0.5	0.4	-1.8	-0.1	0.4	2.9	1.2	0.4
**30**	-0.4	0.0	1.7	0.2	0.8	-0.5	0.3	0.4	3.0	2.5	0.6
**32**	-1.6	-0.8	1.0	-0.1	1.5	-2.0	-0.6	0.1	1.9	1.5	0.2
**31**	-0.3	-0.7	0.4	-0.2	0.5	-1.1	-0.2	0.1	1.2	1.1	-0.1
**25**	-0.7	-0.2	1.0	0.0	0.6	-0.6	-0.1	0.3	2.7	1.0	0.6
**37**	-0.9	-1.0	0.1	-0.1	1.1	-1.2	-0.1	1.1	2.7	0.2	0.4
**36**	-0.7	-0.6	-0.1	-0.1	0.5	-2.2	-0.6	0.1	2.8	0.5	0.4
**Staurosporine**	18.1	15.8	12.8	-0.2	4.6	1.4	6.1	7.8	19.6	13.5	9.3

Table S5: DFS results

Cpd Name	Kinases										
	PKMYT1A	PLK4A	RPS6KA5A	SLKA	SRCA	SRPK1A	PAK4A	PHKG2A	PIM1A	RPS6KA1A	STK10A	MAPK7
**17**	-0.3	1.8	0.6	-1.2	0.2	0.3	-0.2	2.3	2.6	1.3	-1.0	-0.2
**21**	0.6	1.3	0.3	-0.3	0.5	0.3	-0.3	1.0	5.4	0.6	-0.2	-0.2
**19**	0.2	0.9	3.4	1.1	0.4	0.8	-0.3	1.4	2.2	0.3	1.2	-0.2
**34**	0.1	1.3	2.2	-1.3	0.2	0.0	-0.5	1.1	1.3	0.5	-0.9	0.0
**20**	-0.9	1.1	1.0	-1.0	-0.4	-0.1	-1.1	0.1	1.0	-0.2	-1.2	-0.1
**35**	0.0	1.1	3.0	-1.2	0.0	0.0	-0.9	0.3	1.6	0.3	-0.9	-0.2
**18**	-0.3	1.0	-1.0	-1.2	0.0	0.3	-0.8	0.2	1.1	0.7	-0.8	-0.4
**33**	-0.2	0.5	2.7	-0.7	-0.1	0.2	-0.3	0.5	1.4	0.2	-0.9	0.1
**30**	-0.1	2.5	2.5	-0.4	0.3	0.5	-0.1	2.5	3.6	2.2	-0.6	0.0
**32**	0.0	0.8	1.4	-1.3	0.1	0.0	-0.5	0.9	1.6	0.3	-1.2	0.2
**31**	-1.1	0.5	0.7	-1.1	-0.3	-0.1	-0.5	0.3	1.3	0.3	-0.9	0.2
**25**	-0.1	1.6	4.1	-0.8	0.1	0.2	-0.2	1.3	2.8	1.3	-1.2	-0.1
**37**	0.1	0.2	-0.4	-0.5	0.1	0.2	-0.4	0.2	1.3	0.1	-0.5	-0.2
**36**	0.1	0.2	-0.2	-1.3	0.3	-0.2	-0.1	-0.3	1.3	-0.1	-1.2	0.3
**Staurosporine**	0.5	21.5	15.3	15.3	5.4	6.7	11.3	22.0	12.3	3.2	22.7	

Table S6: DFS results

Cpd Name	Kinases											
	AKT3A	BMP2KA	BMPR2A	CAMK1DA	CAMK1GA	STK17AA	STK17BA	STK38LA	STK39A	BRD4A	STK3A	MEK5
**17**	0.1	9.0	3.2	-2.2	-0.1	3.2	-2.2	-0.3	-0.8	-1.3	0.8	5.3
**21**	0.2	7.0	2.6	-2.4	0.2	2.2	-1.2	0.0	-0.2	-1.0	1.4	2.8
**19**	0.4	3.3	1.8	-1.5	1.1	1.4	-0.7	0.3	0.5	-0.4	2.1	5.2
**34**	0.2	4.5	1.4	-2.4	-0.3	1.6	-2.8	-0.4	-0.8	-0.9	0.5	3.3
**20**	-0.1	1.3	1.5	-1.8	-0.3	0.1	-3.2	-0.2	-0.6	-1.9	0.0	1.5
**35**	0.0	2.5	0.9	-2.2	0.4	1.1	-2.8	-0.3	-0.6	-1.0	1.0	2.2
**18**	0.3	4.8	2.1	-2.0	0.1	0.8	-2.1	-0.4	-0.9	-1.2	0.5	2.8
**33**	0.2	1.8	0.4	-2.1	0.3	1.8	-1.7	-0.3	-0.9	-1.3	0.4	1.7
**30**	0.0	7.0	2.8	-1.9	0.1	3.8	-0.6	-0.1	-0.7	-2.5	0.2	6.1
**32**	0.2	2.8	0.6	-2.6	0.0	1.5	-3.2	-0.2	-0.9	-0.6	0.0	2.2
**31**	0.0	1.3	0.8	-1.9	-0.3	0.7	-0.6	0.1	-0.6	-1.1	-1.6	2.5
**25**	0.0	5.0	2.8	-1.8	-0.1	2.6	0.1	-0.1	-0.8	-0.7	1.0	6.5
**37**	0.3	0.0	0.2	-1.0	-0.8	-0.4	-2.6	-0.1	-0.9	-0.7	0.0	1.5
**36**	0.2	0.8	0.3	-3.4	-1.1	-0.5	-4.1	-0.2	-0.7	-1.2	-0.7	0.6
**Staurosporine**	6.8	18.3	2.5	9.2	10.7	12.3	11.1	0.3	8.7	0.5	16.1	

Table S7: DFS results

Cpd Name	Kinases											
	STK4A	STK6A	ULK3A	VRK1A	WNK1A	BRPF1B	EPHB1A	MAPK15HS	MAPKAPK2A	TAF1A	CSNK1G2	MEK1
**17**	1.4	1.6	4.2	0.8	-0.5	-1.2	0.7	2.3	0.1	0.2	1.8	0.2
**21**	1.4	0.8	1.8	0.7	-0.8	-0.7	0.3	1.4	0.1	0.3	1.8	0.1
**19**	1.7	1.6	3.0	1.1	0.4	-0.2	0.7	0.7	0.6	0.5	1.1	0.1
**34**	0.3	0.0	2.1	0.5	-0.6	-1.3	0.1	1.0	-0.2	0.1	1.4	0.0
**20**	-0.3	0.3	1.3	0.5	-0.3	-1.7	0.9	-0.4	-0.2	0.0	0.8	-0.1
**35**	0.3	0.0	1.5	0.4	-0.4	-1.0	0.6	-0.2	-0.2	0.3	0.7	0.2
**18**	0.5	0.7	1.5	0.4	-0.1	-1.3	1.0	0.0	0.0	0.2	1.0	-0.3
**33**	0.0	0.3	1.6	0.6	-0.4	-1.2	0.5	0.6	-0.1	0.0	0.9	0.1
**30**	1.5	1.6	5.4	0.8	-0.4	-1.2	0.3	2.1	0.4	0.3	2.5	0.3
**32**	0.2	-0.4	1.7	0.4	-0.6	-1.3	0.1	1.0	-0.2	0.0	0.9	-0.2
**31**	-0.5	-0.1	1.7	0.3	-0.4	-1.8	-0.4	0.1	-0.2	-1.1	0.7	0.3
**25**	1.3	1.0	4.1	0.7	-0.5	-1.5	0.4	1.7	0.0	0.2	2.2	0.2
**37**	0.4	-0.3	0.9	-0.2	-0.3	-1.1	-0.1	-0.4	-0.1	-0.1	0.6	0.2
**36**	-0.2	-0.5	0.8	0.2	-0.4	-0.8	0.1	0.2	-0.3	0.2	0.9	0.3
**Staurosporine**	15.7	16.9	19.0	3.2	1.2	0.0	6.2	14.2	3.5	0.0	2.0	

Table S8: DFS results

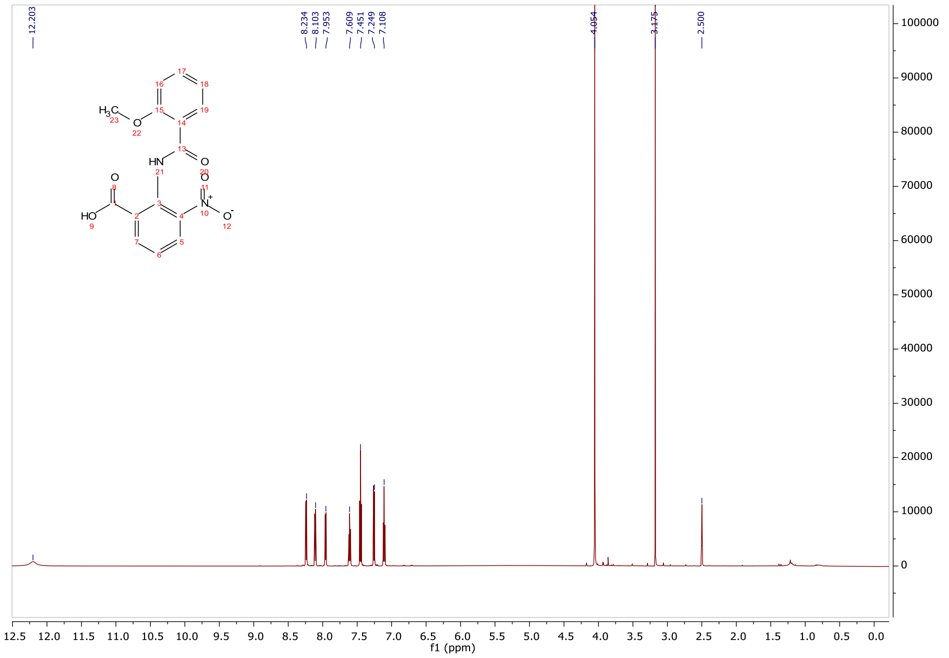

Figure S1: ^{1}H NMR spectrum of 3

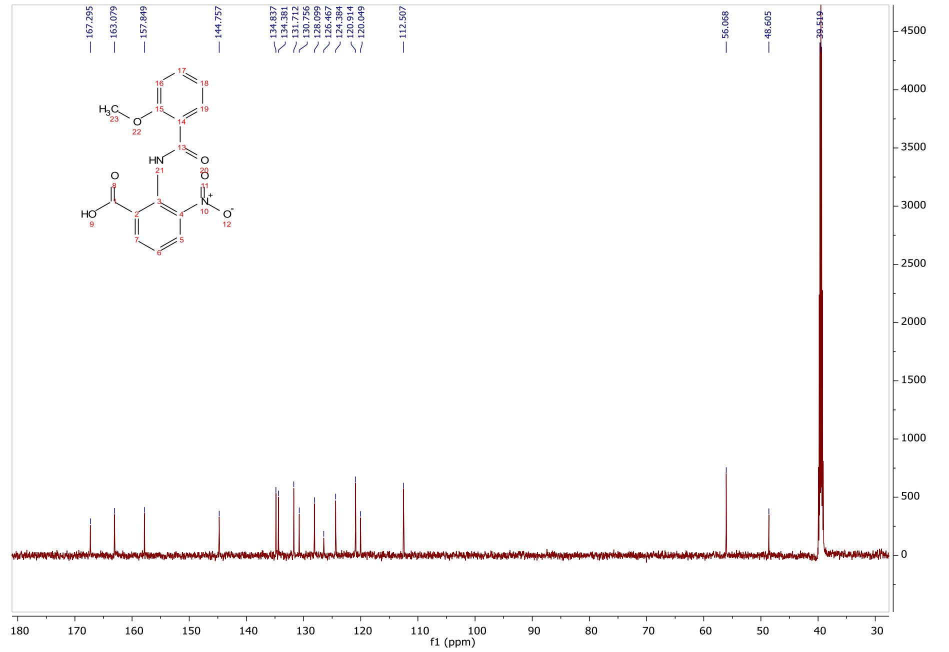

Figure S2: ^{13}C NMR spectrum of 3

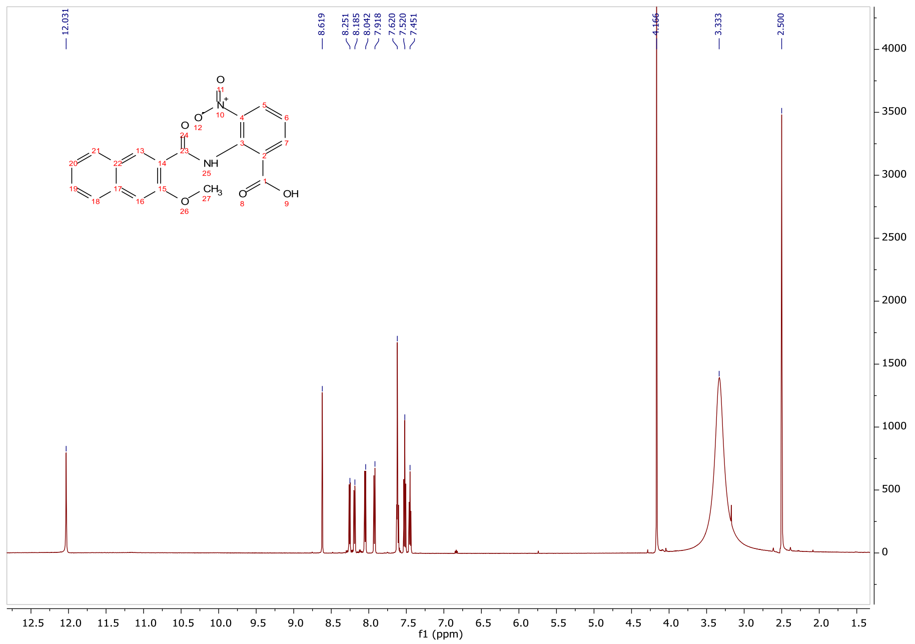

Figure S3: ^{1}H NMR spectrum of 4

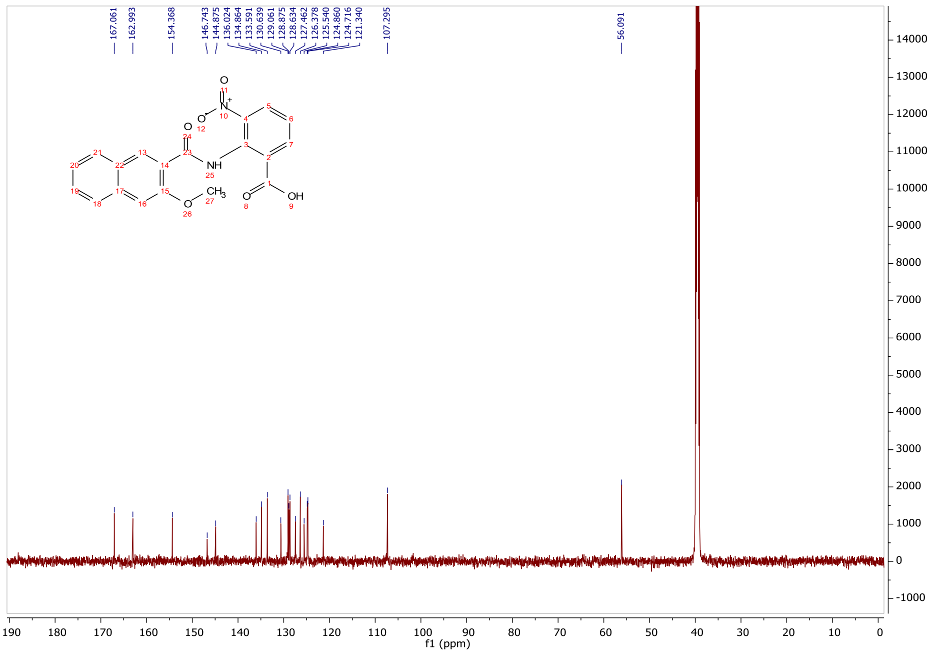

Figure S4: ^{13}C NMR spectrum of 4

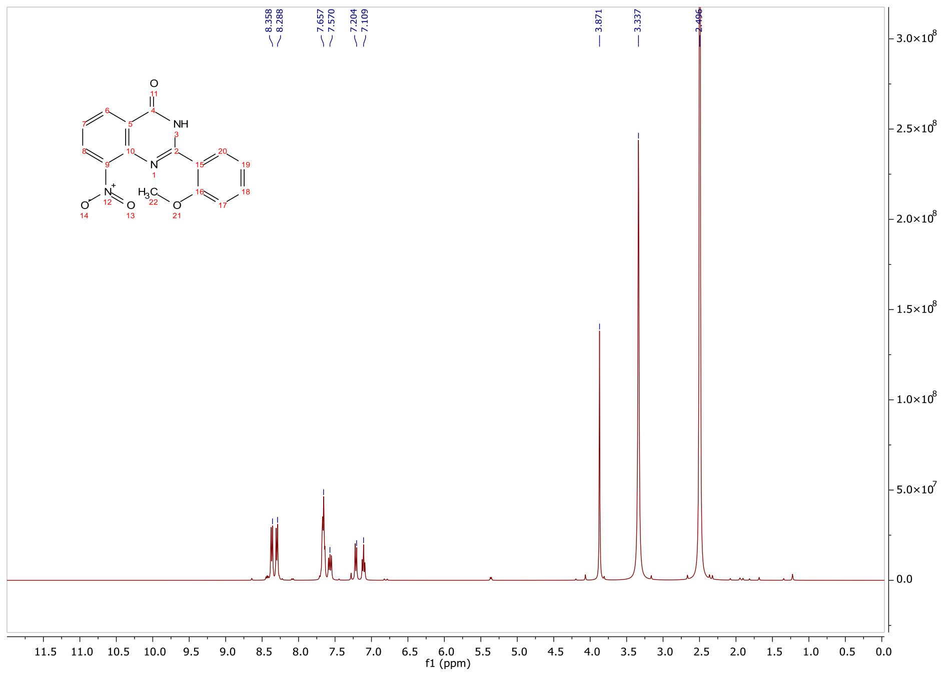

Figure S5: ^{1}H NMR spectrum of 9

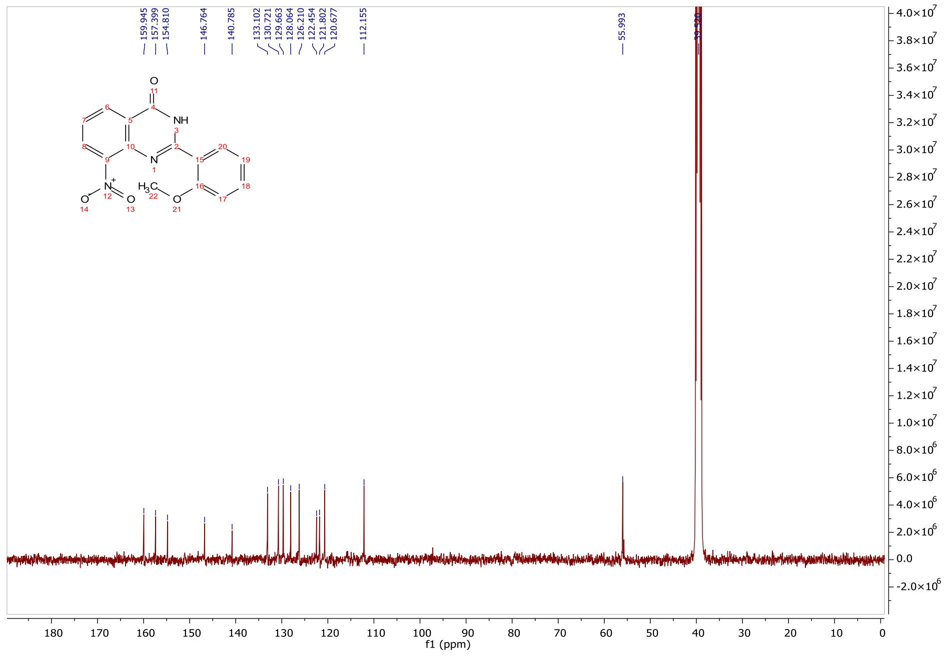

Figure S6: ^{13}C NMR spectrum of 9

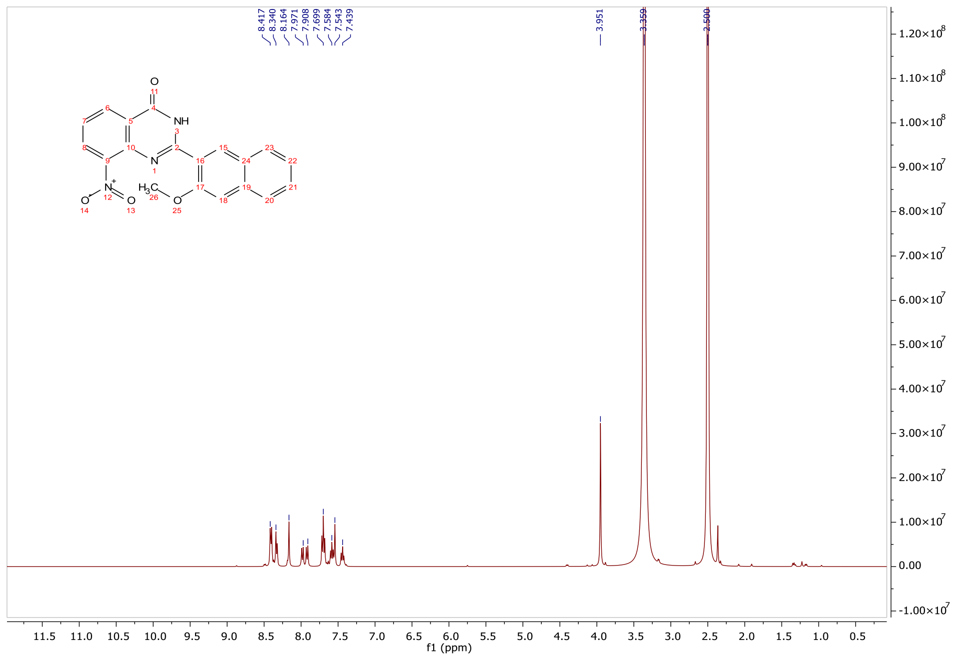

Figure S7: ^{1}H NMR spectrum of 10

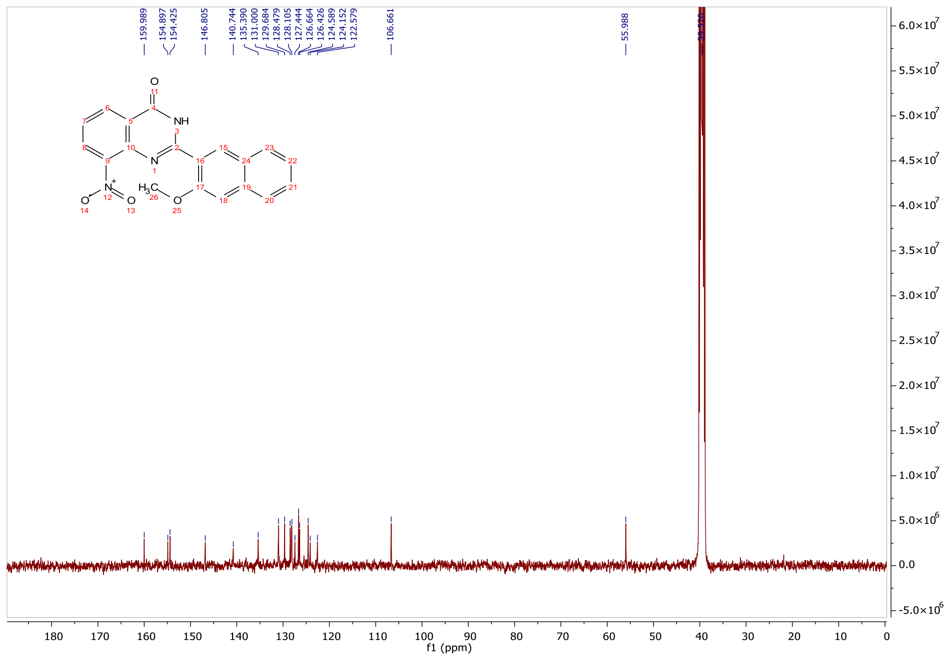

Figure S8: ^{13}C NMR spectrum of 10

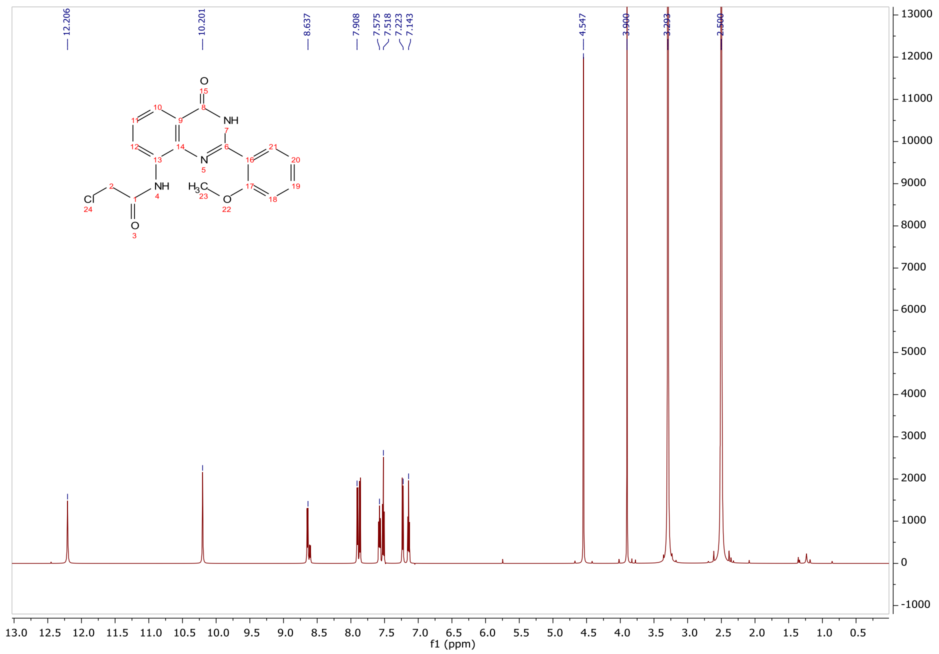

Figure S9: ^{1}H NMR spectrum of 13

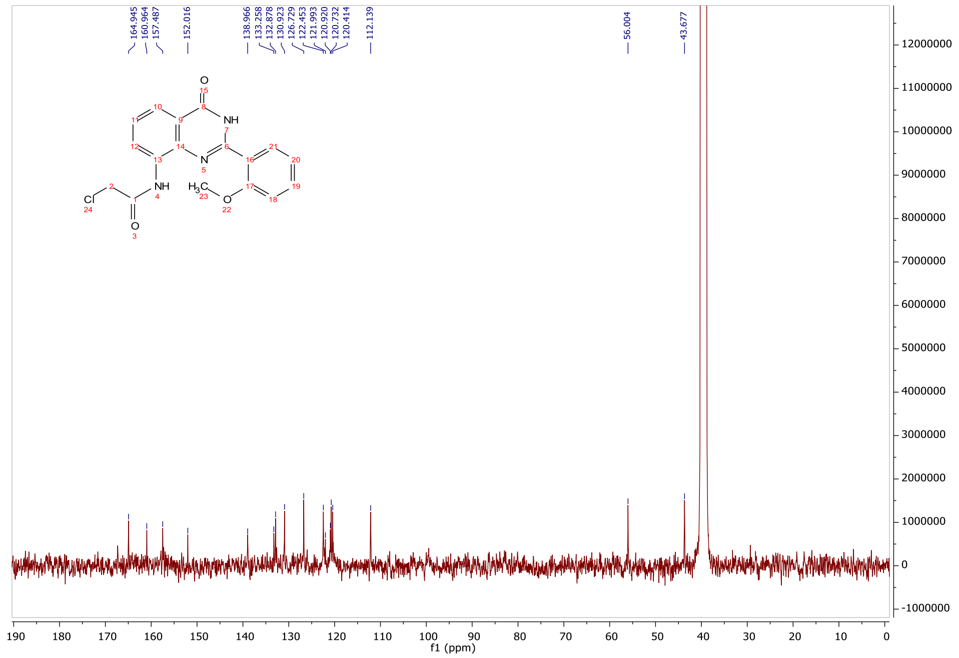

Figure S10: ^{13}C NMR spectrum of 13

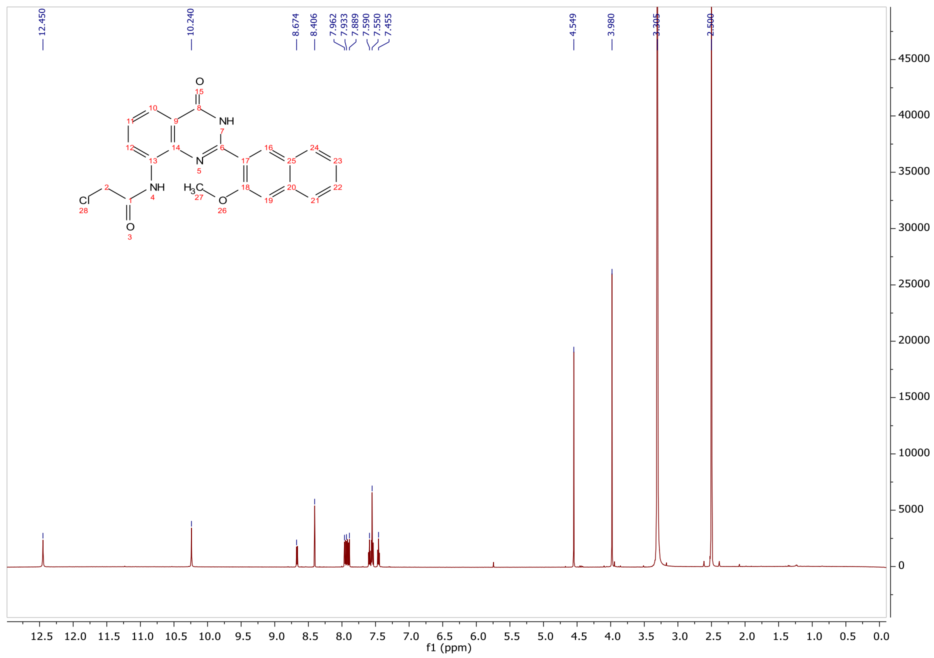

Figure S11: ^{1}H NMR spectrum of 14

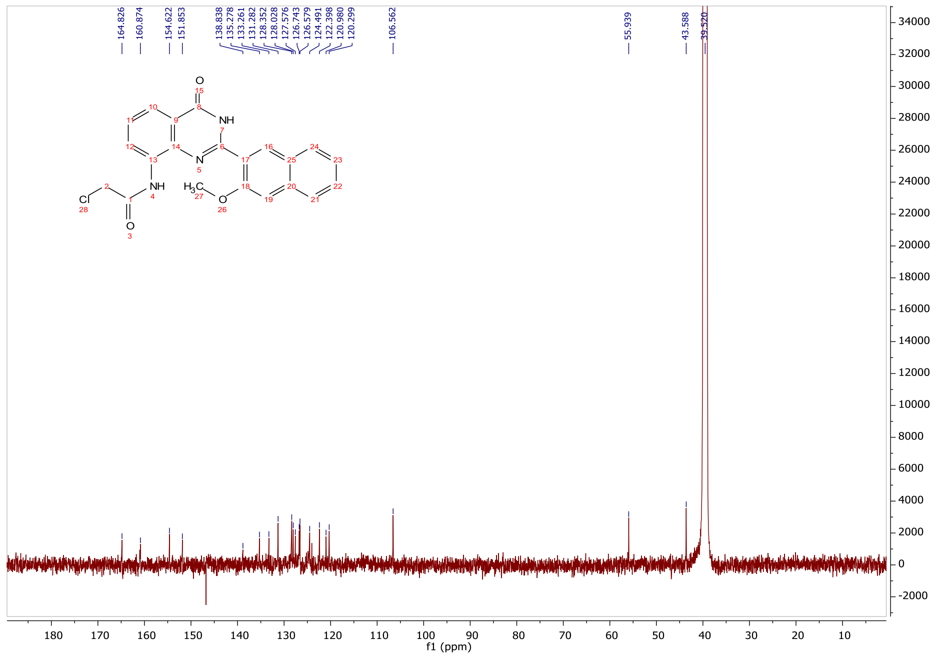

Figure S12: ^{13}C NMR spectrum of 14

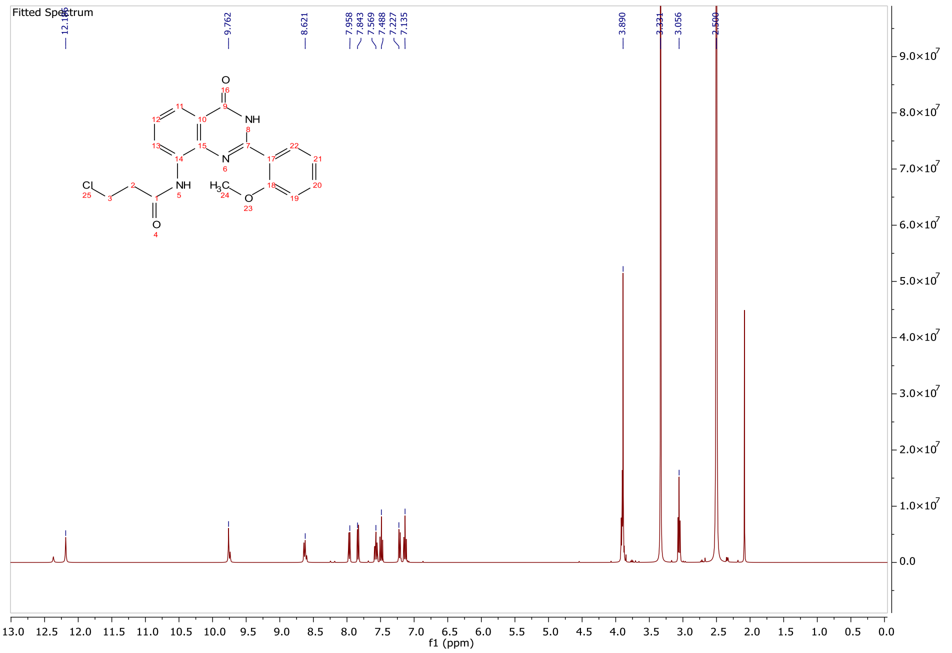

Figure S13: ^{1}H NMR spectrum of 15

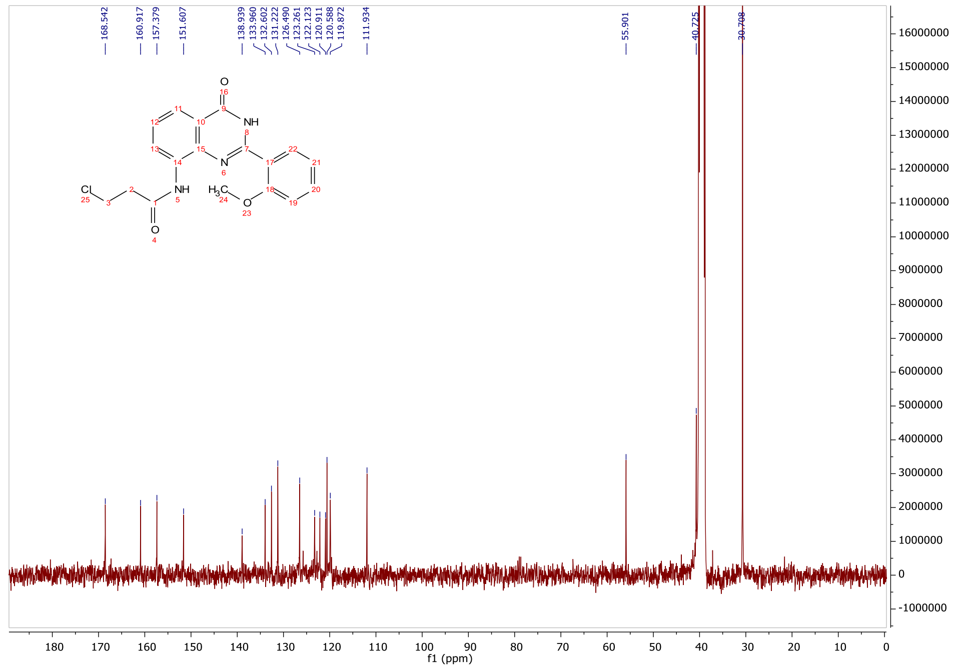

Figure S14: ^{13}C NMR spectrum of 15

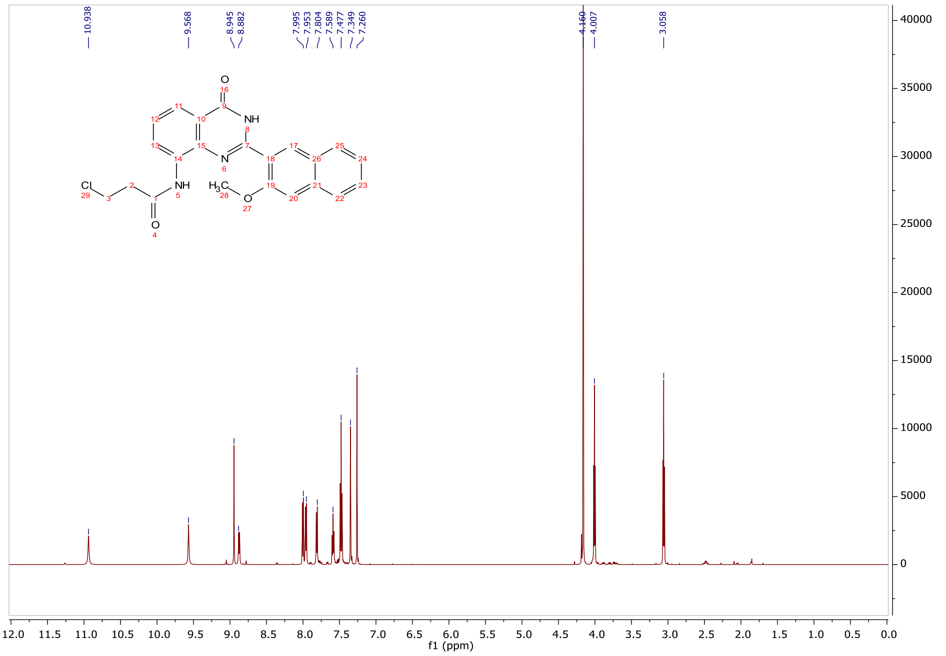

Figure S15: ^{1}H NMR spectrum of 16

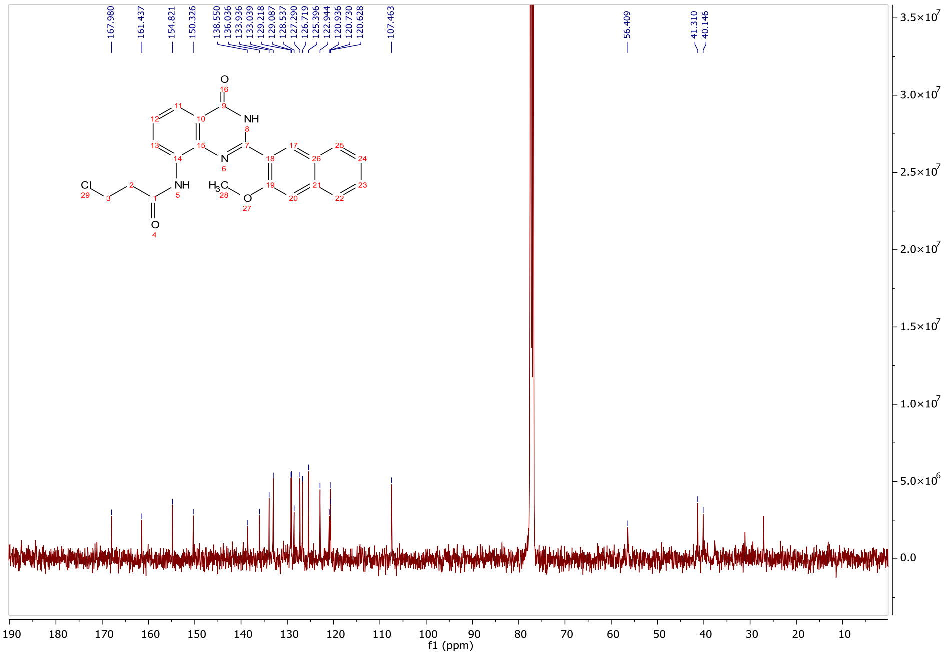

Figure S16: ^{13}C NMR spectrum of 16

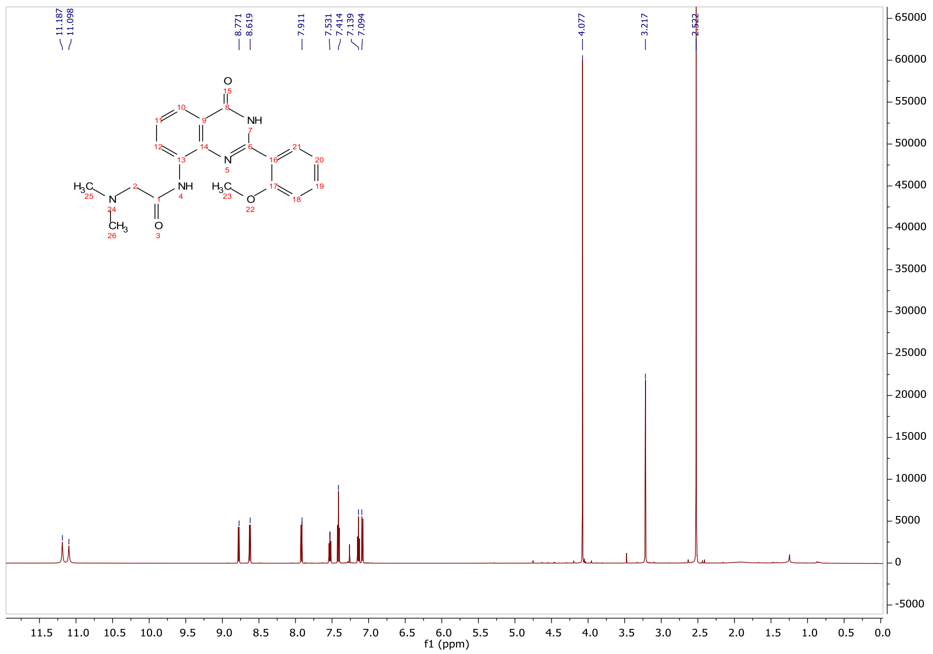

Figure S17: ^{1}H NMR spectrum of 17

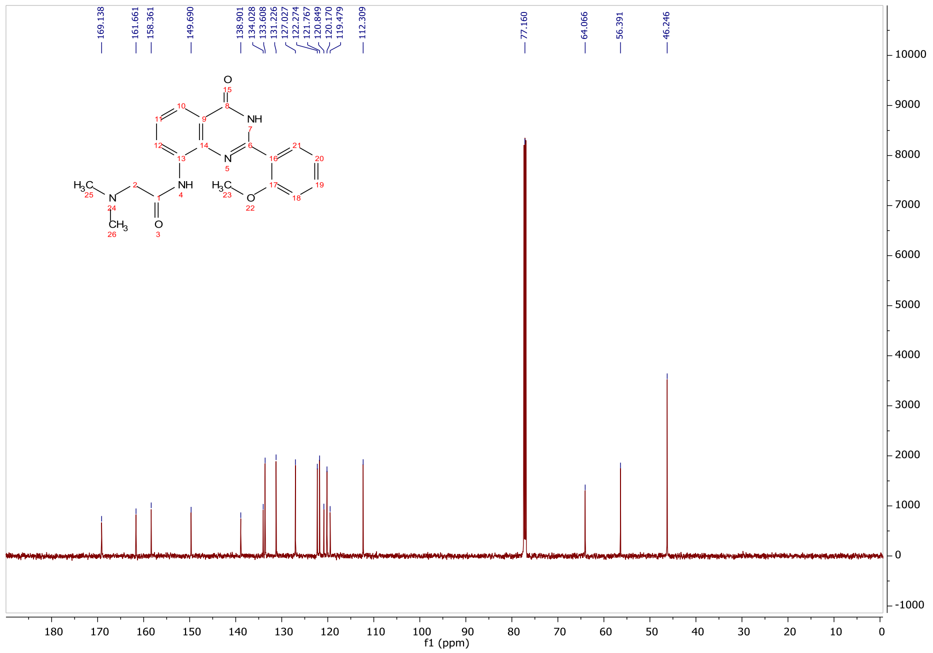

Figure S18: ^{13}C NMR spectrum of 17

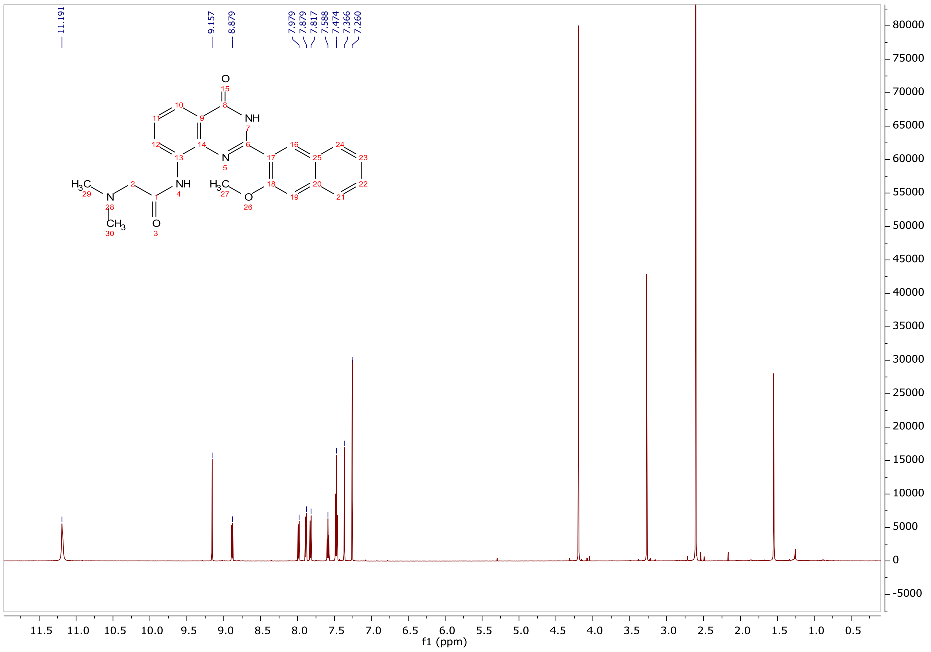

Figure S19: ^{1}H NMR spectrum of 18

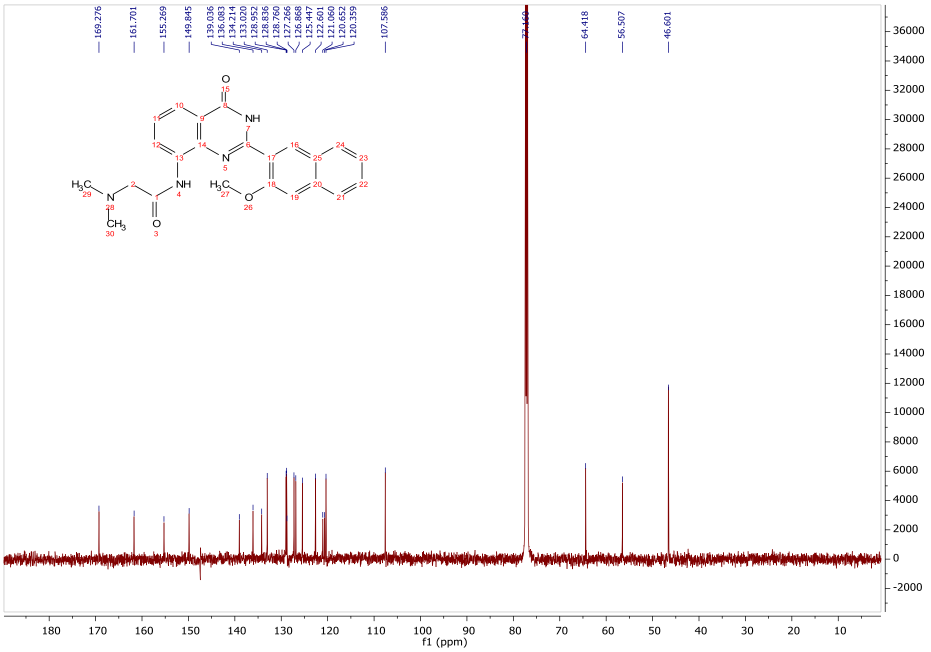

Figure S20: ^{13}C NMR spectrum of 18

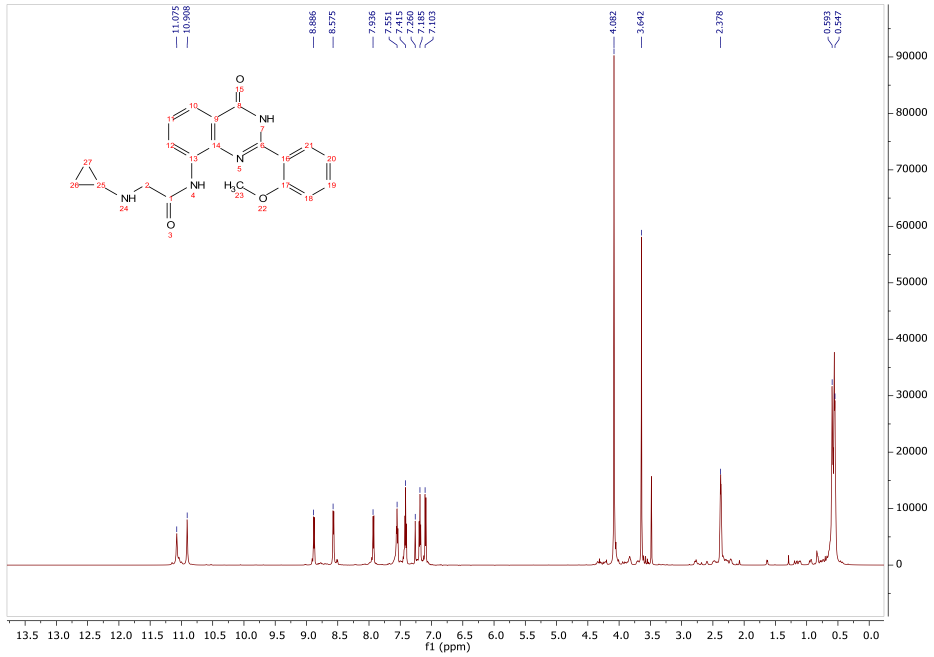

Figure S21: ^{1}H NMR spectrum of 19

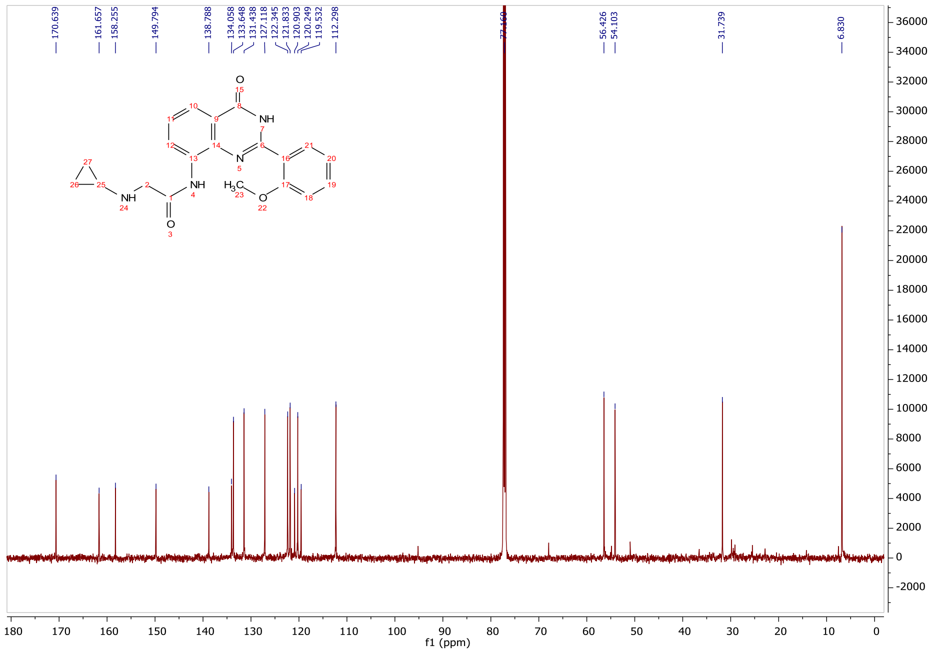

Figure S22: ^{13}C NMR spectrum of 19

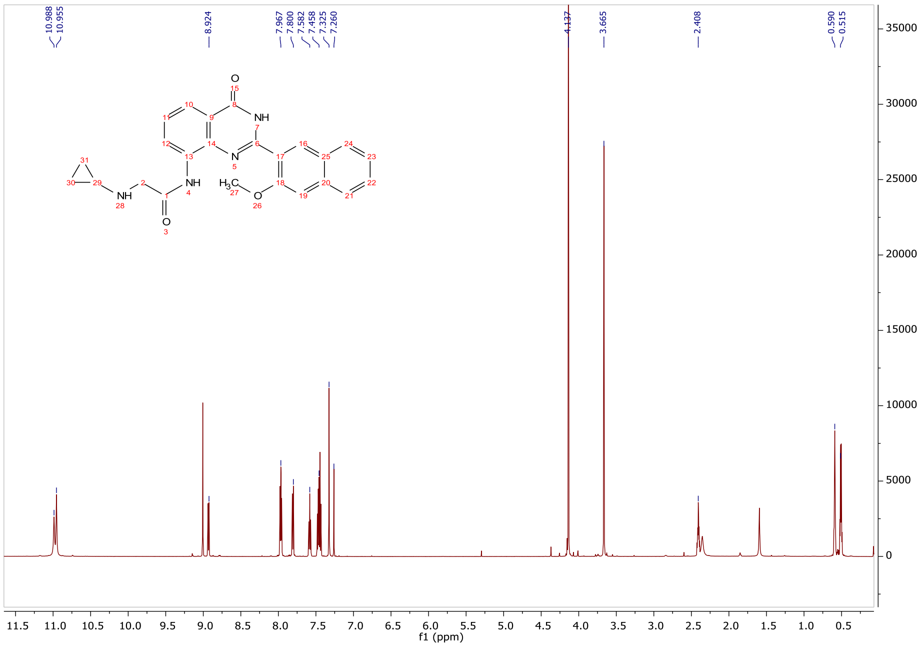

Figure S23: ^{1}H NMR spectrum of 20

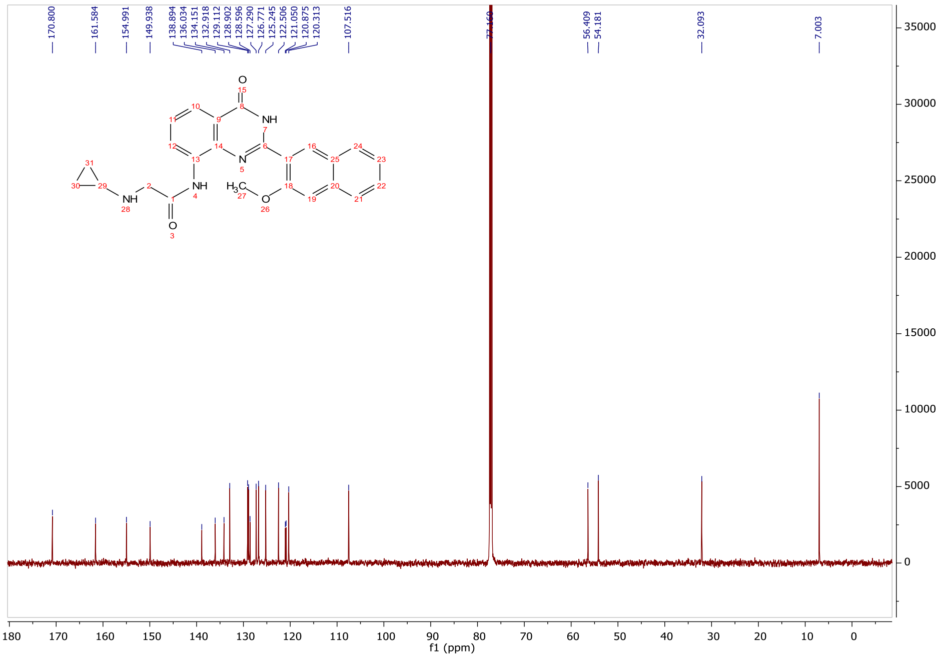

Figure S24: ^{13}C NMR spectrum of 20

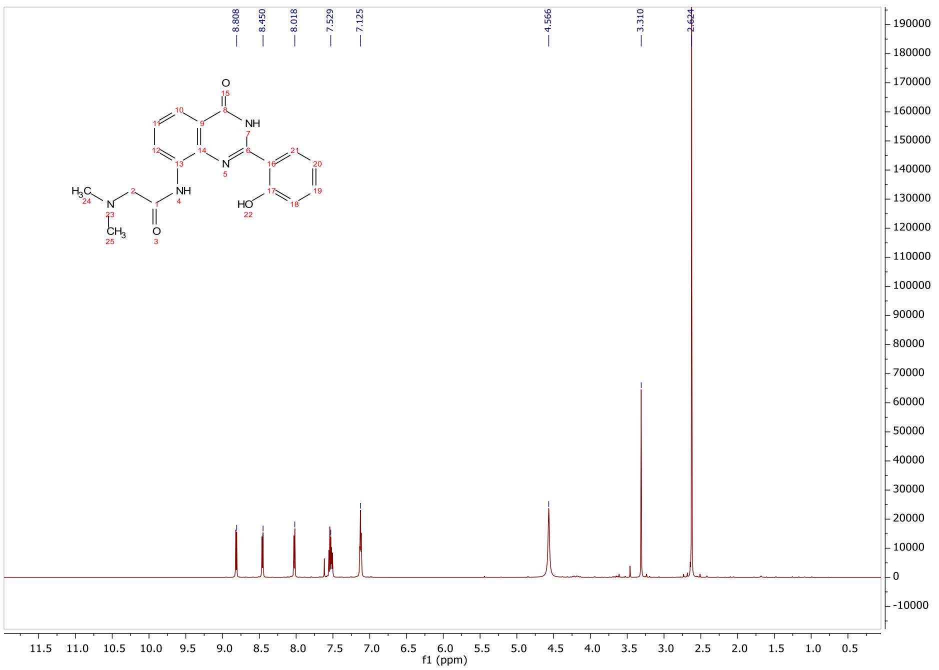

Figure S25: ^{1}H NMR spectrum of 21

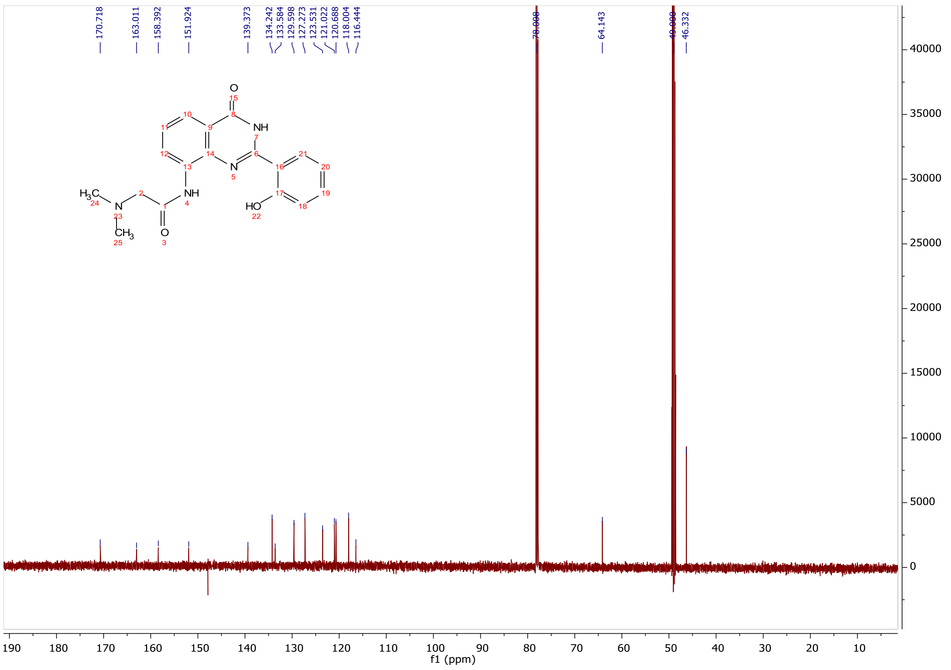

Figure S26: ^{13}C NMR spectrum of 21

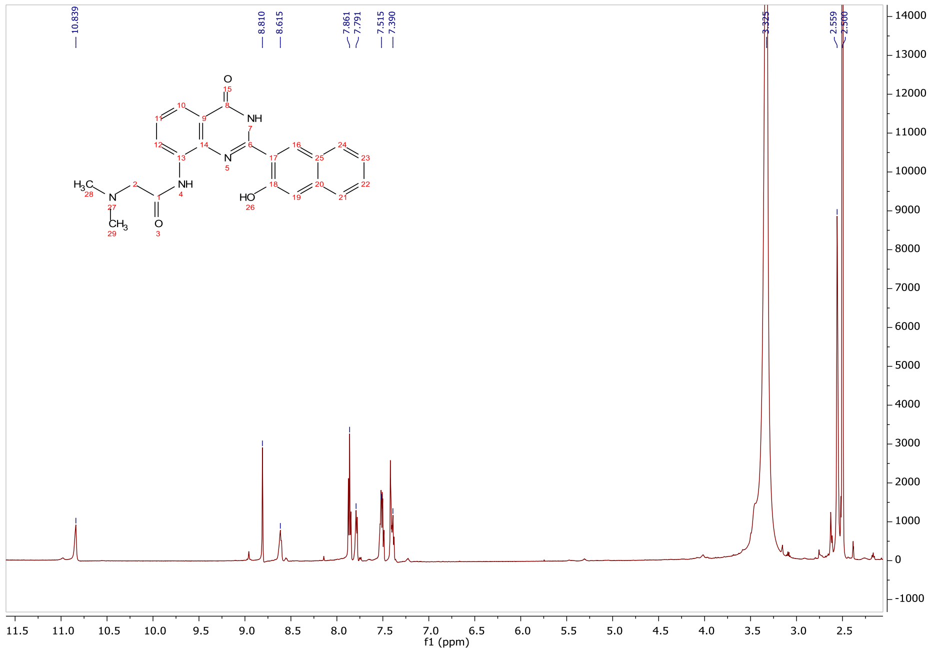

Figure S27: ^{1}H NMR spectrum of 22

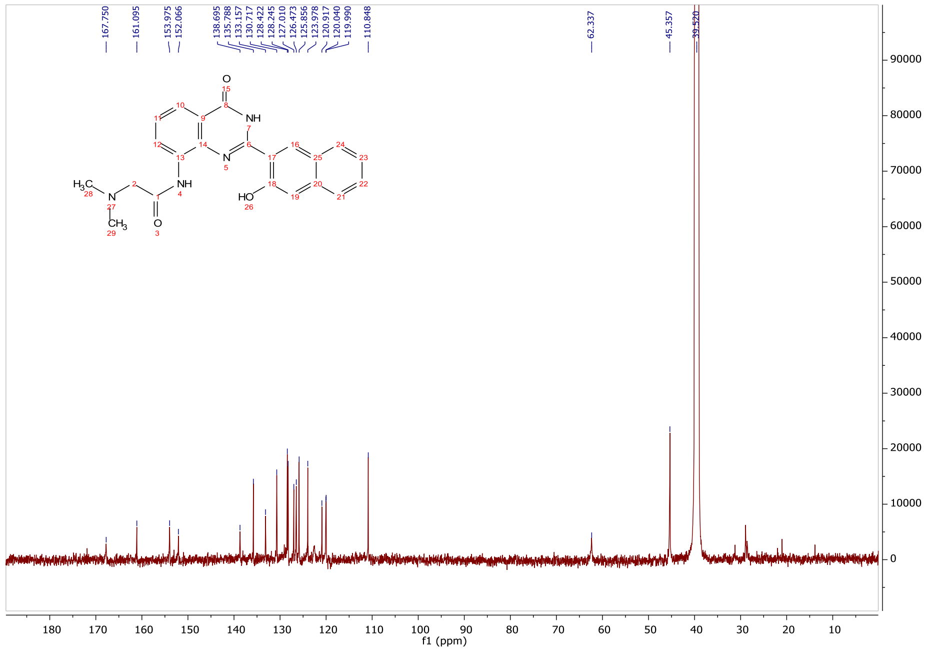

Figure S28: ^{13}C NMR spectrum of 22

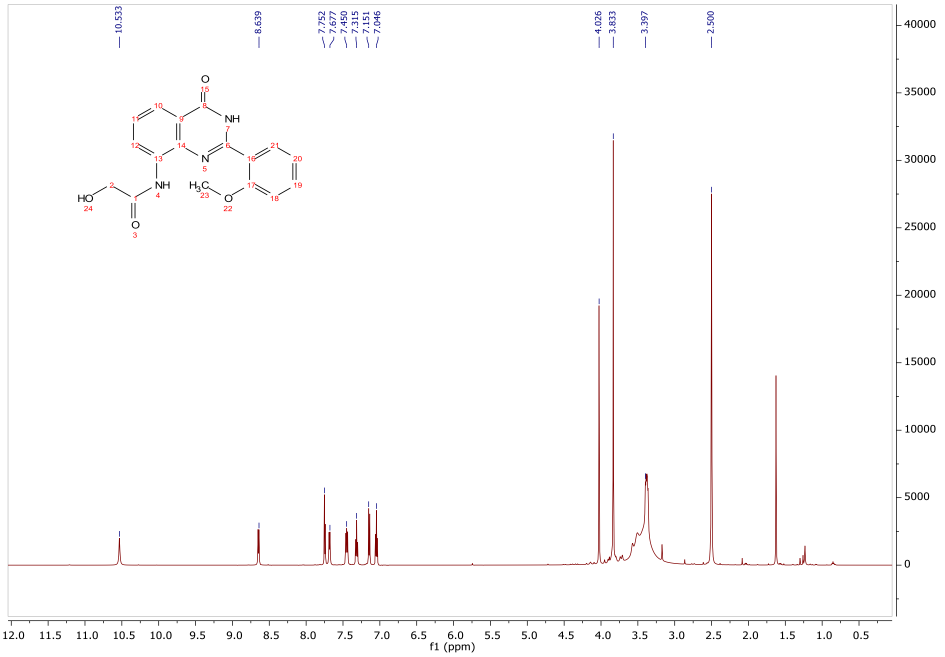

Figure S29: ^{1}H NMR spectrum of 25

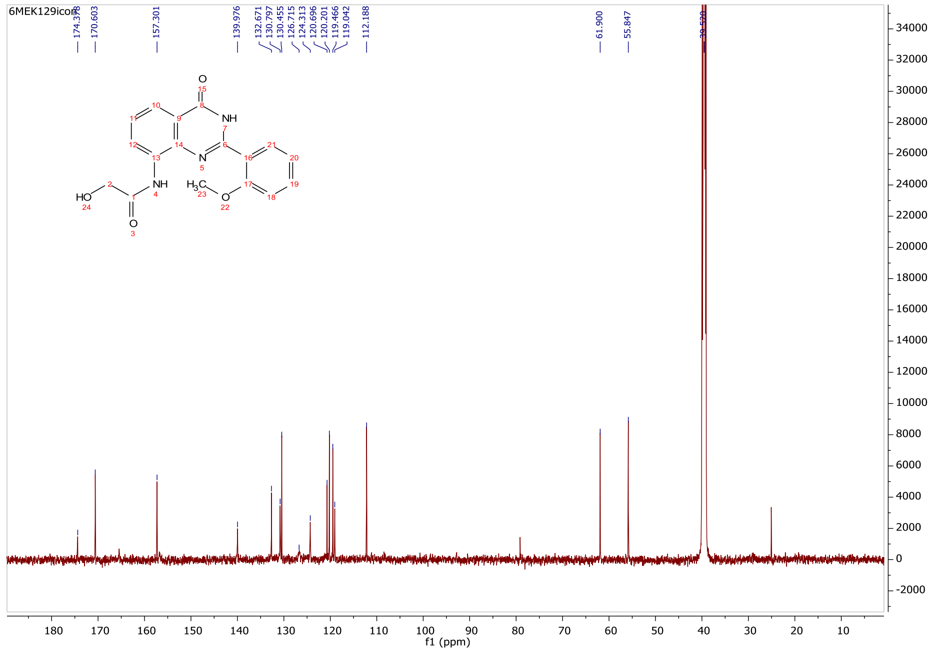

Figure S30: ^{13}C NMR spectrum of 25

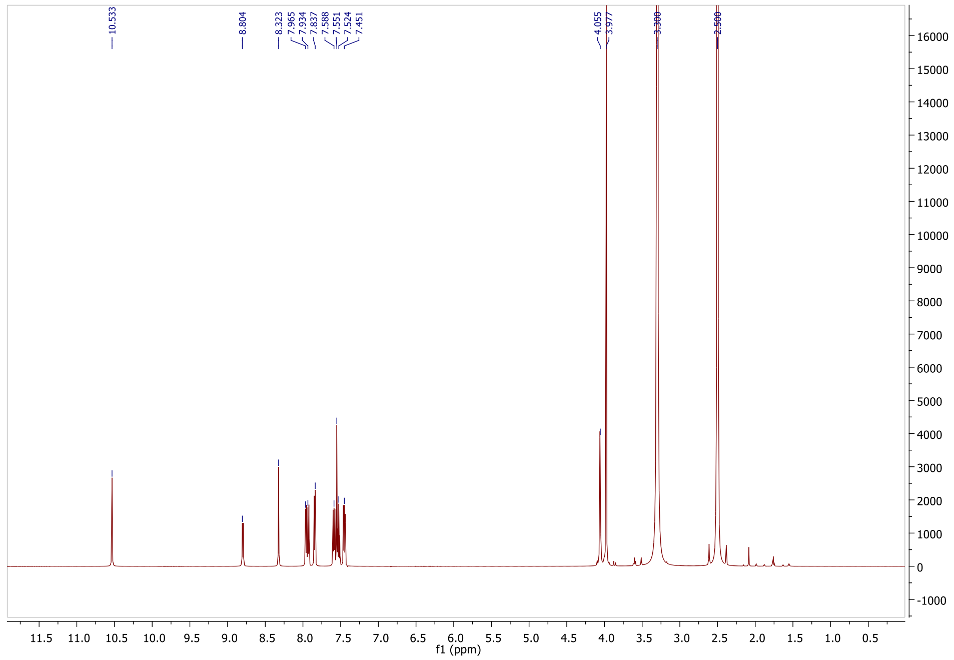

Figure S31: ^{1}H NMR spectrum of 26

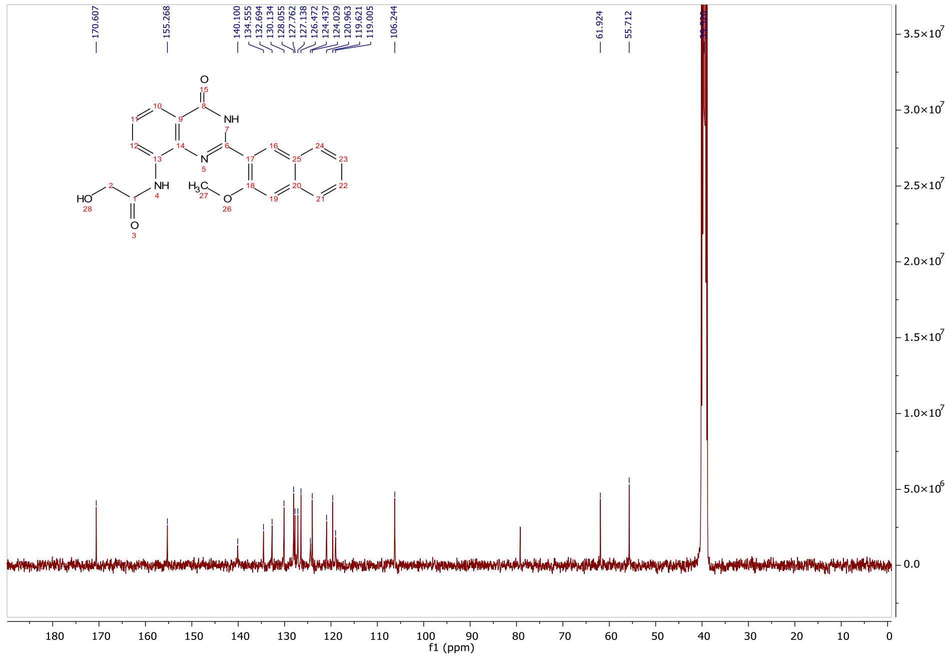

Figure S32: ^{13}C NMR spectrum of 26

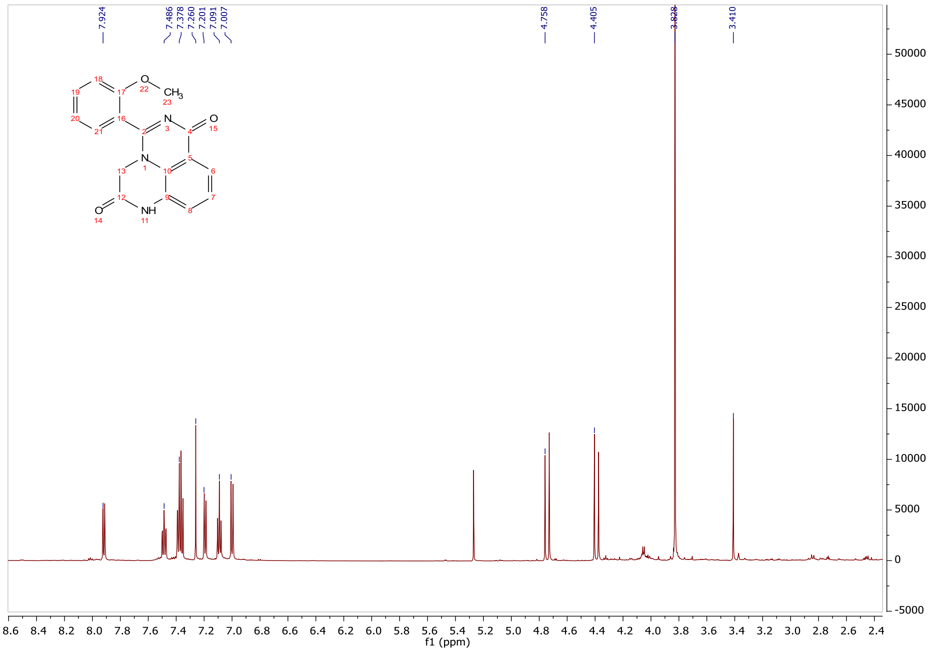

Figure S33: ^{1}H NMR spectrum of 27

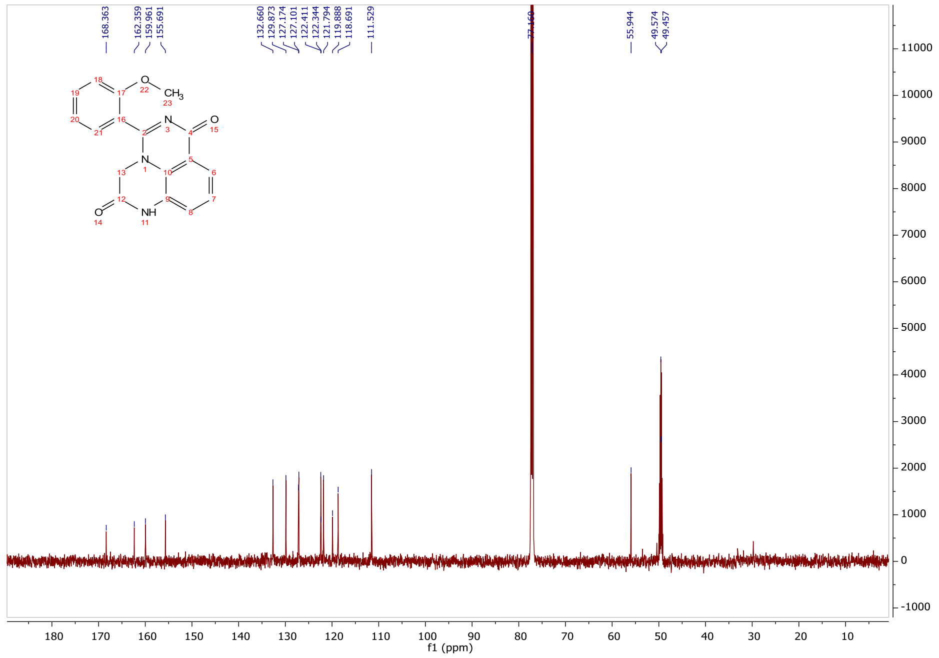

Figure S34: ^{13}C NMR spectrum of 27

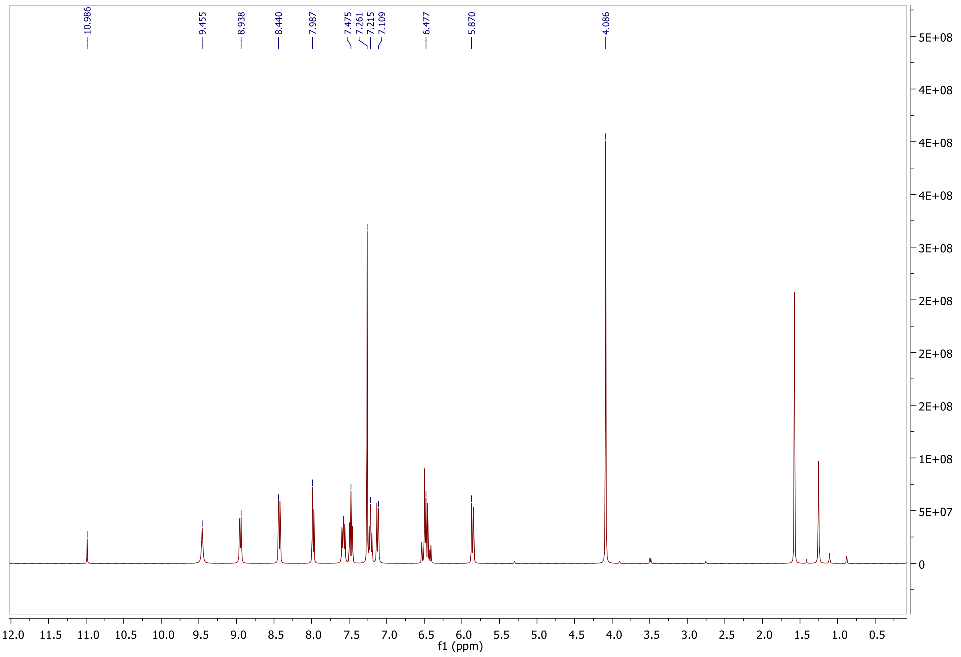

Figure S35: ^{1}H NMR spectrum of 28

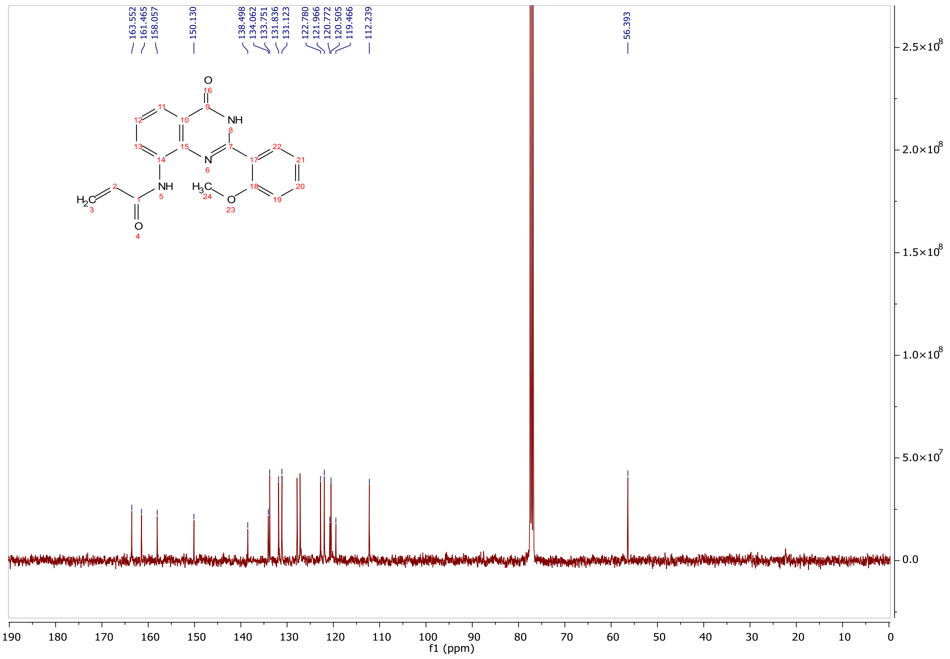

Figure S36: ^{13}C NMR spectrum of 28

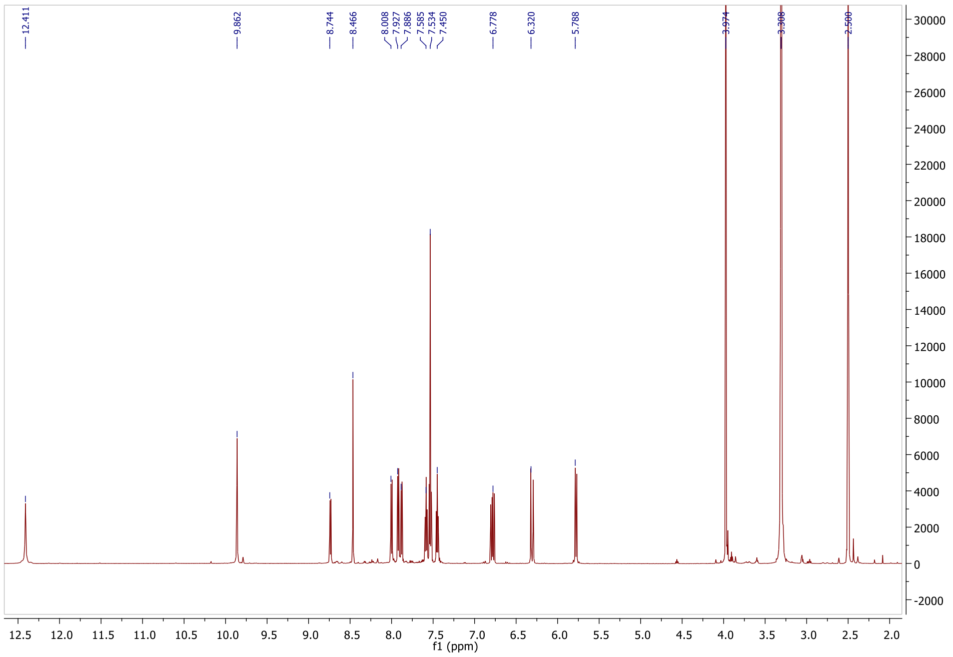

Figure S37: ^{1}H NMR spectrum of 29

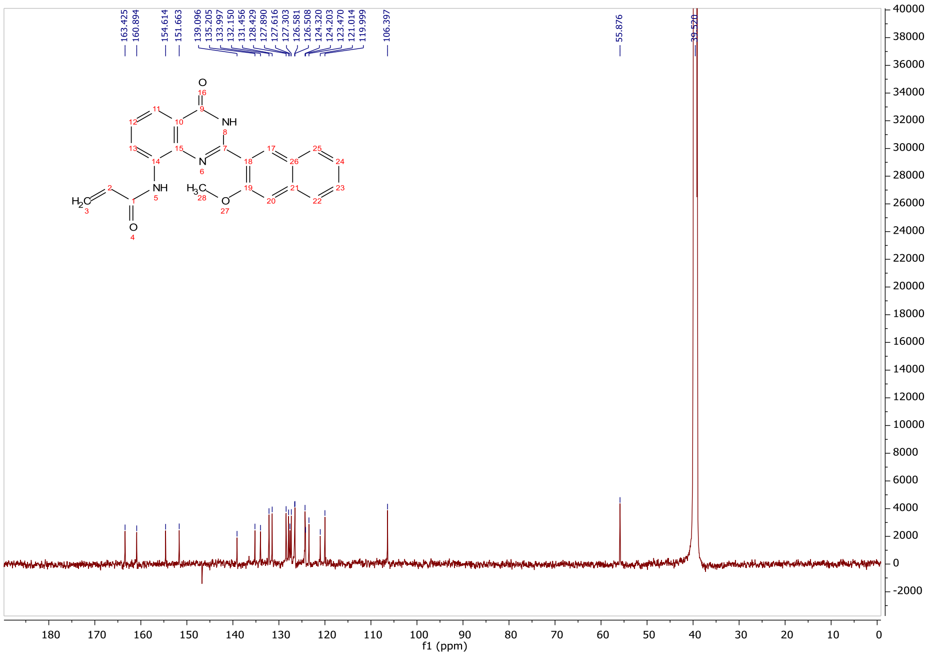

Figure S38: ^{13}C NMR spectrum of 29

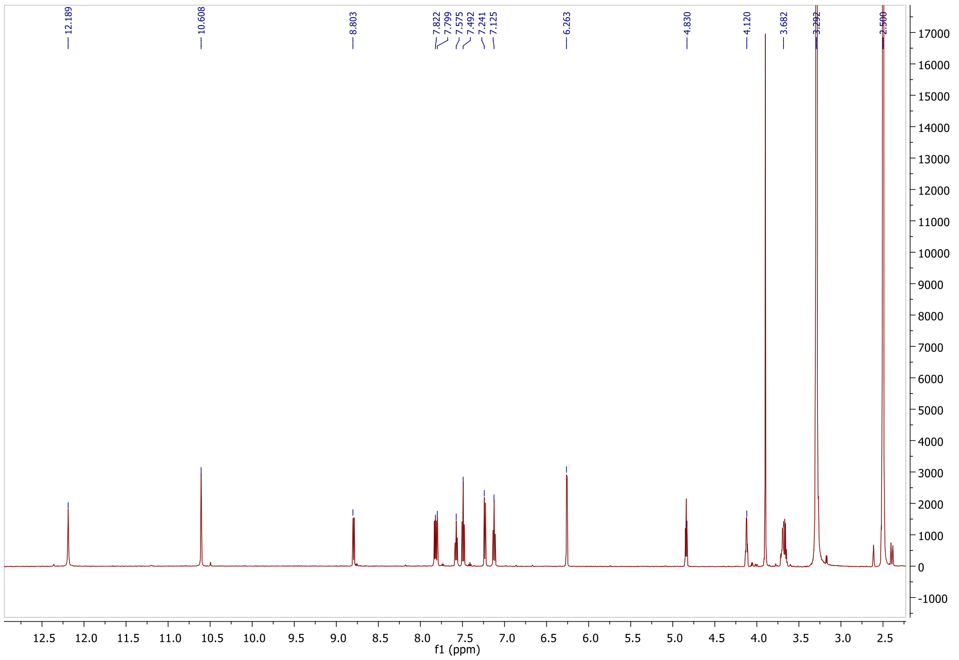

Figure S39: ^{1}H NMR spectrum of 30

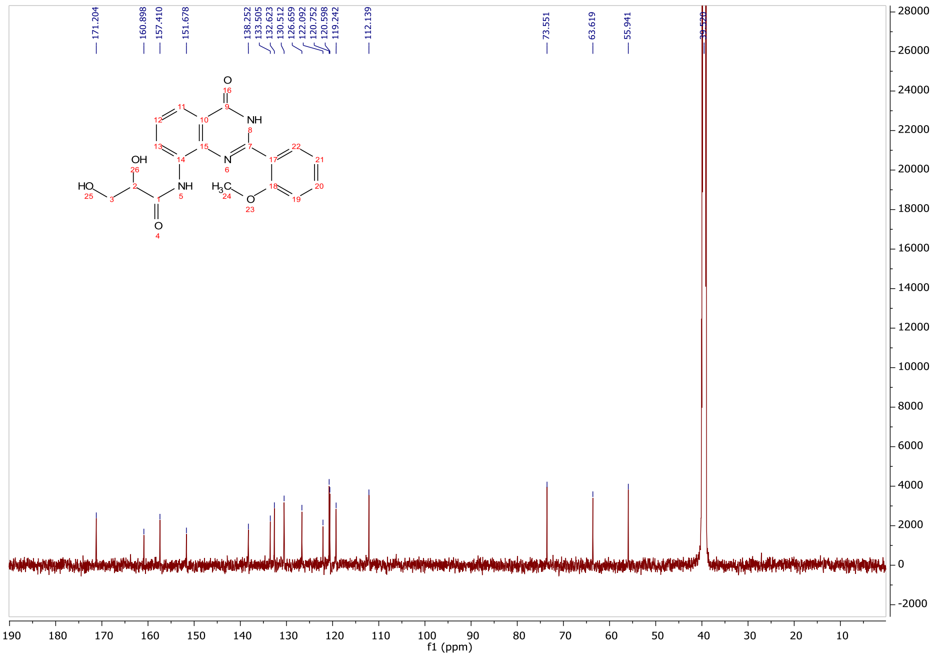

Figure S40: ^{13}C NMR spectrum of 30

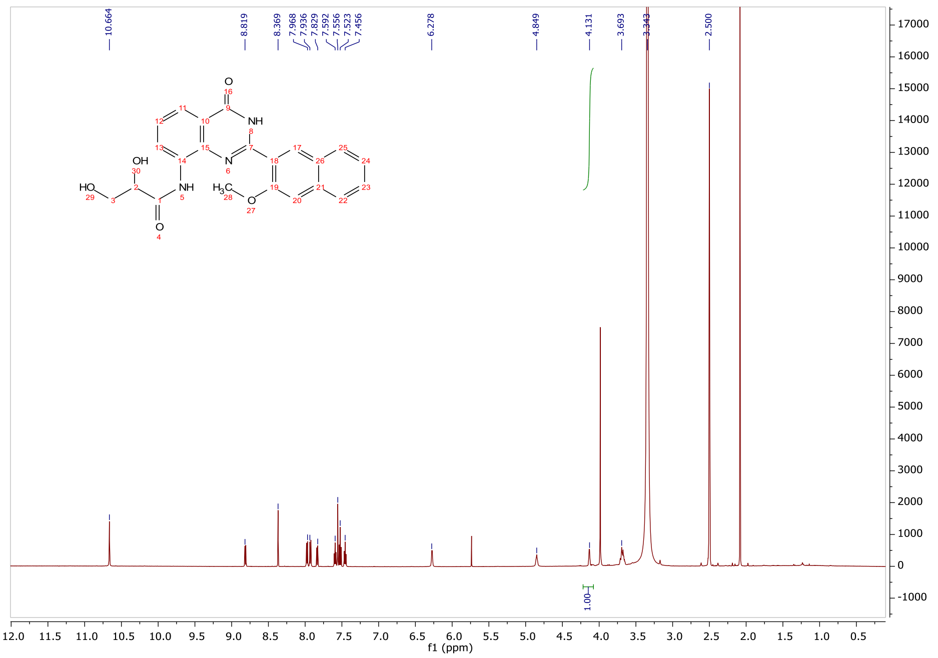

Figure S41: ^{1}H NMR spectrum of 31

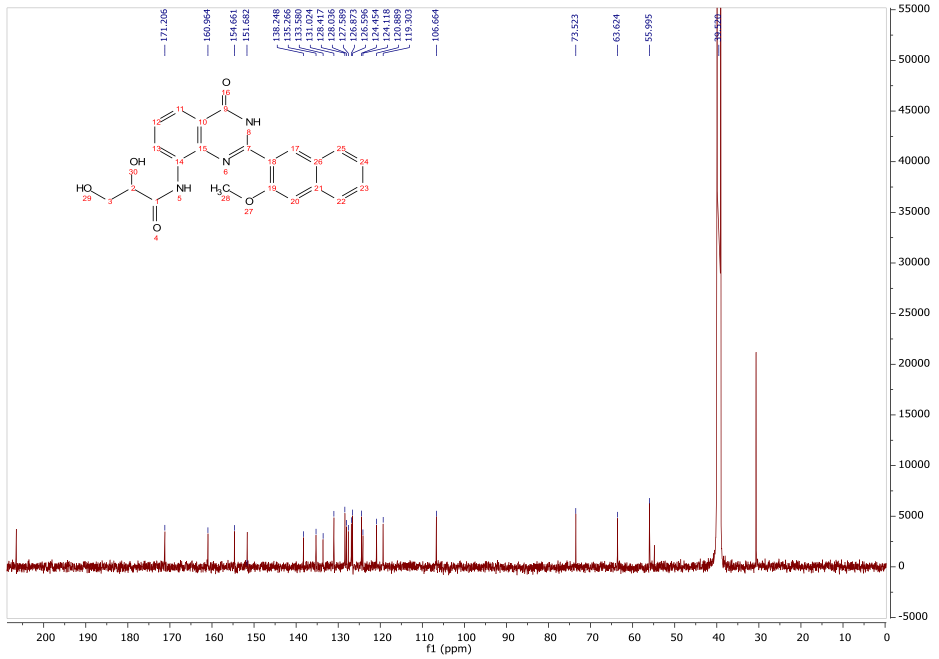

Figure S42: ^{13}C NMR spectrum of 31

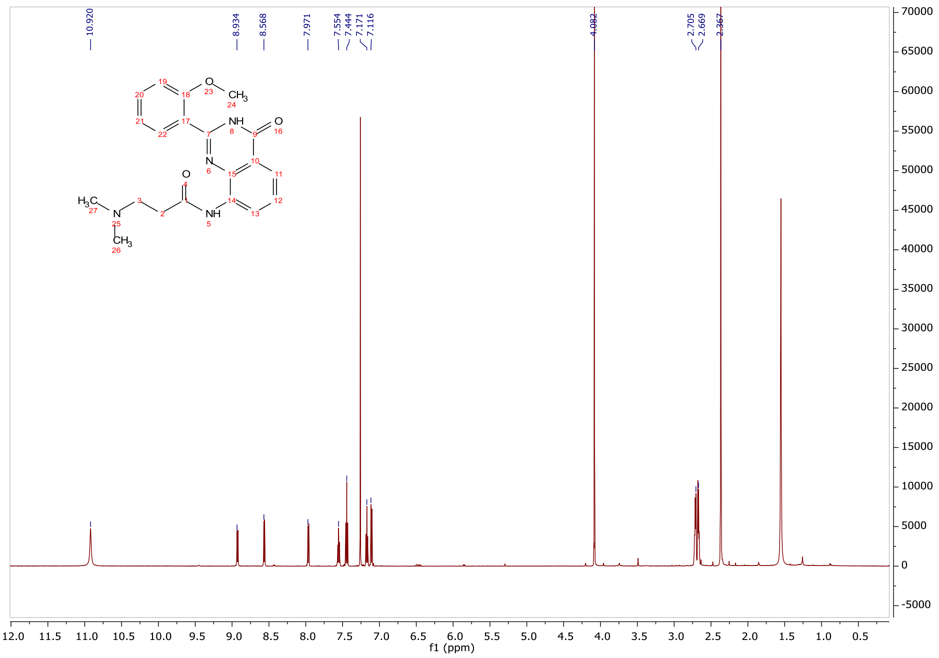

Figure S43: ^{1}H NMR spectrum of 32

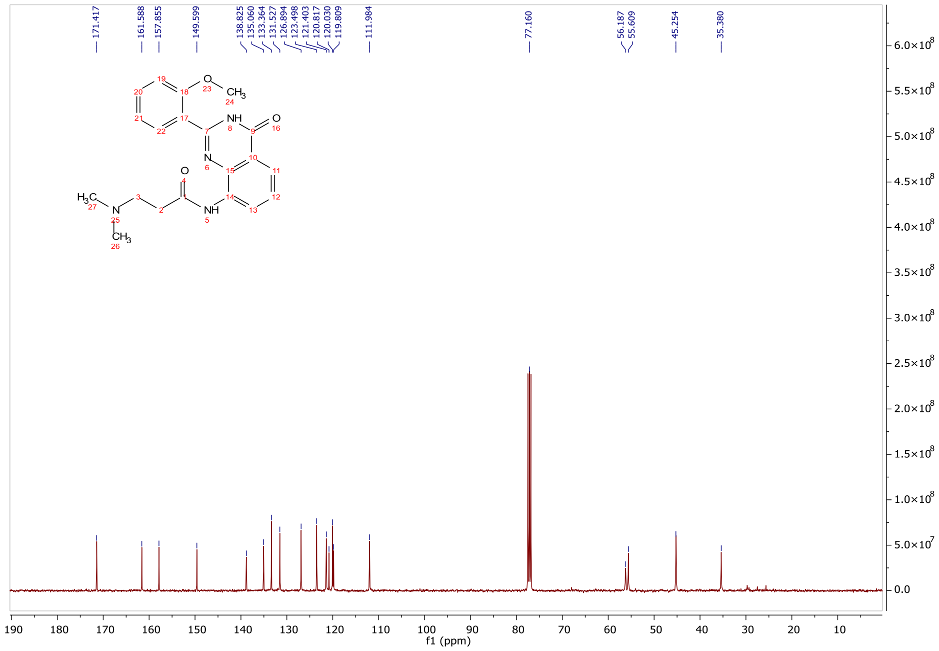

Figure S44: ^{13}C NMR spectrum of 32

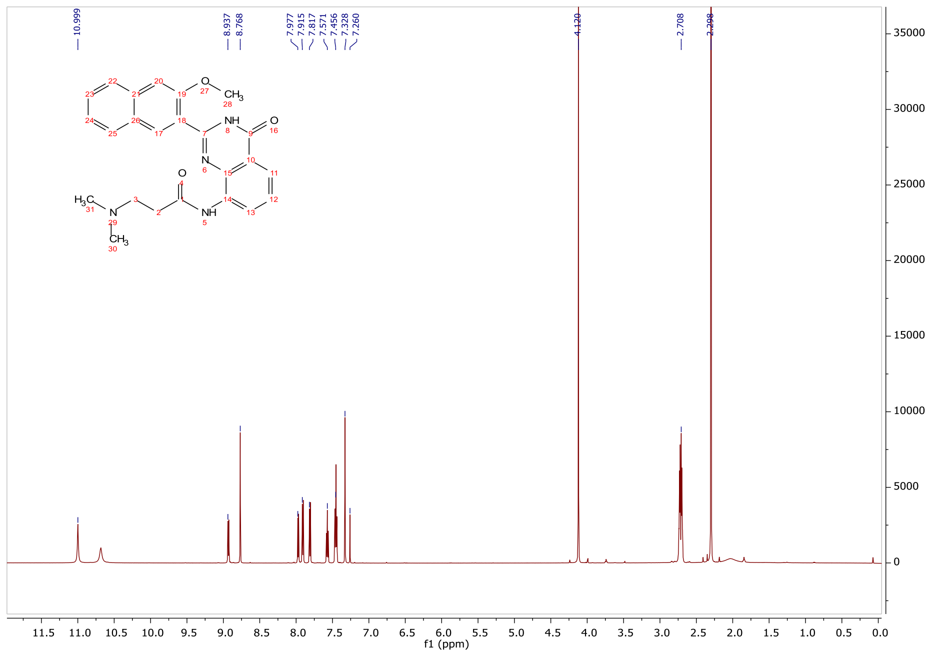

Figure S45: ^{1}H NMR spectrum of 33

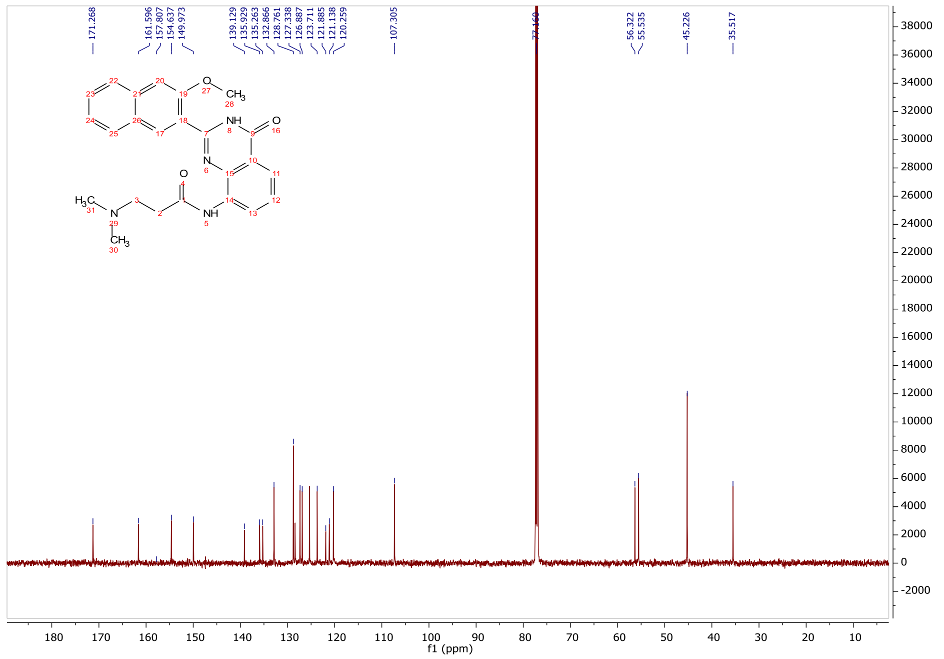

Figure S46: ^{13}C NMR spectrum of 33

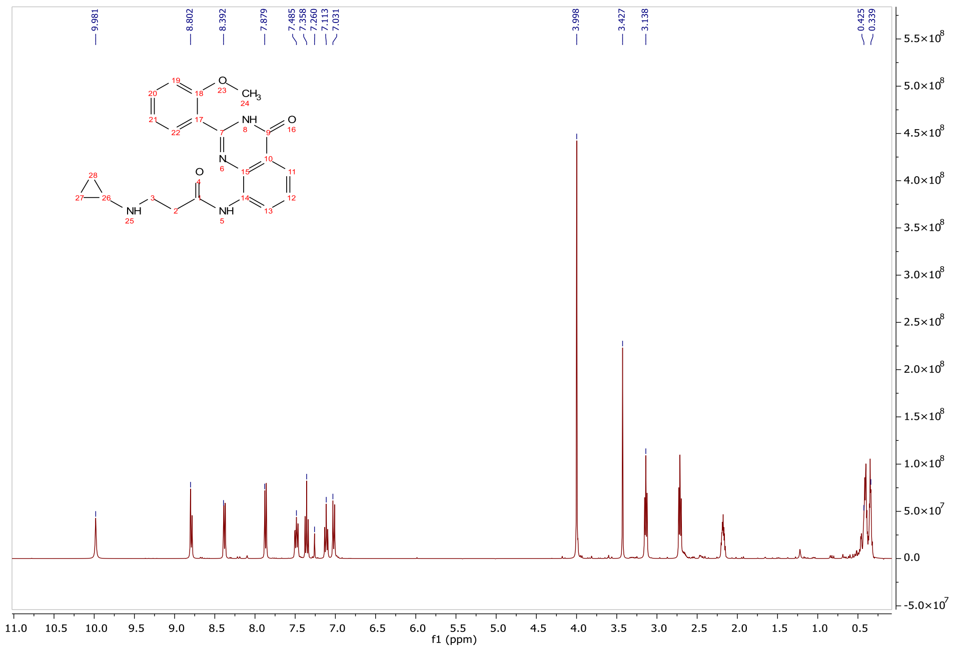

Figure S47: ^{1}H NMR spectrum of 34

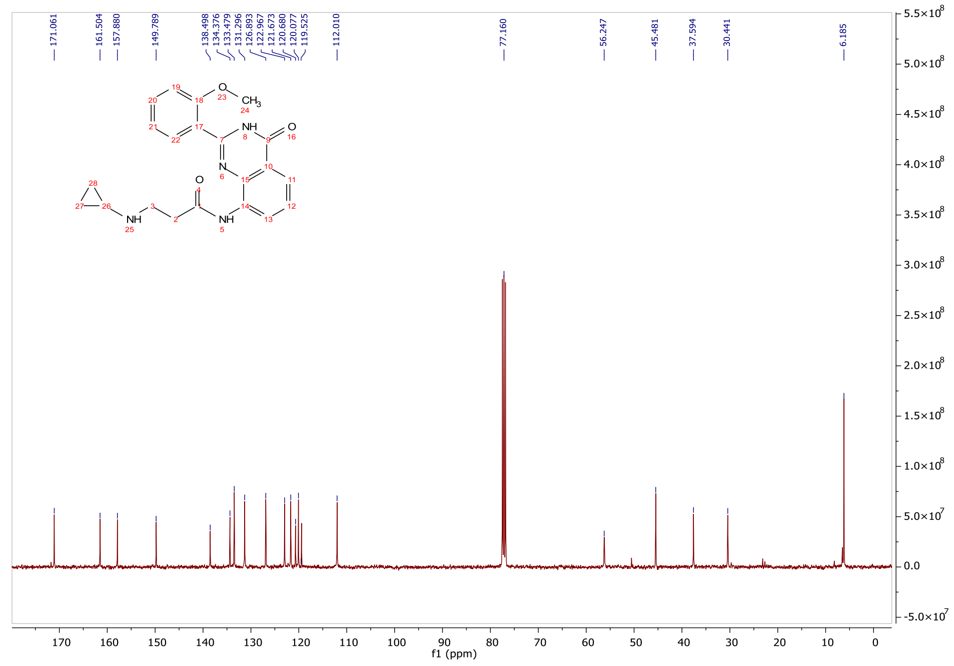

Figure S48: ^{13}C NMR spectrum of 34

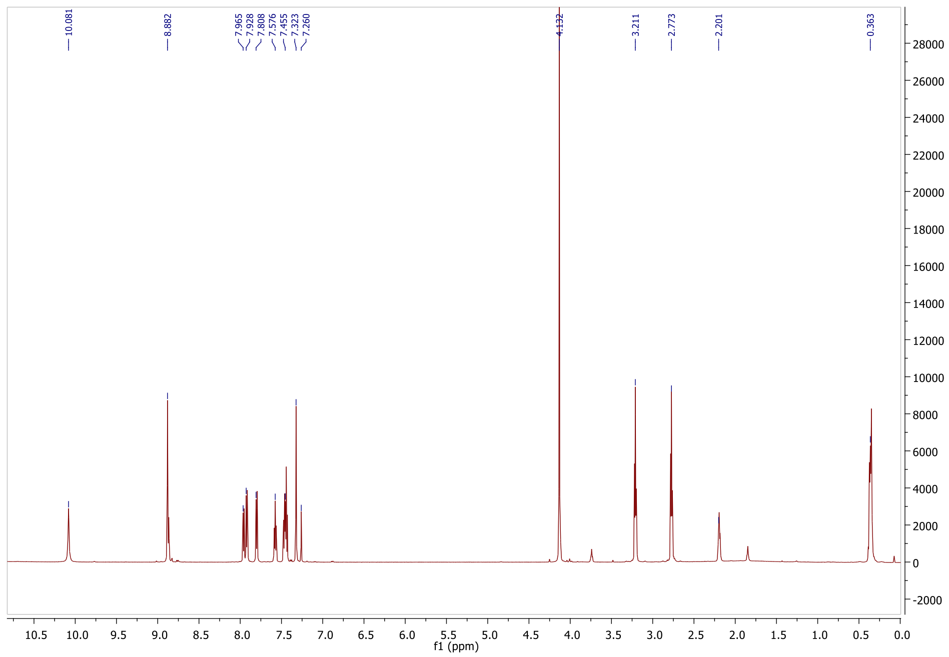

Figure S49: ^{1}H NMR spectrum of 35

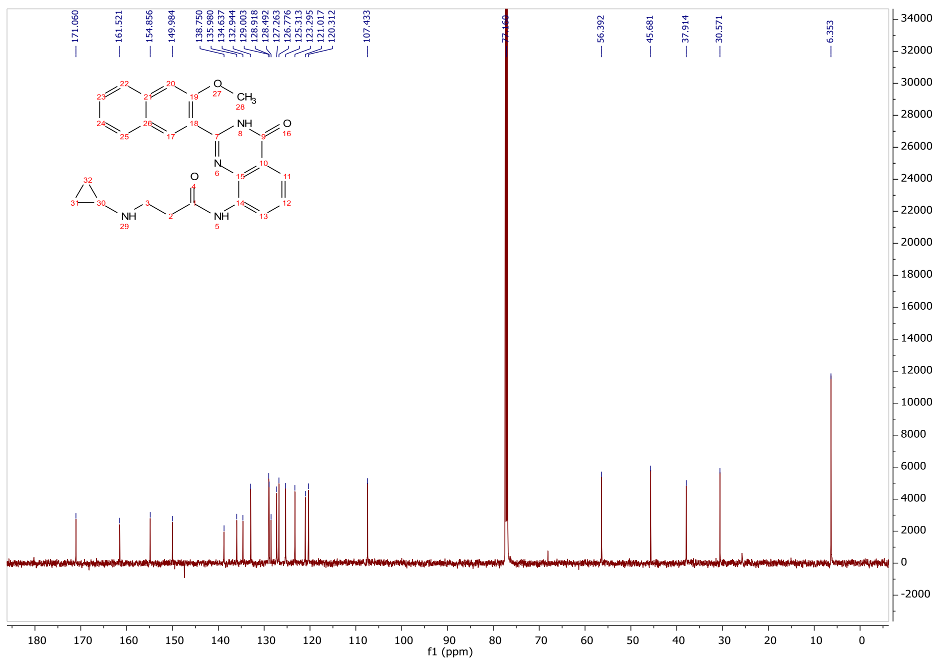

Figure S50: ^{13}C NMR spectrum of 35

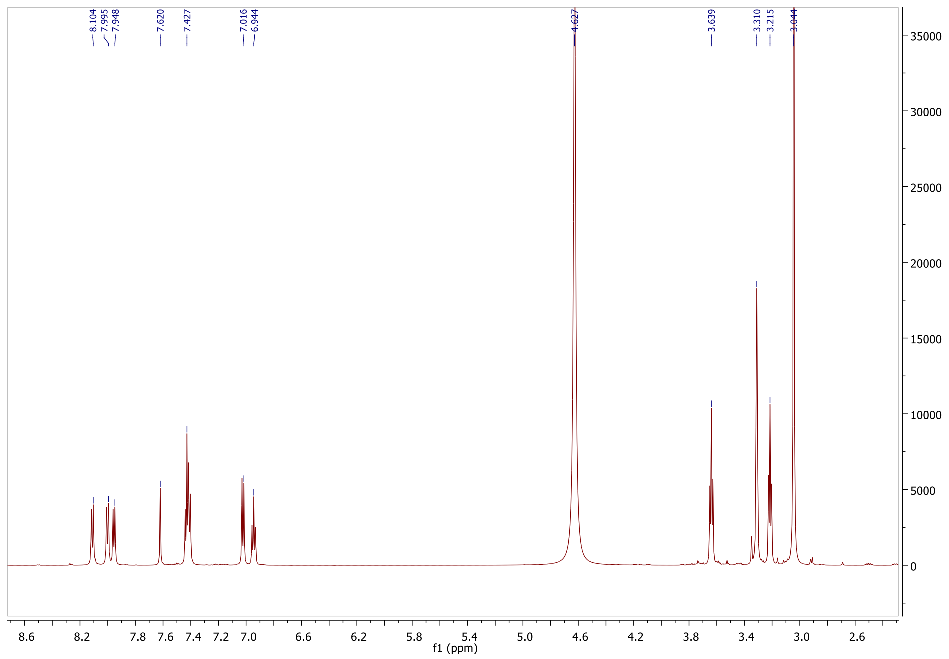

Figure S51: ^{1}H NMR spectrum of 36

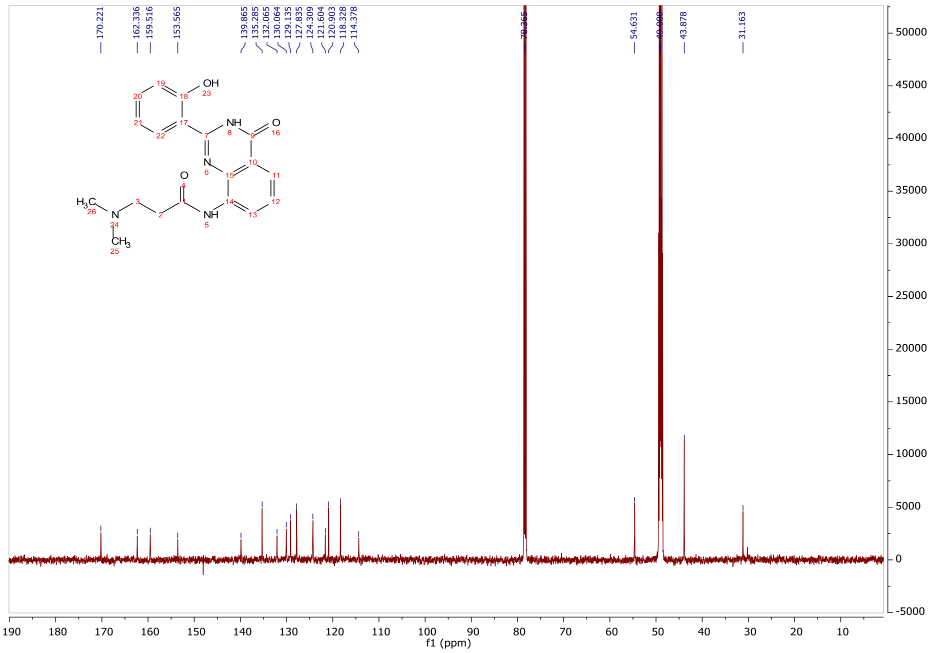

Figure S52: ^{13}C NMR spectrum of 36
